# Africans and Europeans differ in their facial perception of dominance and sex-typicality: a multidimensional Bayesian approach

**DOI:** 10.1038/s41598-022-10646-6

**Published:** 2022-04-26

**Authors:** Vojtěch Fiala, Petr Tureček, Robert Mbe Akoko, Šimon Pokorný, Karel Kleisner

**Affiliations:** 1 Department of Philosophy and History of Science, Faculty of Science, Charles University, Viničná 7, 128 44 Prague, Czech Republic; 2 Center for Theoretical Study, Charles University and Czech Academy of Sciences, Prague, Czech Republic; 3 Department of Communication and Development Studies, University of Bamenda, Bamenda, Cameroon

**Keywords:** Ecology, Evolution, Psychology

## Abstract

Biosocial impact of facial dominance and sex-typicality is well-evidenced in various human groups. It remains unclear, though, whether perceived sex-typicality and dominance can be consistently predicted from sexually dimorphic facial features across populations. Using a combination of multidimensional Bayesian approach and geometric morphometrics, we explored associations between perceived dominance, perceived sex-typicality, measured sexual shape dimorphism, and skin colour in a European and an African population. Unlike previous studies, we investigated the effect of facial variation due to shape separately from variation due to visual cues not related to shape in natural nonmanipulated stimuli. In men, perceived masculinity was associated with perceived dominance in both populations. In European women higher perceived femininity was, surprisingly, likewise positively associated with perceived dominance. Both shape and non-shape components participate in the constitution of facial sex-typicality and dominance. Skin colour predicted perceived sex-typicality in Africans but not in Europeans. Members of each population probably use different cues to assess sex-typicality and dominance. Using our methods, we found no universal sexually dimorphic scale predicting human perception of sex-typicality and dominance. Unidimensional understanding of sex-typicality thus seems problematic and should be applied with cautions when studying perceived sex-typicality and its correlates.

## Introduction

In a social group, some individuals are more prone to using violence and can more easily intimidate or defeat their rivals in status contests. Such individuals score high on behavioural dominance^1^. Behavioural dominance and dominance perceived by others are probably related^2,3^, which is also why perceived facial dominance may help identify individuals who are willing and capable of using strength, aggressive behaviour, and intimidation to impose their will on others.

In men, perceived facial dominance is associated with perceived and measured physical strength^4–6^ and with success in laboratory-controlled physical competitions^7^, whereby the perceived and actual threat potential–a composite measure derived from individuals’ upper-body strength, height, and weight–seem to be correlated^8^. In women, perceived strength and facial dominance are also associated^6^ and raters can predict female upper body strength from faces, albeit less accurately than in male facial stimuli^9^. This evidence suggests that perceived facial dominance serves as a cue to fighting ability and strength.

Facial features which are perceived as associated with dominance include low eyebrows, wide nose, relatively small (vertically short) eyes, and large chin^5,6,10^. These facial features are also regarded as masculine^11^ and various measures of facial masculinity are associated with perceived dominance in male faces^12–15^. Faces of women who attained higher scores in a questionnaire on personal dominance were likewise perceived as more masculine^3^. Similar to facial dominance, masculinity also serves as a cue to fighting ability. Measured facial masculinity in a combined sample of men and women^16^ as well as perceived facial masculinity in a sample of men^17^ were associated with hand-grip strength. In a forced-choice paradigm, perceived facial masculinity of facial stimuli also predicted actual fighting success of the depicted men^18^. Masculine and dominant features therefore seem related to each other and both function as cues to similar personal attributes, such as strength, threat potential, and fighting ability in both men and women.

Masculine/feminine (i.e., sex-typical) features and personal attributes reflect differences in sex hormone titres. In adult women, female sex-typical (feminine) facial traits are related to relatively higher oestrogens levels^19^ or a low testosterone-to-oestradiol ratio^20^. The development of masculine sex-typical traits, including craniofacial development, is directed by testosterone^21,22^, but facial masculinity in adults need not be associated with current testosterone levels^23^. On the other hand, current testosterone levels are associated with handgrip strength in adult men^24^ and with exercise and sports performance in adults of both sexes^25,26^. In young men, testosterone levels are also associated with aggressiveness^27^ (but see^28^ for a detailed review of the role of testosterone in modulating human aggression).

In sum, it has been proposed and substantiated by various studies that perceived facial masculinity and dominance are honest cues to testosterone-dependent strength and associated risk of threatening and formidable behaviour. This claim is based on current research, which, however, studies the phenomena using a variety of methods. Some studies investigate the association between measured facial sexual dimorphism and perceived dominance^11,29^ or association between measures of facial sexual dimorphism and success in dominance-based human hierarchies^30^. Other studies artificially masculinise or feminise facial stimuli and investigate whether such manipulation affects the perception of dominance and its correlates in the anticipated way^13,15,31,32^. This approach assumes that manipulation of facial configuration yields representative results and that relative change in facial shape is an important predictor of perceived sex-typicality and dominance. This, however, fails to consider further potential important predictors of the perceived characteristics and can be prone to methodological artifacts.

Besides facial shape, it has been established that skin colour also affects the perception of facial sex-typicality^33^. Redness increases perceived dominance^34,35^ and male faces are characteristically perceived as darker than female faces even when the male and female faces in question are set equally light (as measured by objective methods)^36^. Moreover, sex-typical facial shape and colour may affect the perception in opposing ways, i.e., not in concert. For example, male facial attractiveness is affected negatively by masculine facial shape and positively by masculine skin reflectance^37^.

There are several ways in which sexual dimorphism in faces can be measured^38–40^ and manipulated^41,42^. This is why the method of manipulation of sex-typical facial shape affects the results in studies on the perception of manipulated human facial sex-typicality, too^40,41^. Moreover, unlike measured facial sexual dimorphism, rated masculinity and femininity need not correspond to two halves or parallel subscales of a single bipolar dimension of sex-typicality^43^.

**Figure 1:**
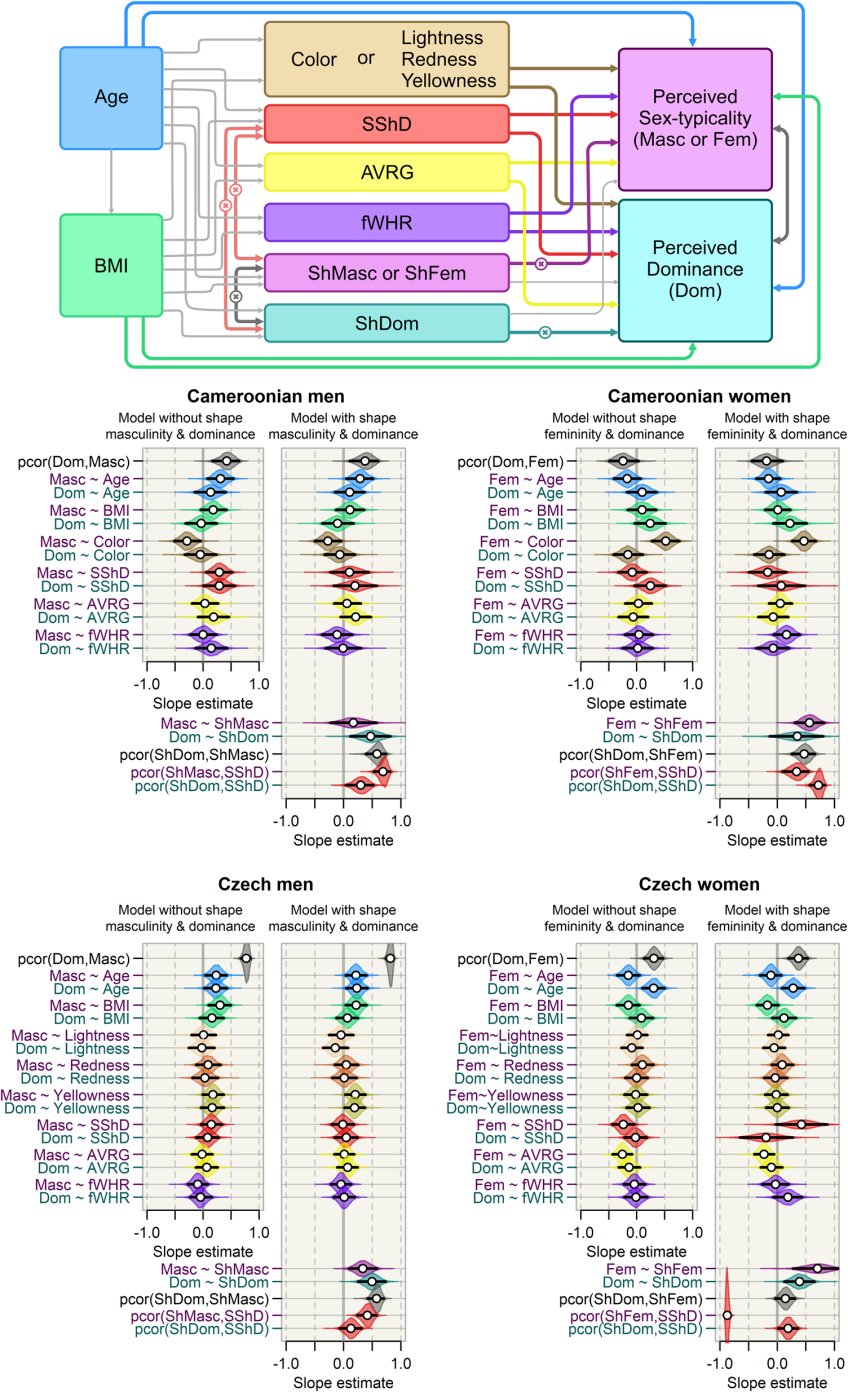
Model structure and density plots representing the posterior margins of selected coefficients (on a standardised scale) by sex and sample. In the upper part of the figure, we show the model structure where visualized regression and covariance parameters are depicted as thick arrows with colours corresponding to the respective density plots, while other unidirectional causal relationships are depicted by light grey arrows. All variables in the medium layer of variables (see the model structure) capturing facial colour and morphology are viewed as potentially correlated but shown are only partial correlations which appear in the parameter value distributions. Relationships which appear only in models with shape masculinity/femininity and dominance are marked with a cross ( ×). Four panels of density plots below the diagram of the model structure represent posterior margins for a given country and sex sample. BMI = body mass index; fWHR = facial width to height ratio, SShD = sexual shape dimorphism, DIST = morphological distinctiveness; L*, a*, b* = lightness, redness, yellowness (CIELab L*a*b*); Masc/Fem = perceived sex-typicality (masculinity of men/femininity of women); Dom = perceived dominance; ShDom = shape dominance; ShMasc = shape masculinity; ShFem = shape femininity. Black error bars span the 95% compatibility intervals of the parameters. The complete posterior summary can be found in Supplementary Fig. S4.

In view of these apparent inconsistencies and contradictions, we explored the simultaneous effect of facial sexual shape dimorphism (SShD) and skin colour on perceived sex-typicality and dominance. Men were rated on masculinity, women on femininity; jointly, we call these ratings ‘perceived sex-typicality’. We also considered the effects of age, relative facial width, relative body mass, and measured the distinctiveness of facial shape. Further, we explored the non-directed association between perceived sex-typicality and perceived dominance (see Fig. 1). Extending this default analysis, we further calculated individual scores of the shape component of perceived sex-typicality and dominance by regressing facial shape coordinates on perceived dominance and sex-typicality separately. Two variables which emerged from this approach, namely ‘shape dominance’ and ‘shape sex-typicality’, were subsequently entered into expanded models. This allowed us to separate the effect of facial shape from other possible non-shape traits-such as skin coloration, contrast, and texture-and explore the effect of the shape component of perceived characteristics independently of non-shape facial features. We used the shape components of the perceived scales (‘shape dominance’ and ‘shape sex-typicality’) and perception-independent sexual shape dimorphism (SShD) simultaneously in a single analysis. This allowed us to explore how different shape-related variables explain the variability of facial perception. It also enabled us to investigate residual non-directed bivariate associations between various facial shape-derived sexually dimorphic variables (predictors).

To lower the risk of reported associations being the artifacts of collinearity between predictors, we also fitted models with SShD and either ‘shape dominance’ or ‘shape sex-typicality’. These partial models enabled us to explore the effects of different shape-derived predictors both on each other and on the dependent variables.

In a ‘unidimensional understanding’ of sexual dimorphism^43^, all measured and perceived sexually dimorphic scales would be located in parallel on a single bipolar scale corresponding to general masculinity–femininity. But given previous research^38,43^, it is reasonable to expect that different sexually dimorphic scales are in fact not parallel to each other. Using our data on perceived male masculinity and female femininity, we tried to avoid the risk of gender-biased perception, which may affect ratings of male femininity and female masculinity; see^44^. Following this decision, we could not directly identify the dimensionality of sex-typicality space or calculate the angle between feminine and masculine subscale of sex-typicality scale(s). We could, however, test two predictions that would present indirect but convincing evidence for the hypothetical bipolar masculinity–femininity scale:

- (H1A) Women’s faces perceived as most feminine should have the most feminine (shape femininity) and female-like (SShD) facial shape and skin colour, i.e., would be relatively lighter. Men’s faces perceived as most masculine should have the most masculine (shape masculinity) and male-like (SShD) facial shape and skin colour, i.e., be relatively darker.
- (H1B) Perceived dominance should be predicted in the same direction: more masculine and male-like facial shape, relatively darker skin colour, and higher perceived masculinity would be perceived as more dominant. More feminine and female-like shape, relatively lighter skin colour, and higher perceived femininity should be perceived as less dominant.

The data would thus present indirect evidence for a single underlying sex-typicality scale, where female faces perceived as the most feminine, with the most female-like shape and relatively lighter colour features, receive the lowest dominance ratings, while relatively darker, male-like shaped, and masculine-rated male faces are rated as most dominant.

Moreover, we explore whether these predictions are cross-culturally invariant. Our study took place in two distinctive populations, one African and one European. According to recent evidence, African faces have a relatively lower range of facial SShD compared to Europeans, who have more dimorphic faces^45^. On the other hand, skin colour seems to affect the perceived characteristics of African faces (as perceived by Africans) more than is the case for European faces^46–48^.

We therefore expect (H2) that in Cameroonian (African) raters, skin colour would predict perceived dominance and sex-typicality more than in our Czech (European) sample, where we expect it to be relatively unimportant. On the other hand, variance in facial shape would affect the ratings relatively more in the Czech sample.

Finally, Cameroon can be regarded as a non-WEIRD (Western, Educated, Industrialized, Rich, and Democratic, see^49^) population, which makes local data especially important, because any association that emerges in both samples is a good candidate for being stable across different populations.

## Material and methods

In Czechia, this study was approved by the Institutional Review Board of Charles University, Faculty of Science (approval number 2020/4). In Cameroon, data collection was approved by the means of a Research Permit provided by the Division of Scientific Policy and Planning, Ministry of Scientific Research and Innovation, Cameroon. Experiment protocols were in accordance with the Declaration of Helsinki and related guidelines. Informed consent was obtained from all participants. Photographed individuals signed informed consent and raters consented by clicking ‘I agree’ in the questionnaire. All data, scripts, as well as detailed descriptions of stimuli acquisition, stimuli ratings, and subsequent statistical analyses are available at https://osf.io/mqgxa/?view_only=a42db3ea5d0f4bd3b76e4d614575ba92. Supplementary figures, tables, and detailed methods descriptions are also located in the Supplementary Materials with this article.

### Participants

#### Acquisition of stimuli

We collected the Cameroonian photographic stimuli in 2013 at the University of Buea (Southwest Region of Cameroon). Czech stimuli were collected at the Charles University in Prague in 2016 and 2019. Participants were recruited by direct invitation, via social networks, and using fliers displayed around the campus of the respective universities.

Acquisition of facial portraits followed a standardised procedure^50^. Participants were instructed to remove facial cosmetics, jewellery, and other decorations (if possible). They wore standardised clothes (black T-shirt) and were asked to adopt a neutral facial expression. Detailed information about the acquisition of facial portraits is available in Supplementary Materials.

In all three sets, most participants were undergraduate university students. Cameroonian participants received 5,000 CFA as a remuneration for their participation (equivalent of app. € 8). Czech participants received either CZK 200 (app. 7 €) or a bottle of wine.

The counts of stimuli and their descriptive statistics are in Table 1 and Table S1 in online supplementary material.

**Table 1:** The counts of stimuli and their descriptive statistics.

Sample	N	Variable	Mean	SD	Min	Max	Skewness	Kurtosis
CMR women	50	Age	21.24	1.89	17.00	25.00	− 0.23	2.94
CZ women	106		23.09	4.12	19.00	39.00	1.49	5.19
CMR women	50	BMI	24.30	4.17	18.59	35.82	0.91	3.35
CZ women	106		21.80	2.89	17.34	38.11	2.24	12.40
CMR women	50	fWHR	2.16	0.17	1.85	2.75	0.86	4.58
CZ women	106		1.93	0.12	1.60	2.28	0.05	3.23
CMR women	50	SShD	− 0.01	0.01	− 0.04	0.02	− 0.12	2.21
CZ women	106		− 0.02	0.02	− 0.07	0.03	0.07	3.24
CMR women	50	DIST	0.05	0.01	0.03	0.08	0.41	2.62
CZ women	106		0.06	0.01	0.03	0.11	0.55	3.51
CMR women	50	L*	38.36	6.45	27.11	55.50	0.28	2.64
CZ women	106		61.38	3.46	53.90	82.17	1.79	14.02
CMR women	50	a*	21.09	3.18	13.14	25.60	− 0.67	2.75
CZ women	106		17.39	2.50	11.58	25.66	0.86	4.53
CMR women	50	b*	18.13	4.45	8.23	26.94	− 0.29	2.45
CZ women	106		13.12	2.14	8.58	20.80	0.89	4.75
CMR women	50	Perceived Femininity	4.88	0.58	3.60	5.78	− 0.39	2.39
CZ women	106		4.00	0.84	1.64	5.80	− 0.54	3.02
CMR women	50	Perceived Dominance	3.90	0.49	2.67	4.91	− 0.27	2.93
CZ women	106		3.81	0.66	2.05	5.32	0.12	2.78
CMR women	50	Shape Dominance	0.0000	0.0005	− 0.0011	0.0009	− 0.06	2.52
CZ women	106		0.0000	0.0002	− 0.0005	0.0004	− 0.28	3.22
CMR women	50	Shape Femininity	0.0000	0.0001	− 0.0003	0.0002	− 0.33	2.43
CZ women	106		0.0000	0.0001	− 0.0004	0.0004	− 0.05	3.98
CMR men	49	Age	22.00	2.24	17.00	30.00	0.56	4.87
CZ men	89		23.38	4.25	19.00	43.00	1.74	7.16
CMR men	49	BMI	23.15	2.33	17.01	30.93	0.78	4.97
CZ men	89		22.99	2.36	16.27	28.60	− 0.08	3.10
CMR men	49	fWHR	2.09	0.17	1.77	2.45	0.35	2.43
CZ men	89		1.88	0.11	1.61	2.27	0.59	4.48
CMR men	49	SShD	0.01	0.01	− 0.03	0.04	− 0.50	3.20
CZ men	89		0.02	0.01	− 0.03	0.05	− 0.08	3.38
CMR men	49	DIST	0.05	0.01	0.03	0.08	0.41	2.78
CZ men	89		0.06	0.01	0.03	0.09	0.73	3.21
CMR men	49	L*	32.91	6.24	20.51	49.08	0.36	2.78
CZ men	89		58.77	2.74	52.64	67.95	0.39	3.65
CMR men	49	a*	17.44	3.41	10.85	25.00	0.38	2.64
CZ men	89		19.15	2.66	10.76	26.59	− 0.31	3.42
CMR men	49	b*	13.33	4.33	5.86	24.96	0.75	3.37
CZ men	89		13.72	1.57	10.14	17.78	0.39	2.88
CMR men	49	Perceived Masculinity	5.62	0.57	3.78	6.48	− 1.03	4.30
CZ men	89		4.17	0.85	2.19	6.39	0.09	2.84
CMR men	49	Perceived Dominance	4.11	0.51	3.11	5.47	0.55	3.39
CZ men	89		3.94	0.71	1.97	5.95	0.23	3.45
CMR men	49	Shape Dominance	0.0000	0.0002	− 0.0007	0.0004	− 0.57	3.46
CZ men	89		0.0000	0.0001	− 0.0002	0.0002	− 0.06	2.60
CMR men	49	Shape Masculinity	0.0000	0.0003	− 0.0008	0.0005	− 0.36	2.88
CZ men	89		0.0000	0.0001	− 0.0003	0.0002	− 0.06	2.76

*BMI* body mass index, *fWHR* facial width-to-height ratio, *SShD* sexual shape dimorphism, *DIST* morphological distinctiveness (distance from mean sample configuration), L*, a*, b* lightness, redness, and yellowness dimension (respectively) of CIELab colour space; Shape Dominance and Shape Masculinity/Femininity = shape component (shape variance) of ratings of stimuli dominance and sex-typicality (Masculinity/Femininity), *CMR* Cameroon, *CZ* Czech Republic, *SD* standard deviation.

#### Processing of stimuli and rating collection

Before being presented to raters, facial photographs were colour-checked and corrected in Adobe Photoshop Lightroom. X-Rite ColourChecker white card (Passport Photo 2) was used as a reference for white balancing and exposure calibration in Adobe Photoshop Lightroom 4. Position of the face in the image was adjusted so as to horizontally align the pupils, leave a standard length of the neck visible, and leave roughly the same amount of space above the hair of the depicted person. For the collection of ratings, photographs were resized to a lower resolution (~ 500 × 700 px). No other shape or colour manipulations were applied to the photos.

We collected the ratings separately for each photoset and recruited raters from local populations. Raters who rated dominance were not aware of the sex-typicality ratings and raters who rated sex-typicality were not aware of the dominance ratings. Rating took place using electronic devices and online survey software Qualtrics (www.qualtrics.com). Raters were briefed about the purpose of the study and answered demographic questions concerning their age, weight, height, and size of their place of residence. Then they were instructed to rate a set of stimuli on a seven-point rating scale anchored with 1 = not at all feminine/masculine/dominant, 7 = very much feminine/masculine/dominant. Male stimuli were rated on masculinity, female stimuli on femininity. In this study, ratings thus delivered ‘perceived sex-typicality’ and ‘perceived dominance’. The rating procedure was self-paced, and the order of stimuli was (pseudo)randomised. Raters were asked to rate a face on the rating scale (e.g., dominance) that was not further defined (e.g., by describing how dominance should be assessed, supplying readers with a text about dominance perception and its hypothetical association with dominant behaviour, etc.). We aimed to get ratings that mirror raters subjective understanding of a given scale. Dominance and sex-typicality ratings were collected in separate questionnaires. Cameroonian raters rated only the Cameroonian stimuli and Czech raters rated only the Czech stimuli.

In the Cameroonian questionnaires, we used only answers from raters who confirmed their Cameroonian origin. In Czech questionnaires, we used answers provided by raters of both Czech and Slovak nationality due to general cultural and linguistic similarity between Czechs and Slovaks^51^.

In contrast to other datasets (i.e., Cameroonian datasets and Czech 2019 datasets) where men and women rated facial photos of both men and women, in the rating of sex-typicality of the Czech 2016 stimuli set photographs of men were rated only by women and photos of women were only rated by men.

The counts and descriptive statistics of raters are available in Table S2 in online Supplementary Materials and at (https://osf.io/mqgxa/?view_only=a42db3ea5d0f4bd3b76e4d614575ba92). Across all datasets, stimuli were rated by 44–231 raters (mean ± SD = 102.3 ± 58.1). The average age of all raters was 23–35 years and interrater agreement was high (ICC 3, k ≥ 0.91).

### Facial measures, body measure

#### Relative facial width and body mass

Evolutionary studies suggest that facial width-to-height ratio (fWHR) is an important marker of fighting ability, dominance^52^, and perceived aggressiveness^53^. Following one such study^54^, we measured bizygomatic facial width and facial height in the facial portraits as a distance from the border of the upper lip to the glabella. We did this in the ImageJ programme using the mouse cursor and the ‘straight’ tool. All measurements were taken twice and consistency across measurements was verified by Intraclass correlations. Two measurements of the same feature in the same sample were accepted if they correlated > 0.9; then they were averaged across the same person. Facial width was scaled by facial height to obtain the facial width-to-height ratio of every stimulus person.

We measured and weighed the participants during the stimuli acquisition procedure using calibrated tools. The body mass index (BMI) was calculated as weight [kilograms] divided by the square of body height [metres].

#### Skin lightness and colour

We used the CIELab L*a*b* colour space, a tool developed to yield a device-independent measurement of lightness and colour intensity change as perceived by the human eye (by a ‘standard observer’)^55^. This space consists of three dimensions: L* (scale from black to white), a* (from green to red), and b* (from blue to yellow), all of which we used in the study.

In the Cameroonian sample, we measured the CIELab dimensions from facial photographs using the ImageJ programme^56^ with the ‘lab’ setting within the Color Transformer 2.02 plugin. In the Czech 2016 and 2019 sample, we took analogical measurements from faces in vivo using a spectrophotometer (Ocean Optics Flame-S, 200–850 nm, with optical resolution 2 nm). We took three measurements (cheeks and mid-forehead) and recorded the respective L*a*b* values. In previous research, similar results were obtained when using measurements taken in vivo with a spectrophotometer and using skin colouration measurements taken from facial photos^57^. We did not, however, combine these measurement approaches in a single set.

#### Geometric morphometrics

We employed geometric morphometrics to calculate the level of SShD and distinctiveness of all facial configurations within a set. We also calculated ‘shape dominance’ and ‘shape sex-typicality’ based on analyses of geometric morphometrics of facial configurations. Landmark-based geometric morphometrics is an approach that quantifies biological shapes and describes, compares, and visualises their variability^58^.

We landmarked all facial photographs using a stable set of 72 landmarks as defined in^59^. In total, 36 were true landmarks and 36 were a posteriori indicated as semi-landmarks. Landmarks are anatomically or geometrically homologous points that delimit the analysed object. Semi-landmarks denote curves and outlines within a structure between the true landmarks^60^. Landmarks were applied manually in the tpsDig2 software, ver. 2.31^61^. We ran Procrustes superimposition of all landmark configurations within each set using the gpagen() function from the R package Geomorph^62^. In this analysis, semi-landmark positions are computationally optimised by sliding along a tangent of the curve denoted by these semi-landmarks. The process results in minimising Procrustes distances between the corresponding points in different faces (or generally, landmark configurations) within a dataset. Outcomes of such superimposition may be further processed to obtain the relative position of a single facial configuration within the set.

Accordingly, we computed morphological distinctiveness (DIST) of a face as the Procrustes distance of individual facial configuration from the sample mean. The higher the numeric value a given face gets, the more distinctive (i.e. less average) the face is with regard to other faces in the set.

We also computed facial sexual shape dimorphism (SShD). This measure was used to measure relative facial sexual dimorphism of each individual facial configuration within a set. It is determined by projecting individual facial configurations from the high-dimensional morphological space of faces onto a vector that connects male and female means (mean configurations) within a sample^45^. Lower SShD values indicate more female-like facial shapes, while higher values indicate more male-like facial shapes.

Shape dominance and shape sex-typicality (i.e. shape masculinity of men, shape femininity of women) were obtained as per-face coefficients from the regression of facial configurations onto the perceived characteristics that was fitted using the procD.lm() function from the geomorph package^62^. The regressions were done separately for perceived dominance and perceived sex-typicality as independent variables. As such, the two variables explain the portion of the variance in rating that can be ascribed to variance in facial shape.

### Data analyses

All analyses were conducted within the R software, ver. 4.0.3^63^. First, we assessed interrater reliability of sex-typicality and dominance ratings using an intraclass correlation coefficient, which we calculated using the ICC() function of the R package ‘psych’^64^. Given that all raters rated all stimuli within a set of a stimuli of a given sex, we applied a two-way, average score interrater consistency analysis^65^.

We were interested in the predictions of perceived traits for each face, not in the ratings of individual raters, which is why we averaged the ratings across raters.

We used Bayesian inference to evaluate joint posterior distributions of plausible combinations of parameter values in a mediation analysis rooted in multiple linear regression. Age, BMI, CIELab L*, a*, b*, fWHR, SShD, and distinctiveness of facial shape (DIST) served as predictors of two correlated dependent variables: perceived sex-typicality (masculinity of men, femininity of women) and perceived dominance. We developed a directed causal model (containing only continuous linear predictors and continuous dependent variables) as follows: Age was independent of the other variables, BMI was predicted only by age, and age and BMI predicted all other variables. CIELab L*, a*, b*, fWHR, SShD, and DIST were predicted by age and BMI in a single multivariate distribution of mediators (covariances between them were included in the model). These mediators predicted intercorrelated dimensions of perceived dominance and sex-typicality (i.e., perceived masculinity of men, perceived femininity of women). The perceived characteristics were the main outcome variables. We did not investigate a directed association between perceived dominance and perceived sex-typicality, which is why we report their residual covariance. Before the analyses, all variables were standardised within samples.

In an alternative analysis, we also fitted shape dominance and shape sex-typicality as predictors of perceived sex-typicality and dominance. Shape dominance and sex-typicality were predicted by age and BMI and entered into a multivariate distribution of mediators (with CIELab L*, a*, b*, fWHR, BMI, SShD, and DIST on the same level in the multiple regression layout, see Fig. 1). To make sure that none of the reported effects are caused by the inclusion of intercorrelated predictors, we fitted also models that go only half-way towards the full model (see the concluding paragraphs of the Introduction above). In these, we used either shape dominance or shape sex-typicality (shape masculinity of men, shape femininity of women). We report these analyses in the online Supplementary Material, Figs. S1 and S5. The layout of the fitted models is in the upper part of Fig. 1 in this article.

The Bayesian models were fitted using the ulam() function of the rethinking package^66^ using an implemented Markov chain Monte Carlo Stan infrastructure^67^. The ulam() function converted the model layout into Stan syntax and sampled the posterior probability distribution to assess the joint distribution of likely parameter values. We extracted 10,000 samples from each joint posterior distribution (separately for each fitted model).

The sampled parameter values were: nine intercepts-one for each variable except for age-and twenty-nine slopes. There was one slope for each unidirectional relationship (eight slopes: Age, BMI, L*, a*, b*, fWHR, SShD, and DIST for each outcome variable, meaning perceived sex-typicality and dominance), two slopes regressing each mediator on BMI and age, and one slope from age to BMI. We employed multivariate distributions to evaluate residual correlations between mediators (6 variance, 15 covariance parameters) and between outcome variables (2 variance, 1 covariance parameter). Multivariate normal distributions were parametrised by vectors of two (predicted perceived sex-typicality, dominance), respectively six (predicted fWHR, DIST, SShD, L*, a*, b*) values coming from the linear regression of the abovementioned terms, residual correlation matrix between variables within each set, and vectors of variables’ standard deviations.

In the alternative analysis with shape dominance and shape sex-typicality (shape masculinity of men and shape femininity of women), the number of intercepts (11), slopes (37 in total, ten for each outcome variable, two slopes regressing each mediator on BMI and age, and one slope from age to BMI), and the number of variance (8) and covariance parameters (28) between mediators were changed accordingly; the same applies to the ‘shape dominance only’ and ‘shape sex-typicality only’ partial models. In all other respects, the model’s layout remained unchanged.

For each model parameter in all fitted models, we used unbiased weakly regularising priors. Priors for intercepts were characterised by a normal distribution with mean = 0 and SD = 0.2, priors for slopes by normal distribution with mean = 0 and SD = 0.5. The two correlation matrix priors (residual correlation within mediators and outcome variables) were defined using LKJ correlation matrix distribution with $\eta$=2, favouring correlations closer to 0 over extreme values. We characterised priors for standard deviations by exponential distributions with λ = 1.

Sampled posterior distribution of plausible parameter values was very wide along the margins of CIELab L*, a*, b*, and slope parameters characterising the effect of these colour dimensions, because in the Cameroonian sample, the outcomes were heavily correlated (see Supplementary Fig. S5 A, F), which suggested extreme collinearity. Aiming to reduce this collinearity, we ran a parallel factor analysis (using the fa.parallel() function within the ‘psych’ package^64^) and revealed a single underlying latent factor. We extracted this factor’s score and created a new variable called ‘colour’, using the fa() function of the ‘psych’ package. Thus updated Bayesian models were adequately reduced (the estimated parameters for L*, a*, b* were reduced to a single estimated parameter for ‘C’ and other model parameters were changed accordingly). Otherwise, the layout remained unchanged. To illustrate the predictions of all the fitted models, we drew density plots outlining the marginal distributions of likely parameter values. We used functions implemented within base R graphics to draw the mean and 95% credibility intervals for sampled parameter distribution (all model coefficients are in Table S3 and Fig. S5 in online supplementary material).

## Results

### Association between perceived variables

In men in general, we found a positive association between perceived dominance and perceived sex-typicality (masculinity). In Cameroonian men, the sampled posterior distribution of residual covariance between perceived dominance and perceived sex-typicality (perceived masculinity) had a mean of 0.42 (95% Compatibility Interval [CI]: 0.16, 0.64), while for sample of Czech men, the mean reached 0.77 (CI: 0.67, 0.85). In both the cultures, therefore, men who were perceived as more masculine were also perceived as more dominant, though the association was stronger in the Czech than in the Cameroonian men.

In Cameroonian women, the mean for the sampled posterior distribution of residual covariance between perceived femininity and dominance was weakly negative − 0.24 (CI: − 0.49, 0.03), suggesting that more dominant women were probably perceived as slightly less feminine. In the sample of Czech women, the estimated residual covariance between perceived femininity and dominance was moderately positive. The mean of sampled posterior distribution was 0.31 (CI: 0.13, 0.47), which indicates that more feminine Czech women (perceived femininity) were perceived as more dominant.

### Perceived variables vs. measured sexual shape dimorphism (SShD) and distinctiveness (DIST)

In landmark-based morphometric analysis, faces with more male-like shape yielded relatively higher SShD coefficients and faces with more female-like shape relatively lower SShD coefficients. This is important for a proper understanding of the bivariate coefficients reported below.

In Cameroonian men, perceived masculinity (mean slope: 0.29, CI: 0.06, 0.52) and dominance (mean slope: 0.29, CI: 0.01, 0.57) were positively associated with SShD, indicating that more male-like facial configurations were perceived as more masculine and dominant. In the sample of Czech women, the slope between perceived femininity and SShD was with a high level of likelihood negative with a slope of − 0.24 (CI: − 0.44, − 0.04). This suggests that faces with more female-like facial shape were perceived as more feminine. In Cameroonian women, the mean slope of partial correlation between perceived dominance and SShD was with a high level of likelihood positive (more female-like facial configurations being perceived less dominant; mean slope: 0.24, CI: − 0.04, 0.52).

In Czech women, lower distinctiveness of facial configurations was associated with higher perceived femininity − 0.26 (CI: − 0.44, − 0.08). Facial configurations that were less average with respect to typical facial configuration in the Czech female sample were thus perceived as less feminine. In the rest of the samples, we found neither conclusively positive nor negative association between perceived traits and distinctiveness (see Fig. 1).

### Perceived variables vs. measured skin colour

Using factor analysis, we computed the continuous variable ‘colour’, which most parsimoniously explains the three closely correlated L*, a*, b* CIELab channels in the Cameroonian samples. The slope between perceived masculinity and colour (higher scores along all three CIELab dimensions, meaning basically lighter skin that allows both redness and yellowness to stand out) of Cameroonian men was negative –0.29 (CI: − 0.52, − 0.05). In Cameroonian women, the slope between perceived femininity and colour was conclusively positive 0.52 (CI: 0.27, 0.76).

In the sample of Cameroonian men, colour explains a substantial amount of the variability of redness a* (R^2^ = 0.999), and yellowness b* (R^2^ = 0.97), and a moderate amount of variability in lightness L* (R^2^ = 0.55). More masculine men in the Cameroonian sample have therefore darker, less red, and less yellow skin colour.

In Cameroonian women, Colour explains well all of the component variables: Lightness L* (R^2^ = 0.77), redness a* (R^2^ = 0.90), and yellowness b* (R^2^ = 0.999). More feminine women thus have a lighter, yellower, and redder skin than less feminine women.

All in all, while darker, less red, and less yellow facial skin lowered the perceived masculinity in Cameroonian men, relatively lighter and brighter-coloured skin predicted higher femininity ratings in Cameroonian women (see Fig. S6, which presents a diagram that describes a build-up of the underlying factor ‘Colour’ and counterfactual plots on the association between colour and perceived sex-typicality in the Cameroonian sample).

In the Czech samples, the slope for association between colour channels and perceived sex-typicality and dominance was probably zero in every sampled posterior distribution. The only exception was that more masculine (mean slope: 0.18; CI: − 0.01, 0.37) and dominant (mean slope: 0.16; CI: − 0.04, 0.38) Czech men had a slightly yellower facial skin.

### Associations between perceived variables, age, and BMI

There was a positive slope between age and perceived masculinity in Cameroonian men, with a mean estimate of 0.31 (CI: 0.07, 0.55), indicating that older Cameroonian men were perceived as more masculine. In Czech men, age positively predicted both perceived masculinity (0.24; CI: 0.04, 0.42) and perceived dominance (0.23; CI: 0.02, 0.43). In Czech women, perceived dominance also increased with age (0.30, CI: 0.10, 0.50).

BMI affected perceived dominance in Cameroonian women, whereby relatively heavier women were likely perceived as more dominant (mean slope: 0.24; CI: − 0.04, 0.51). In Czech men, there was a moderately positive partial regression of BMI on perceived dominance (mean slope: 0.30; CI: 0.09, 0.50). Relatively heavier Czech men were therefore also probably perceived as more dominant.

### Effects of shape sex-typicality and shape dominance

Below, we report extended models which report on the effects of shape variance on perceived dominance and sex-typicality, thus adding to the default models listed above.

The overall magnitude of bivariate associations across the fitted multiple regressions was not substantially and/or systematically affected by entering shape dominance and sex-typicality into the models (see Fig. 1 in this article and Fig. S1 in online supplementary material). The exception were associations between the perceived characteristics and SShD, which became weaker, suggesting that variance explained by SShD may also be well explained by shape dominance and/or shape sex-typicality (see the Fig. 1 and S1). Additionally, credibility intervals for bivariate associations that included SShD or other shape-based variables widened, probably due to a collinearity between SShD and shape sex-typicality. In the sample of Czech women, this effect was the most pronounced. Residual covariance between SShD and shape femininity in Czech women had a mean of − 0.87 (95% CI: − 0.91, − 0.82). In fact, the previously negative association between SShD and perceived femininity (− 0.24) became in Czech women positive (mean slope = 0.42; CI: − 0.03; 0.87) once shape dominance and shape sex-typicality were entered into the analysis.

It seems therefore that in Czech women, collinearity between the two predictors (SShD and shape femininity) may be a concern. The rule of thumb in frequentist models is to cautiously approach predictors with VIF > 5. The VIF between SShD and shape femininity was 5.37, which indicates that this model structure would not work well with frequentist methods. On the other hand, Bayesian analysis in general prevents detrimental effects of collinearity better than standard frequentist methods do, because it penalises extreme regression coefficients proportionally to the standard deviation of parameters’ prior distributions. Based on a comparison between the default and extended model, we thus suppose that-unlike the SShD-shape femininity is a credible predictor of perceived femininity once both variables are entered into the same model. The same effect was also observed in a ‘partial’ model with only SShD and shape sex-typicality (shape femininity) without shape dominance.

Taken together, it is likely that shape dominance predicted perceived dominance and shape sex-typicality predicted perceived sex-typicality in all samples except for the association between perceived masculinity and shape masculinity in Cameroonian men. Moreover, due to a moderate collinearity between variables (all VIFs < 5), the seemingly positive association between shape dominance and perceived dominance in Cameroonian women may in fact be zero or even weakly negative (mean slope = 0.35; CI: -0.13; 0.80). Shape dominance and shape femininity were in this subsample positively correlated (mean of the partial correlation: 0.47; CI: 0.25, 0.66).

Table 2 summarises the most important results. The tables of mean slopes and credibility intervals for all fitted partial regressions and estimates for other model parameters (intercepts, standard deviations) are available in the supplementary materials (Table S3, Fig. S1 and S5). The supplementary materials also contain separate analyses on Czech 2016 and 2019 samples, which reveal similar patterns.

**Table 2:** Summary of selected bivariate associations.

Sample→	Cameroonian men			Czech men		
↓Association↓	Crediblity^a^	Par.	Interpretation	Crediblity^a^	Par.	Interpretation
Perc. masculinity	✓	0.42	Men perceived as more masculine were also perceived as more dominant	✓	0.77	Men perceived as more masculine were also perceived as more dominant
~~		95% CI:			95% CI:	
Perc. dominance		[0.16; 0.64]			[0.67; 0.85]	
Perc. masculinity	✓	0.29	More male-like facial configurations were perceived as more masculine	×?	0.15	Measured sexual shape dimorphism probably did not affect perceived masculinity
~		95% CI:			95% CI:	
SShD		[0.06; 0.52]			[− 0.04; 0.34]	
Perc. dominance	✓	0.29	More male-like facial configurations were perceived as more dominant	×	0.08	Measured sexual shape dimorphism did not affected perceived dominance
~		95% CI:			95% CI:	
SShD		[0.01; 0.57]			[− 0.13; 0.28]	
Perc. masculinity	✓	− 0.29	Darker, less bright-coloured men were perceived as more masculine	×?	yellowness [b*]: 0.18	Men with yellower facial skin tended to be perceived as more masculine; probably no other assoc.
~		95% CI:			[− 0.01; 0.37]	
Colour/L*,a*,b*		[− 0.52; − 0.05]				
Shape masculinity	✓	0.69	Shape component of perceived masculinity associated with male-like facial shape	✓	0.41	Shape component of perceived masculinity associated with male-like facial shape
~~		95% CI:			95% CI:	
SShD		[0.53; 0.81]			[0.24; 0.57]	
Shape masculinity	✓	0.58	Shape components of the perceived scales were positively related	✓	0.58	Shape components of the perceived scales were positively related
~~		95% CI:			95% CI:	
Shape dominance		[0.39; 0.74]			[0.42; 0.70]	

Sample→	Cameroonian women			Czech women		
↓Association↓	Crediblity^a^	Par.	Interpretation	Crediblity^a^	Par.	Interpretation
Perc. femininity	✓?	− 0.24	Women perceived as more feminine tended to be perceived as less dominant	✓	0.31	Women perceived as more feminine were perceived as more dominant
~~		95% CI:			95% CI:	
Perc. dominance		[− 0.49; 0.03]			[0.13; 0.47]	
Perc. femininity	×	− 0.08	No association between perceived sex-typicality and sexual shape dimorphism	✓	− 0.24	More female-like facial configurations perceived as more feminine
~		95% CI:			95% CI:	
SShD		[− 0.33; 0.17]			[− 0.44; − 0.04]	
Perc. dominance	✓?	0.24	More female-like facial configurations tended to be perceived as less dominant	×	− 0.02	No association between sexual shape dimorphism and perceived dominance
~		95% CI:			95% CI:	
SShD		[− 0.04; 0.52]			[− 0.22; 0.18]	
Perc. femininity	✓	0.52	Lighter and brighter-coloured women perceived as more feminine	×	L*: 0.01	No association between the three colour channels and perceived femininity
~		95% CI:			a*: 0.10	
Colour/L*,a*,b*		[0.27; 0.76]			b*: − 0.02	
Shape femininity	✓	0.34	The shape component of perc. femininity associated with less female-like SShD	✓	−0.87	The shape component of perc. femininity associated with more female-like SShD
~~		95% CI:			95% CI:	
SShD		[0.09; 0.55			[− 0.91; − 0.82]	
Shape femininity	✓	0.47	The shape components of perceived femininity and dominance positively related	×?	0.14	The shape components of perceived femininity and dominance were probably unrelated
~~		95% CI:			95% CI:	
Shape dominance		[0.25; 0.66]			[− 0.03; 0.31]	

^a^Once the 95% credibility interval of posterior probability density distribution of bivariate coefficients did not contain zero, we interpreted it as *credible* (we assume that the coefficient is in fact non-zero) and marked by ‘✓’. Borderline coefficients (CI containing zero but majority of the mass of the distribution above/below zero are marked with ‘✓?’. Credibility does not correspond to the direction of association (cf. Discussion where the results are being addressed with regard to hypotheses); Par. = Parameter value.

1st to 4th coefficients for each of the sample’s regression coefficients based on ‘default’ models without shape dominance and shape sex-typicality, 5th and 6th coefficients are residual covariances based on models with both shape dominance and shape sex-typicality. Relationships marked with ~~ are modelled as correlational; ~ indicates regression slopes.

## Discussion

Traditionally, it has been assumed that perceived dominance is closely related to sex-typicality, whereby masculine traits are perceived as dominant and feminine traits as submissive. Recently, though, Hester and colleagues rejected the single bipolar sex-typicality scale underlying the perception of sex-typical characteristics. They suggest that masculinity and femininity are two concepts that are negatively related but not mutually opposite^43^, which is why the association between dominance and sex-typicality may differ between the scales and between the sexes.

Moreover, some methods, especially the artificial masculinisation/feminisation of facial stimuli, may fail to capture the effect of facial sex-typicality on perceived characteristics accurately^41,68^. For these reasons, some researchers prefer working with natural facial stimuli which are optimally standardised but unaltered by any manipulation technique^46,69–71^. It also seems more appropriate to simultaneously use several facial features as predictors of a perceived characteristic. Moreover, when collected in distant populations, facial ratings may shed light on population-specific effects of variability of sex-typical facial shape and skin colour on perceived characteristics^48^.

Our study used unmanipulated standardised facial stimuli from two distant populations, one European (WEIRD) and one African (non-WEIRD), to explore the association between sex-typical traits and perceived dominance. Our results can be most parsimoniously interpreted as providing indirect support for the two-scale approach to human sex-typicality (as opposed to the concept of masculinity–femininity as a continuous bipolar dimension or two strictly parallel dimensions).

SShD and skin colour did not consistently predict perceived sex-typicality and dominance. Moreover, while the perceived masculinity of men was positively associated with perceived dominance, perceived women’s femininity was only weakly and inconsistently associated with perceived dominance. Shape sex-typicality and shape dominance altered the effect of SShD, but not the effect of skin colour and perceived sex-typicality. Finally, our assumption of differences across populations has been substantiated: skin colour affected the perceived traits more in the Cameroonian sample.

All in all, we uncovered no evidence of universality of a continuous linear scale from masculinity into femininity. Our results rather suggest that both higher masculinity and higher femininity may be in some samples related to higher perceived dominance. The same is obviously true for SShD (shape sex-typicality) and colouration. Although these scales can be treated as bipolar (from male to female extreme), they do not neatly and consistently align and predict sex-typicality and dominance neither across the populations nor across the sexes.

Men who were perceived as dominant were also perceived as masculine. We can thus assume that these two psychological scales are based on similar facial traits and perceived male sex-typicality (masculinity) affects perceived dominance. This association is in agreement with previous evidence^11–13^. On the other hand, while perceived masculinity and dominance were strongly related in the Czech sample (r = 0.77), in the Cameroonian sample their association was only moderately strong (r = 0.42). Given that Cameroon can be regarded a non-WEIRD country, we reviewed other recent studies on the association between sex-typical traits and dominance in non-WEIRD populations. U.S. and Mexican (non-WEIRD) raters agreed on the perception of facial dominance^72^, but that study did not compare the effect of sex-typicality on dominance perception across the cultures. A comparison between Europeans (United Kingdom) and the Japanese revealed that facial masculinisation increased attributions of dominance and masculinity in both samples^73^. In a Turkish male sample, perceived dominance was closely related to perceived masculinity (r = 0.87; see^74^). In an Arab sample from multiple countries, female raters perceived masculinised male but not masculinised female faces derived from^74^ as more dominant^75^, thus supporting our conclusion that the association between sex-typical features and dominance perception differs across sexes.

A recent study has revealed that across distant populations, dominance is one of key dimensions assessed from faces. That, however, only holds when the dimensions are forced to be uncorrelated, i.e. under the assumption of their orthogonality^76^. Moreover, another cross-cultural study^77^ did not identify dominance as such as an implicit dimension of facial impressions, despite finding considerable agreement across two distant (British and Chinese) participants’ cultures. The Czech vs. Cameroonian differences in dominance perception may thus partly result from limited cross-cultural universality of the impression of dominance.

Cross-cultural research had further shown that preference for sex-typical traits varies in connection with differences in local socioeconomic environment and its harshness^78,79^. It is therefore possible that local circumstances affect the association between dominance and sex-typical traits as well as the relative importance of perceived dominance.

Concerning women, our results between the population samples differ: Czech women with higher perceived femininity were actually perceived as more dominant, while in Cameroonian women the association between perceived femininity and dominance was weakly negative. Our results thus diverge from previous findings according to which facial feminisation lowers perceived dominance^73^, more feminine women are behaviourally less dominant^3^, and more female-like faces are perceived as less dominant in a mixed-sex sample of computer-generated faces^80^. There is, however, also evidence that corresponds to our results: in the aforementioned Turkish sample^74^, perceived dominance correlated positively with perceived attractiveness (r = 0.30) and femininity (r = 0.37). Such counterintuitive results could be due to the close resemblance between feminine and attractive female facial features: for instance in the aforementioned Turkish sample, perceived femininity was strongly associated with perceived attractiveness (r = 0.91; see^70,74^). The idea that feminine facial traits are regarded attractive in women is widely accepted^68,81,82^. Raters may thus perceive feminine women as attractive and due to the halo effect of attractiveness^83^ as also more competent, powerful, and ultimately also dominant.

It is also possible that Czech raters perceived more feminine women as more prestigious and did not distinguish between the dominance and prestige scales^84^. Aside from that, as suggested above, the association between femininity and dominance may be scale-dependent. Feminisation^73^ and femininity scale of computer-generated faces^80^ may capture different (presumably non-dominant) features than perceived femininity in unaltered real faces.

Shape dominance and shape sex-typicality predicted perceived characteristics as expected: shape dominance was positively associated with perceived dominance and shape sex-typicality was associated with perceived sex-typicality across the samples. Moreover, shape dominance and shape masculinity were positively associated across the two male samples. Surprisingly, though, we found a relatively strong positive association between shape dominance and shape femininity also in Cameroonian women. Although in that subsample perceived femininity itself correlated negatively with perceived dominance, the shape components of the two variables were in fact positively associated. This also suggests that sexually dimorphic perceived characteristics and sexually dimorphic shape variance scales do not orientate in the same way on any underlying universal sexually dimorphic scale.

In sum, when associated with perceived dominance in both sexes, neither perceived masculinity of men, femininity of women, nor the shape components of the perceived scales behave as two halves of a single scale. Other sexually dimorphic variables likewise did not follow the ‘unidimensional understanding’ of human sexually dimorphic scales.

Both Czech and Cameroonian raters are probably familiar with some variation in skin colour within their own populations. Despite this, only perceived sex-typicality in the Cameroonian samples was conclusively affected by skin colour as expressed by the ‘colour’ variable. Darker, less red, and less yellow skin was perceived more male sex-typical and less female sex-typical. This is consistent with previous studies which showed that skin colour, particularly its lightness, redness, and yellowness, is more important for the perception of facial characteristics in African populations^48,57^. Also, our results support the suggestion that lighter skin is a sex-typical trait associated with femininity^85^. These results, however, contradict the assumption that redder skin would be perceived as more masculine, aggressive, and, accordingly, more dominant^34^. In contrast to our study, Stephen et al.^34^ told their participants to manipulate redness to make faces more dominant-looking. This procedure may have allowed them to detect subtler colour differences. Moreover, while in both populations we collected stimuli and ratings within young, mostly student populations, it is possible that the Cameroonian sample was more ethnically diverse. Ethnic diversity may have thus affected skin colouration and its perception disproportionately more in Cameroonians, which would help explain our finding that skin colouration variance was more important in Cameroon than in Czechia.

Unlike perceived sex-typicality, dominance seems relatively unaffected by skin colour. Still, although we did not determine the direction of association between perceived dominance and perceived sex-typicality, it is likely that this association is, in fact, directed. We could speculate that skin colour predicts perceived sex-typicality and perceived sex-typicality in turn predicts perceived dominance, not vice versa.

The effects of the morphometric variables (SShD, Distinctiveness) were relatively weak and sample-specific, even when we focused only on models without shape dominance and shape sex-typicality. In these ‘default’ models, no collinearity and suppressor effect could distort bivariate associations between variables. Europeans have more sexually dimorphic facial morphologies than Africans do^45^ but in our study, SShD did not predict the perceived characteristics in the Czech (European) sample relatively more than in the Cameroonian (African) sample.

We added age into the analyses because facial cues of ageing strongly affect perceived facial characteristics^86^, including sex-typicality and dominance^13^. Older Cameroonian men in our sample were perceived as more masculine. Older Czech men were perceived as both more dominant and more masculine. In Czech women, higher age was also associated with higher dominance ratings. Although the means of sampled distribution of regression coefficients were weak to moderate, the positive effect of age on perceived sex-typicality and dominance was relatively stable across the samples.

### Limitations

On the basis of knowledge of a positive correlation between male masculinity and dominance, one cannot predict which association one ought to expect between female femininity and dominance. This statement seems true, but mainstream evolutionary psychology assumes the opposite. Some of the papers cited above assume that masculinity-femininity is a single unidimensional scale and some even tend to treat masculinity and femininity as complementary scales^3^. In our study, we showed that masculinity and femininity are probably two relatively unrelated scales, but we did not collect ratings of male femininity and female masculinity. The perception of sex-typicality is affected by knowledge of the gender of stimulus^44^. Rating of masculinity in women and femininity in men may thus be counterintuitive and prone to bias, cf^74^. The concept of sex-typicality is linked to gender stereotyping^44^. Anticipated gender identity of an adult stimulus is important for characteristics ascribed to such stimulus: this has conclusively shown by studies on the stereotyping of transgender persons^87^. Although it might be possible to obtain femininity ratings of male stimuli and masculinity ratings of female stimuli, these ratings would quite possibly be biased by stereotypes associated with the gender ascribed to the stimuli.

Still, future studies could attempt to collect such ‘full-scale’ ratings (femininity and masculinity of both sexes) but we would recommend that in doing so, they should present the faces of men and women in a randomised order. Additionally, they also should omit any specification of the sex of stimuli faces to prevent sex information from affecting the results. A future study could also collect ratings of both sex-typicality and sex-atypicality (as two scales). That would minimise the risk of gendered ratings but preserve the approach of collecting ratings on two theoretically associated sex-typicality scales.

Other potential limitation of our study stems from certain methodological discrepancies between the datasets. While in Cameroon, we collected only facial photographs and did not measure skin colouration with spectrophotometer in vivo, in the Czech samples, we did measure skin colouration by spectrophotometer in vivo. Nevertheless, there is some evidence to the effect that analyses based on in vivo measurements and skin colouration measured from facial photos yield similar results ^57^; see also^88^.

Another limitation stems from the different approach to rating collection in Czech and Cameroonian samples. While in the Czech collection of sex-typicality ratings, we used only opposite sex-ratings, in Cameroon, due to the limited number of potential raters, we decided to collect sex-typicality ratings from raters of both sexes with respect to stimuli of both sexes.

## Conclusions

We show that the pattern of association between sex-typical scales and perceived dominance differs across sexes and cultures. Also, within the same-population sample, sexually dimorphic scales do not follow a universal layout, showing that a unidimensional understanding of human sexual dimorphism is likely incorrect. Future studies should treat male and female sex-typicality as related but not mutually opposite scales. The association between female sex-typicality and dominance may in fact differ from a straightforward strong negative relationship, mainly with regard to its shape and non-shape (e.g. skin colour, texture) component. Our findings thus call for greater caution when interpreting results of studies that manipulate shape of facial stimuli.

## Supplementary Information

Supplementary Information.

## Data Availability

The dataset and R code is available at https://osf.io/mqgxa/?view_only=a42db3ea5d0f4bd3b76e4d614575ba92.
