# Supplementary material for: Africans and Europeans differ in their facial perception of dominance and sex-typicality: a multidimensional Bayesian approach
Source: Sci Rep. 2022 Apr 26;12:6821. doi: 10.1038/s41598-022-10646-6 (PMC9042949; doi:10.1038/s41598-022-10646-6)
Supplement: Supplementary file 1 — Supplementary Information. [file 41598_2022_10646_MOESM1_ESM.pdf]

Africans and Europeans differ in their facial perception of dominance and sex-typicality: A multidimensional Bayesian approach

Vojtěch Fiala[a,]*(iD: https://orcid.org/0000-0002-0148-5092), Petr Tureček[a,b](iD: https://orcid.org/0000-0003-2118-9909), Robert Mbe Akoko[c](iD: https://orcid.org/0000-0003-2804-4219), Šimon Pokorný[a](iD: https://orcid.org/0000-0001-5204-1048), Karel Kleisner[a](iD: https://orcid.org/0000-0002-3277-8365)

[a] Department of Philosophy and History of Science, Faculty of Science, Charles University, Prague, Czech Republic

[b] Center for Theoretical Study, Charles University and Czech Academy of Sciences, Prague, Czech Republic

[c] Department of Communication and Development Studies, University of Bamenda, Cameroon

* Corresponding author: Department of Philosophy and History of Science, Faculty of Science, Charles University, Viničná 7, 128 44 Prague, Czech Republic.
E-mail address: fialavoj@natur.cuni.cz

Supplementary Material

S.1. Extended theoretical framework

S1.1. Attribution of personal characteristics based on facial cues

The evolutionary-psychological studies of human visual perception and attribution of personal characteristics assume that certain facial features point to specific personal attributes. People do, in fact, associate other's visual facial traits stereotypically with various personal characteristics, e.g., perceived intelligence (Kleisner et al., 2014), propensity to aggression (Třebický et al., 2013), or un/trustworthiness (Linke et al., 2016; Stirrat & Perrett, 2010). Moreover, such attribution is rapid and spontaneous (Oosterhof & Todorov, 2008; Willis & Todorov, 2006). Using facial traits of newly acquainted individuals, people automatically assess their attributes (Todorov et al., 2009). Evolutionary psychologists have been explaining this phenomenon as an adaptation (Zebrowitz, 2006).

From an evolutionary perspective, individuals who could use facial cues to predict actual personal attributes were favoured over those who could not use facial features properly. Based on their assessment, human perceivers may predict the outcome of the subsequent interpersonal interaction and adjust their behaviour to maximise their benefits. In line with this prediction, research has shown that people assess some attributes, like upper-body physical strength of men (Sell et al., 2009), body mass (de Jager et al., 2018), or acute sickness (Axelsson et al., 2018) accurately according to facial features. However, in some characteristics, e.g., long-term health assessment (Kalick et al.,

1998; Rhodes et al., 2001), such accuracy has not been shown. Concerning psychological traits like trustworthiness, intelligence and agreeableness, there is evidence that the attribution is affected by an individual's concept of association among those psychological scales (Stolier et al., 2018). When a person believes that two psychological scales generally correlate in human personality (e.g., trustworthiness and intelligence), they tend to associate them with similar facial configurations. Therefore, the question of whether evolved cognitive architecture of humans serves to accurately perceive personal attributes remains open (see also Over & Cook, 2018).

S.1.2. Facial width to height ratio

To save space and keep the article's background section concise, we omitted a review of relevant literature on facial width-to-height ratio (fWHR). We, however, recognise that some readers could miss a detailed overview of the evolutionary research on human fWHR. fWHR, once assumed a sexually dimorphic (Carre & McCormick, 2008; Geniole et al., 2015) and testosterone-dependent measure (Lefevre et al., 2013), has been associated with perceived human aggressiveness (Lefevre & Lewis, 2014), perceived dominance (Dixson, 2017), actual fighting ability (Třebický et al., 2015), but also with cheating and explicit deception during negotiation (Haselhuhn & Wong, 2012), and with a propensity to rule violation and sports aggression (Carre & McCormick, 2008; Geniole et al., 2015). In subsequent research, some of the suggested associations were challenged. fWHR may not be sexually dimorphic in some populations (Kramer, 2015; Özener, 2012). It may not present a reliable proxy to sex hormones levels (Bird et al., 2016; Kordsmeyer et al., 2019), neither predict anti-social and risky behaviour (Wang et al., 2019). Moreover, variance in perceived aggressiveness is associated with a complex change of facial features rather than being predicted solely by fWHR (Třebický et al., 2013). When measured from facial photos, fWHR also change with alteration of focal length (Třebický et al., 2016).

Taken together, the association between fWHR and perceived traits is not stable across samples and populations and depends on study methodology. Therefore, we primarily attempted to express human sexual dimorphism and distinctiveness/typicality of facial configuration using more complex landmark-based methods of geometric morphometrics. These methods may better capture the overall change of facial shape with perceived characteristics. Nonetheless, we entered fWHR (measured in a standardised way, see Třebický et al., 2016) into the analyses as widely used facial ratio.

S.2. Methods: Detailed overview

S.2.1. Stimuli acquisition and processing

Participants for stimuli acquisitions were recruited by direct personal invitation, using social networks and fliers located around the place of stimuli collection.

We collected the Cameroonian photographic stimuli in 2013 at the University of Buea, located in the Town of Buea (local centre, population ~300 000), in the Southwest Region of Cameroon. The stimuli in Cameroon were photographed with a DSR camera, Nikon D90. We set the exposure time to 1/100, ISO to 100, focal length to 50.00 mm, and aperture to 8.0. The scene was illuminated by an electronic studio flash and an oval collapsible reflector. The camera shutter was released manually; the Camera was mounted on a tripod (Velbon Sherpa). Photos were saved in the resolution of 4352×2868 (height × width) pixels as .raw files for subsequent processing and analyses. We used plain white background behind the photographed persons.

The 2016 Czech stimuli were photographed using a DSR camera Fujifilm Finepix S602 Zoom. Exposure time was 1/100; ISO was set to 200, the aperture was 5.0, and the focal length was 85 mm. The scene was illuminated with Photon Europe MSN HSS-800Ws with a reverse 120cm lighting umbrella. Images were stored as .raw files in the resolution (height×width) 2678 × 2674 pixels for

subsequent processing and analyses. The photographs were taken at the Faculty of Science, Charles University, Prague, in autumn 2016.

Czech photos from 2019 were taken with a DSR camera Canon EOS 6D. Exposure time was set to 1/100, ISO speed was 100, and the focal length was set to 85 mm. The scene was illuminated in the same way as in the 2016 Czech sample. We stored the images in a JPG file in resolution 5472×3648 pixels for subsequent image processing and analyses. The photographs were taken at the Faculty of Physical Education and Sport, Charles University, Prague, in spring 2019. In both the 2016 and 2019 collections, the participants were photographed in a photographic tent (a cube with a background, sidewalls, ceiling, and floor covered with plain white fabric).

Because the two Czech samples were similar concerning the process of acquisition, age of the participants, and applied measurements, we combined them into a single sample of stimuli. Nonetheless, in online Supplementary Material, we also report Bayesian analyses conducted on the separated samples, given that those samples were collected and rated separately (see Figure S5, section S to AH). Figure S3 in online supplementary material (see below and in the osf storage; https://osf.io/mqgxa/?view_only=a42db3ea5d0f4bd3b76e4d614575ba92) presents an overview of relative distribution densities of variables within the Czech samples. Table S1 presents an overview of descriptive statistics per variable and sample.

Table S1. The counts of stimuli and their descriptive statistics

sample	n	Variable	mean	SD	min.	max.	skewness	kurtosis
CMR women	50	Age	21.2400	1.8905	17.0000	25.0000	-0.2332	2.9363
CZ women	106		23.0943	4.1185	19.0000	39.0000	1.4865	5.1853
CMR women	50	BMI	24.2958	4.1674	18.5918	35.8177	0.9145	3.3457
CZ women	106		21.8002	2.8882	17.3380	38.1039	2.2374	12.4007
CMR women	50	fWHR	2.1564	0.1733	1.8542	2.7475	0.8596	4.5790
CZ women	106		1.9302	0.1232	1.6022	2.2786	0.0549	3.2297
CMR women	50	SShD	-0.0117	0.0132	-0.0400	0.0152	-0.1176	2.2112
CZ women	106		-0.0162	0.0169	-0.0663	0.0299	0.0682	3.2361
CMR women	50	DIST	0.0532	0.0117	0.0342	0.0811	0.4121	2.6173
CZ women	106		0.0574	0.0137	0.0293	0.1076	0.5524	3.5145
CMR women	50	L*	38.3559	6.4451	27.1070	55.4960	0.2804	2.6411
CZ women	106		61.3841	3.4570	53.8990	82.1737	1.7930	14.0212
CMR women	50	a*	21.0855	3.1804	13.1400	25.5980	-0.6660	2.7534
CZ women	106		17.3866	2.5013	11.5770	25.6563	0.8596	4.5338
CMR women	50	b*	18.1284	4.4549	8.2320	26.9420	-0.2899	2.4497
CZ women	106		13.1199	2.1353	8.5813	20.8030	0.8877	4.7524
CMR women	50	Perceived Femininity	4.8805	0.5776	3.5974	5.7792	-0.3888	2.3855
CZ women	106		3.9995	0.8430	1.6364	5.8000	-0.5412	3.0181
CMR women	50	Perceived Dominance	3.9030	0.4943	2.6729	4.9057	-0.2704	2.9256
CZ women	106		3.8128	0.6637	2.0500	5.3167	0.1177	2.7827
CMR women	50	Shape Dominance	0.0000	0.0005	-0.0011	0.0009	-0.0628	2.5243
CZ women	106		0.0000	0.0002	-0.0005	0.0004	-0.2751	3.2180
CMR women	50	Shape Femininity	0.0000	0.0001	-0.0003	0.0002	-0.3345	2.4282
CZ women	106		0.0000	0.0001	-0.0004	0.0004	-0.0510	3.9758
CZ women 16	50	Age	23.6400	4.3319	19.0000	36.0000	1.1690	3.7218
CZ women 19	56		22.6071	3.8926	19.0000	39.0000	1.8068	7.3106
CZ women 16	50	BMI	22.1580	2.9007	17.3380	31.8041	0.8121	4.2808

CZ women 19	56		21.4808	2.8650	17.5064	38.1039	3.5781	21.7611
CZ women 16	50	fWHR	1.9453	0.1336	1.6027	2.2381	-0.1778	2.7460
CZ women 19	56		1.9166	0.1126	1.6022	2.2786	0.2576	4.1226
CZ women 16	50	SShD	-0.0189	0.0185	-0.0715	0.0245	-0.1533	3.4288
CZ women 19	56		-0.0148	0.0158	-0.0580	0.0178	-0.1415	2.8358
CZ women 16	50	DIST	0.0542	0.0128	0.0336	0.0943	0.7470	3.5162
CZ women 19	56		0.0539	0.0131	0.0329	0.0827	0.5338	2.4194
CZ women 16	50	L*	60.2618	4.2697	53.8990	82.1737	2.5455	15.0421
CZ women 19	56		62.3861	2.0994	57.3893	68.2360	0.4618	3.2487
CZ women 16	50	a*	17.6742	3.2506	11.5770	25.6563	0.5309	2.9535
CZ women 19	56		17.1299	1.5454	14.1783	22.4950	0.8148	4.6700
CZ women 16	50	b*	13.6975	2.3369	10.3047	20.8030	1.0977	4.4823
CZ women 19	56		12.6042	1.8069	8.5813	16.9440	0.0840	2.5413
CZ women 16	50	Perceived Femininity	3.8550	0.8563	1.6364	5.1591	-0.4266	2.6692
CZ women 19	56		4.1286	0.8170	1.7500	5.8000	-0.6410	3.4807
CZ women 16	50	Perceived Dominance	3.7384	0.5745	2.4410	4.9876	0.0043	2.7393
CZ women 19	56		3.8792	0.7331	2.0500	5.3167	0.0603	2.5748
CZ women 16	50	Shape Dominance	0.0000	0.0003	-0.0006	0.0008	-0.0132	3.0734
CZ women 19	56		0.0000	0.0003	-0.0007	0.0008	-0.2691	3.8429
CZ women 16	50	Shape Femininity	0.0000	0.0001	-0.0003	0.0003	0.0159	3.2026
CZ women 19	56		0.0000	0.0003	-0.0008	0.0009	0.1271	3.9964
CMR men	49	Age	22.0000	2.2361	17.0000	30.0000	0.5586	4.8694
CZ men	89		23.3820	4.2493	19.0000	43.0000	1.7443	7.1638
CMR men	49	BMI	23.1507	2.3297	17.0079	30.9289	0.7794	4.9654
CZ men	89		22.9890	2.3634	16.2727	28.5968	-0.0831	3.1045
CMR men	49	fWHR	2.0925	0.1702	1.7672	2.4470	0.3519	2.4261
CZ men	89		1.8809	0.1131	1.6136	2.2692	0.5947	4.4770
CMR men	49	SShD	0.0119	0.0142	-0.0266	0.0380	-0.5041	3.1955
CZ men	89		0.0193	0.0147	-0.0281	0.0545	-0.0827	3.3766
CMR men	49	DIST	0.0535	0.0113	0.0344	0.0816	0.4107	2.7785
CZ men	89		0.0550	0.0124	0.0321	0.0889	0.7268	3.2129
CMR men	49	L*	32.9066	6.2404	20.5130	49.0840	0.3640	2.7752
CZ men	89		58.7741	2.7392	52.6407	67.9527	0.3877	3.6542
CMR men	49	a*	17.4403	3.4057	10.8470	25.0030	0.3775	2.6366
CZ men	89		19.1447	2.6620	10.7583	26.5867	-0.3132	3.4190
CMR men	49	b*	13.3267	4.3254	5.8560	24.9590	0.7545	3.3686
CZ men	89		13.7170	1.5656	10.1403	17.7813	0.3923	2.8779
CMR men	49	Perceived Masculinity	5.6157	0.5732	3.7792	6.4805	-1.0259	4.3047
CZ men	89		4.1701	0.8544	2.1905	6.3896	0.0941	2.8440
CMR men	49	Perceived Dominance	4.1097	0.5074	3.1121	5.4722	0.5546	3.3882
CZ men	89		3.9406	0.7093	1.9677	5.9548	0.2327	3.4539
CMR men	49	Shape Dominance	0.0000	0.0002	-0.0007	0.0004	-0.5702	3.4583
CZ men	89		0.0000	0.0001	-0.0002	0.0002	-0.0616	2.6031
CMR men	49	Shape Masculinity	0.0000	0.0003	-0.0008	0.0005	-0.3632	2.8819
CZ men	89		0.0000	0.0001	-0.0003	0.0002	-0.0555	2.7620
CZ men 16	50	Age	24.0400	3.9173	19.0000	34.0000	0.7632	2.6133

CZ men 19	39		22.5385	4.5529	19.0000	43.0000	2.7540	12.3519
CZ men 16	50	BMI	22.3771	2.2726	16.2727	27.5543	-0.2693	3.1922
CZ men 19	39		23.7736	2.2696	19.4458	28.5968	0.1249	2.5043
CZ men 16	50	fWHR	1.8852	0.1252	1.6136	2.2692	0.6396	4.6122
CZ men 19	39		1.8754	0.0969	1.6982	2.1011	0.2808	2.5935
CZ men 16	50	SShD	0.0189	0.0157	-0.0094	0.0503	0.0074	2.1050
CZ men 19	39		0.0213	0.0125	-0.0187	0.0437	-0.8250	4.4272
CZ men 16	50	DIST	0.0544	0.0119	0.0351	0.0846	0.7309	3.1112
CZ men 19	39		0.0489	0.0121	0.0333	0.0846	1.0443	3.7967
CZ men 16	50	L*	58.1568	2.3165	52.6407	64.5213	0.1258	3.2976
CZ men 19	39		59.5656	3.0516	53.6217	67.9527	0.2438	3.3402
CZ men 16	50	a*	18.7285	2.6559	10.7583	23.1633	-0.6106	3.2884
CZ men 19	39		19.6783	2.6066	14.4553	26.5867	0.0899	3.0375
CZ men 16	50	b*	13.8041	1.6254	10.9800	17.7000	0.3647	2.2892
CZ men 19	39		13.6052	1.4988	10.1403	17.7813	0.3841	3.8532
CZ men 16	50	Perceived Masculinity	4.0711	0.7994	2.4069	6.3896	0.1320	3.3224
CZ men 19	39		4.2971	0.9149	2.1905	6.1746	-0.0332	2.4526
CZ men 16	50	Perceived Dominance	3.8488	0.6621	2.6102	5.9548	0.5211	3.4691
CZ men 19	39		4.0583	0.7580	1.9677	5.8710	-0.1171	3.6703
CZ men 16	50	Shape Dominance	0.0000	0.0002	-0.0005	0.0004	-0.1427	2.8461
CZ men 19	39		0.0000	0.0001	-0.0003	0.0002	-0.6546	3.9374
CZ men 16	50	Shape Masculinity	0.0000	0.0002	-0.0003	0.0003	0.2337	2.3937
CZ men 19	39		0.0000	0.0001	-0.0003	0.0002	-0.1907	2.1567

BMI = body mass index; fWHR = facial width-to-height ratio; SShD = sexual shape dimorphism; DIST = morphological distinctivness (distance from mean sample facial morphological configuration); L*, a* b* - CIELab colour channels (in turn lightnesss, redness, yellowness); SD = standard deviation; CZ = Czech Republic, CMR = Cameroon.

S.2.2. Stimuli processing and rating collection

For the rating sessions, facial photos were colour-checked and corrected in Adobe Photoshop Lightroom. The X-Rite ColourChecker white card (Passport Photo 2) was used as a reference for white balancing and exposure calibration in Adobe Photoshop Lightroom 4. We adjusted the position of a face in the image to locate eyes in the same horizontal position and leave a standard length of the neck visible. The photos were resized to a lower resolution (~500×700 px) for collections of ratings.

We collected the ratings separately for each of the four photo sets. The rating collection was mediated by the Qualtrics online survey software (www.qualtrics.com). The questionnaires were distributed by e-mail invitation to potential raters, flyers in the university buildings, and publicly sharing the survey link on an online social networking service. We also used direct personal invitation in the university buildings in both the countries. Participants (raters) were debriefed with the purpose of the study and after confirming a consent for participation, they answered demographic questions concerning their age, weight, height, and settlement size. Then, they were instructed to rate a set of stimuli on a seven-point rating scale anchored as 1 = not at all feminine/masculine/dominant, 7 = very much feminine/masculine/dominant. Male stimuli were rated on masculinity; female stimuli were rated on femininity. Collectively, we term these ratings as perceived sex-typicality and perceived dominance. The ratings were self-paced, and the order of stimuli was (pseudo)randomised. Dominance and sex-typicality ratings were collected in separate questionnaires. Data collection in Cameroon was

held in the English language (in the Southwest region of Cameroon, English is the official language), data collection in Czechia was held in the Czech language.

In Cameroonian questionnaires, we kept only the answers from raters who confirmed their Cameroonian origin (all the participants did, except for one rater from Nigeria). In Czech questionnaires, we kept raters of Czech nationality and raters who stated the Slovak nationality, given the cultural and language similarity between Czechs and Slovaks (e.g., Nábělková, 2007). Three such raters appeared in the ratings of dominance and two in the ratings of sex-typicality in the Czech 2019 set, seven in the ratings of dominance in the Czech 2016 dataset.

Unlike in other questionnaires in which men and women rated facial photos of both men and women, in the questionnaire on sex-typicality (masculinity of men, femininity of women) of Czech 2016 stimuli, photos of men were rated by women and photos of women were only rated by men. Table S2 presents an overview of the samples of raters. ICC 3,k stands for Inter-rater agreement assessment that was calculated as intraclass correlation coefficient (a two-way, average score interrater consistency analysis ICC(3,k)).

Table S2. Descriptive statistics of the samples of raters

Country (year)	Rated set	No. Raters (k)	Female raters	Rater's age (mean±SD)	Female rater's age (mean±SD)	Male rater's age (mean±SD)	Inter-rater agreement (ICC 3,k)
Cameroon (2013)	Dominance - women	108	54	23.766±3.942	23.704±3.720	23.830±4.191	0.93 (CI: 0.90-0.95)
Cameroon (2013)	Dominance - men	108	54	23.766±3.942	23.704±3.720	23.830±4.191	0.93 (CI: 0.90-0.95)
Cameroon (2013)	Femininity - women	77	38	24.000±4.123	23.105±3.733	24.872±4.342	0.91 (CI: 0.88-0.94)
Cameroon (2013)	Masculinity - men	77	38	24.000±4.123	23.105±3.733	24.872±4.342	0.95 (CI: 0.94-0.97)
Czech rep. (2016)	Femininity - women	44	NA	30.000±9.683	NA	30.000±9.683	0.97 (CI: 0.96-0.98)
Czech rep. (2016)	Masculinity - men	231	231	32.039±7.595	32.039±7.595	NA	0.99 (CI: 0.99-1.00)
Czech rep. (2016)	Dominance - women	161	117	30.199±9.766	29.308±9.810	32.568±9.347	0.97 (CI: 0.96-0.98)
Czech rep. (2016)	Dominance - men	177	133	29.429±9.551	28.534±9.406	32.136±9.581	0.98 (CI: 0.97-0.99)
Czech rep. (2019)	Dominance - women	60	47	35.017±10.292	34.702±10.716	36.154±8.877	0.95 (CI: 0.93-0.96)
Czech rep. (2019)	Dominance - men	62	48	34.968±10.085	34.646±10.554	36.071±8.535	0.95 (CI: 0.93-0.97)
Czech rep. (2019)	Femininity - women	60	48	34.300±9.206	34.646±10.086	32.917±4.166	0.97 (CI: 0.96-0.98)
Czech rep. (2019)	Masculinity - men	63	50	34.079±8.977	34.500±9.826	32.462±4.313	0.97 (CI: 0.96-0.98)

S.2.3. Skin colouration measurement

We used CIEL*a*b* colour space which was developed to yield a device-independent measurement of the lightness and colour intensity change as is perceived by the human eye (by a ‘standard observer’) (McLaren, 1976). This space consists of three dimensions: L* (scale from black to white), a* (from green to red), and b* (from blue to yellow), all of which we used in this study.

In the Cameroonian sample, we measured the CIELab dimensions from the facial photos with the ImageJ software (Schneider et al., 2012), using the “lab” setting within the plugin Color Transformer 2.02. Color Transformer converts the colour of the image into the CIELab colour space.

The measurements combined three spots on the photographed faces (left and right cheek and mid-forehead). Measured areas were selected using the "rectangle" selection tool. The L*a*b* was calculated as the grand mean of the hue and tone of the three skin patches.

In the Czech 2016 and 2019 samples, we took the analogical measurements from faces in vivo using a spectrophotometer (Ocean Optics Flame-S, 200-850 nm, with optical resolution 2 nm). We calibrated the spectrophotometer with white standard (WS-1 Diffuse Reflectance Standard, Ocean Optics) before measurement. Subsequently, we checked and updated the calibration between the participants (given that the spectrophotometer heated itself). We seated a participant on a rotating chair, cleaned their skin with a liquid make-up remover to wash down cosmetics and dirt. The same cleaning procedure was applied to the measuring tool (integrating sphere) after each measurement. We took three measurements (cheeks and mid-forehead) and saved the L*a*b* values with Ocean View, ver. 1.6.7.

Unless the way of skin colour measurements differs across samples, the study methodology may not affect the results concerning the effects of skin colouration: (i) Photos were taken using the standardised procedure and were colour-calibrated and colour-checked before the colour measurements and rating collection; (ii) Contemporary evidence (Coetzee et al., 2012, see also Fiala et al. 2021) shows that results obtained with skin colour measured in vivo from facial skin and results of analyses that use skin colour measured from facial photos are similar with regard to the effect of skin colouration variance on the other traits (variables); (iii) we did not analyse the data across cultures, neither combined the datasets with the different methodology of skin colour measurements.

2.3 Data analyses

All analyses were conducted within the R software, ver. 4.0.3 (R Core Team, 2021). First, we assessed interrater reliability of sex-typicality and dominance ratings using an intraclass correlation coefficient, which we calculated using the ICC() function of the R package 'psych' (Revelle, 2018). Given that all the raters rated all stimuli within a set, we applied a two-way, average score interrater consistency analysis (Shrout & Fleiss, 1979).

We were interested in the predictions of perceived traits for each face, not in the ratings of individual raters, which is why we averaged the ratings across raters.

We used Bayesian inference to evaluate joint posterior distributions of plausible combinations of parameter values in a mediation analysis rooted in multiple linear regression. We developed a directed causal model (containing only continuous linear predictors and continuous dependent variables) as follows: Age was independent of other variables, BMI was predicted only by age, and age and BMI predicted all other variables. CIELab L*, a*, b*, fWHR, SShD, and distinctiveness of facial shape (DIST) were predicted by age and BMI in a single multivariate distribution of mediators (covariances between them were included in the model). These mediators predicted intercorrelated dimensions of perceived dominance and sex-typicality (i.e., perceived masculinity of men, perceived femininity of women). The perceived characteristics were the main outcome variables. We did not investigate a directed association between perceived dominance and perceived sex-typicality, which is why we report their residual covariance. Before the analyses, all variables were standardised within samples.

In an alternative analysis, we also fitted shape dominance and shape sex-typicality as predictors of perceived sex-typicality and dominance. Shape dominance and sex-typicality were predicted by age and BMI and entered into a multivariate distribution of mediators (with CIELab L*, a*, b*, fWHR, BMI, SShD, and DIST on the same level in the multiple regression layout, see Figure 1).

The Bayesian models were fitted using the ulam() function of the rethinking package (McElreath, 2020) using an implemented Markov chain Monte Carlo Stan infrastructure (Stan

Development Team, 2020). The ulam() function converted the model layout into Stan syntax and sampled the posterior probability distribution to assess the joint distribution of likely parameter values. We extracted 10,000 samples from each joint posterior distribution (separately for each fitted model).

The sampled parameter values were: nine intercepts – one for each variable except for age – and twenty-nine slopes. There was one slope for each unidirectional relationship (eight slopes: Age, BMI, L*, a*, b*, fWHR, SShD, and DIST for each outcome variable, meaning perceived sex-typicality and dominance), two slopes regressing each mediator on BMI and age, and one slope from age to BMI. We employed multivariate distributions to evaluate residual correlations between mediators (6 variance, 15 covariance parameters) and between outcome variables (2 variance, 1 covariance parameter). Multivariate normal distributions were parametrised by vectors of two (predicted perceived sex-typicality, dominance), respectively six (predicted fWHR, DIST, SShD, L*, a*, b*) values coming from the linear regression of the abovementioned terms, residual correlation matrix between variables within each set, and vectors of variables' standard deviations.

In the alternative analysis with shape dominance and shape sex-typicality (i.e., shape masculinity of men and shape femininity of women), the number of intercepts (11), slopes (37 in total, ten for each outcome variable, two slopes regressing each mediator on BMI and age, and one slope from age to BMI), and the number of variance (8) and covariance parameters (28) between mediators were changed accordingly.

For each model parameter in all fitted models, we used unbiased weakly regularising priors. Priors for intercepts were characterised by a normal distribution with mean=0 and SD=0.2, priors for slopes by normal distribution with mean=0 and SD=0.5. The two correlation matrix priors (residual correlation within mediators and outcome variables) were defined using LKJ correlation matrix distribution with η=2, favouring correlations closer to 0 over extreme values. We characterised priors for standard deviations by exponential distributions with λ=1.

Sampled posterior distribution of plausible parameter values was very wide along the margins of CIELab L*, a*, b*, and slope parameters characterising the effect of these colour dimensions, because in the Cameroonian sample, the outcomes were heavily correlated (see Supplementary Figure S4 A, F), which suggested extreme collinearity. Aiming to reduce this collinearity, we ran a parallel factor analysis (using the fa.parallel() function within the 'psych' package (Revelle, 2018)) and revealed a single underlying latent factor. We extracted this factor's score and created a new variable called 'colour', using the fa() function of the 'psych' package. Thus updated Bayesian models were adequately reduced (the estimated parameters for L*, a*, b* were reduced to a single estimated parameter for 'C' and other model parameters were changed accordingly). Otherwise, the layout remained unchanged. To illustrate the predictions of all the fitted models, we drew density plots outlining the marginal distributions of likely parameter values. We used functions implemented within base R graphics to draw the mean and 95% credibility intervals for sampled parameter distribution (all model coefficients are in Table S3 and Figure S4 in online supplementary material at https://osf.io/mqgxa/?view_only=a42db3ea5d0f4bd3b76e4d614575ba92).

The rest of the study methodology is described in sufficient detail in the main text.

S.2.5. Supplementary figures

In this section, there are five out of the six supplementary figures (Figure S6 is also located at: https://osf.io/mqgxa/?view_only=a42db3ea5d0f4bd3b76e4d614575ba92). Note that <u>high-resolution version of each of the figures (under the same name)</u>, associated data, supplementary tables, and R

scripts are located elsewhere (https://osf.io/mqgxa/?view_only=a42db3ea5d0f4bd3b76e4d614575ba92).

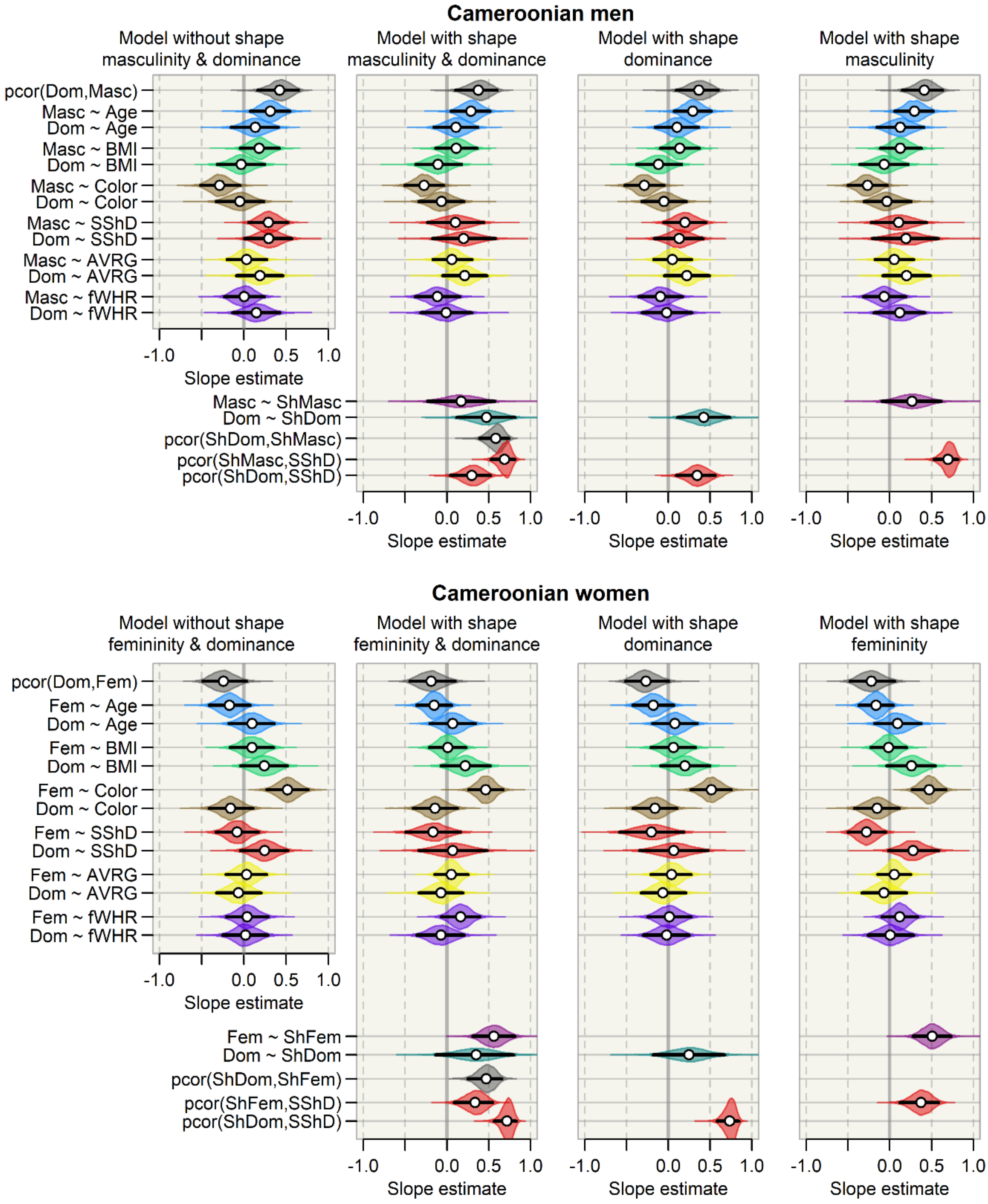

Figure S1a. Density plots of selected bivariate coefficients across models (based on Cameroonian data) (for a description, see Caption of Figure S1b below).

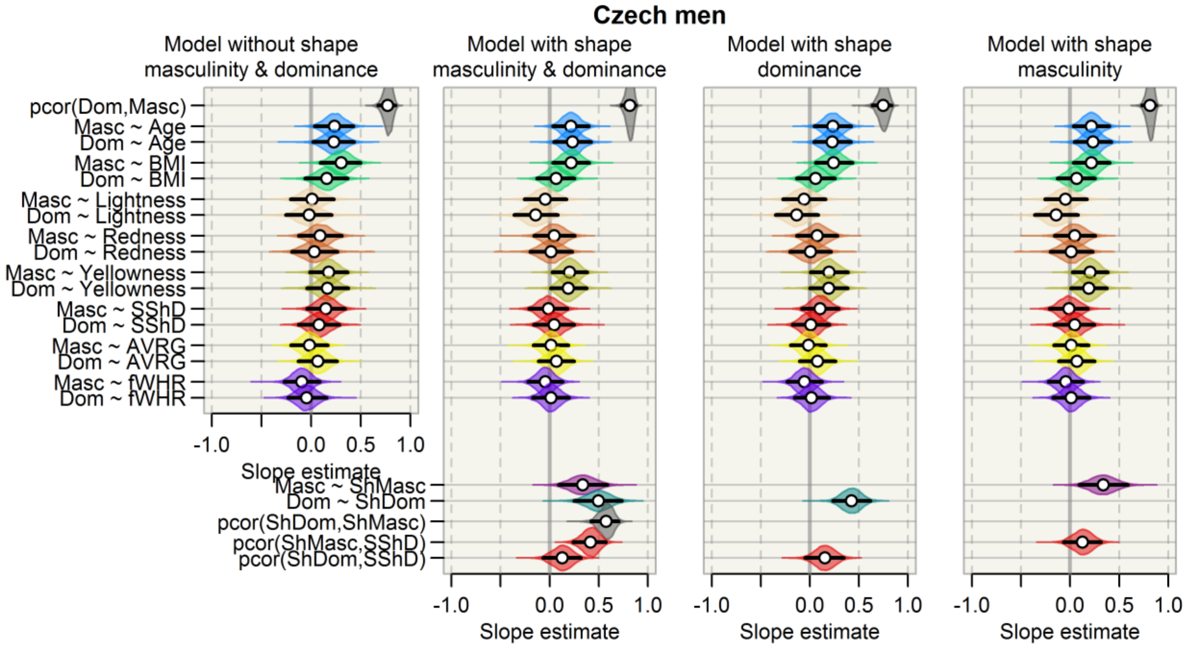

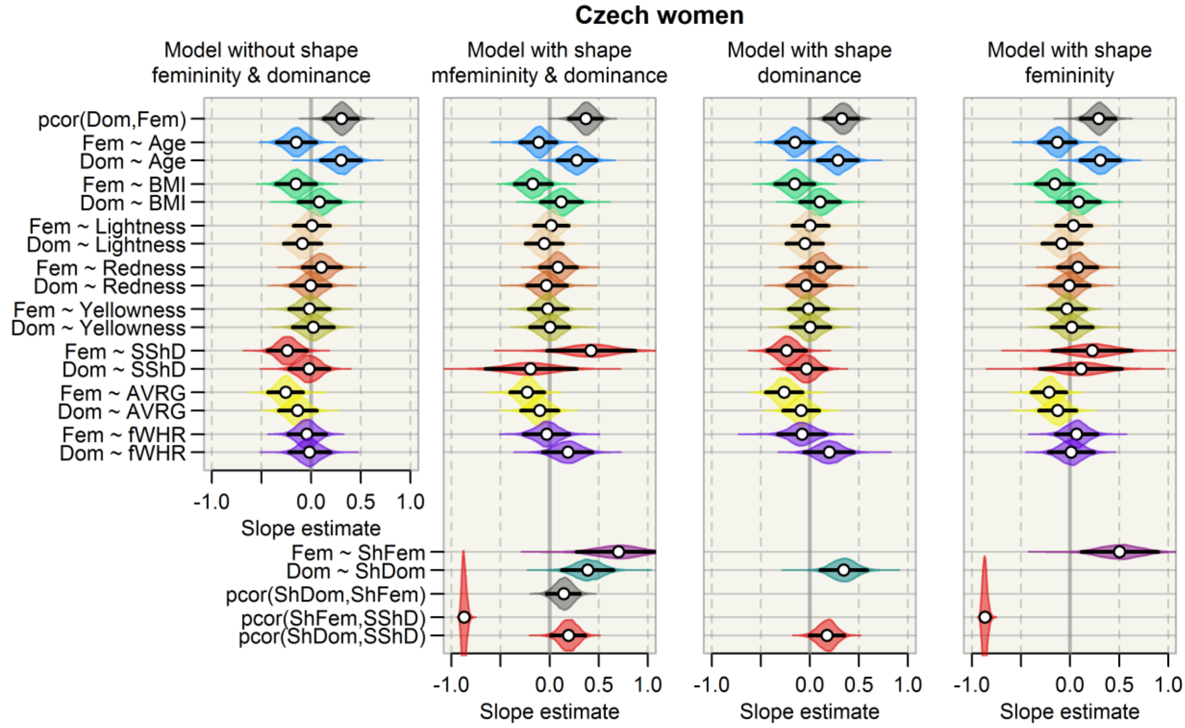

Figure S1b. Density plots of selected bivariate coefficients across models (based on Czech data). Model structure and density plots representing the posterior margins of selected coefficients (on a standardised scale) by sex, in the Cameroonian (**S1a**) and Czech sample (**S1b**). Sixteen panels of density plots represent posterior margins for a given sex, country (sample), and model layout. Density distribution correspond, from left to right, to these models: (1) 'Default model' – without shape variables derived from the perceived scales; (2) 'Full-scale model' – with 'Shape dominance' and 'Shape sex-typicality'; (3) 'Partial model #1" – with 'Shape dominance' only; (4) 'Partial model #2' – with 'Shape

sex-typicality' only. BMI = body mass index; fWHR = facial width to height ratio, SShD = sexual shape dimorphism, DIST = morphological distinctiveness; L*, a*, b* = lightness, redness, yellowness (CIELab L*a*b*); Masc/Fem = perceived sex-typicality (masculinity of men/femininity of women); Dom = perceived dominance; ShDom = shape dominance; ShMasc = shape masculinity; ShFem = shape femininity. Black error bars span the 95% compatibility intervals of the parameters. The complete posterior summary can be found in Supplementary Figure S5.

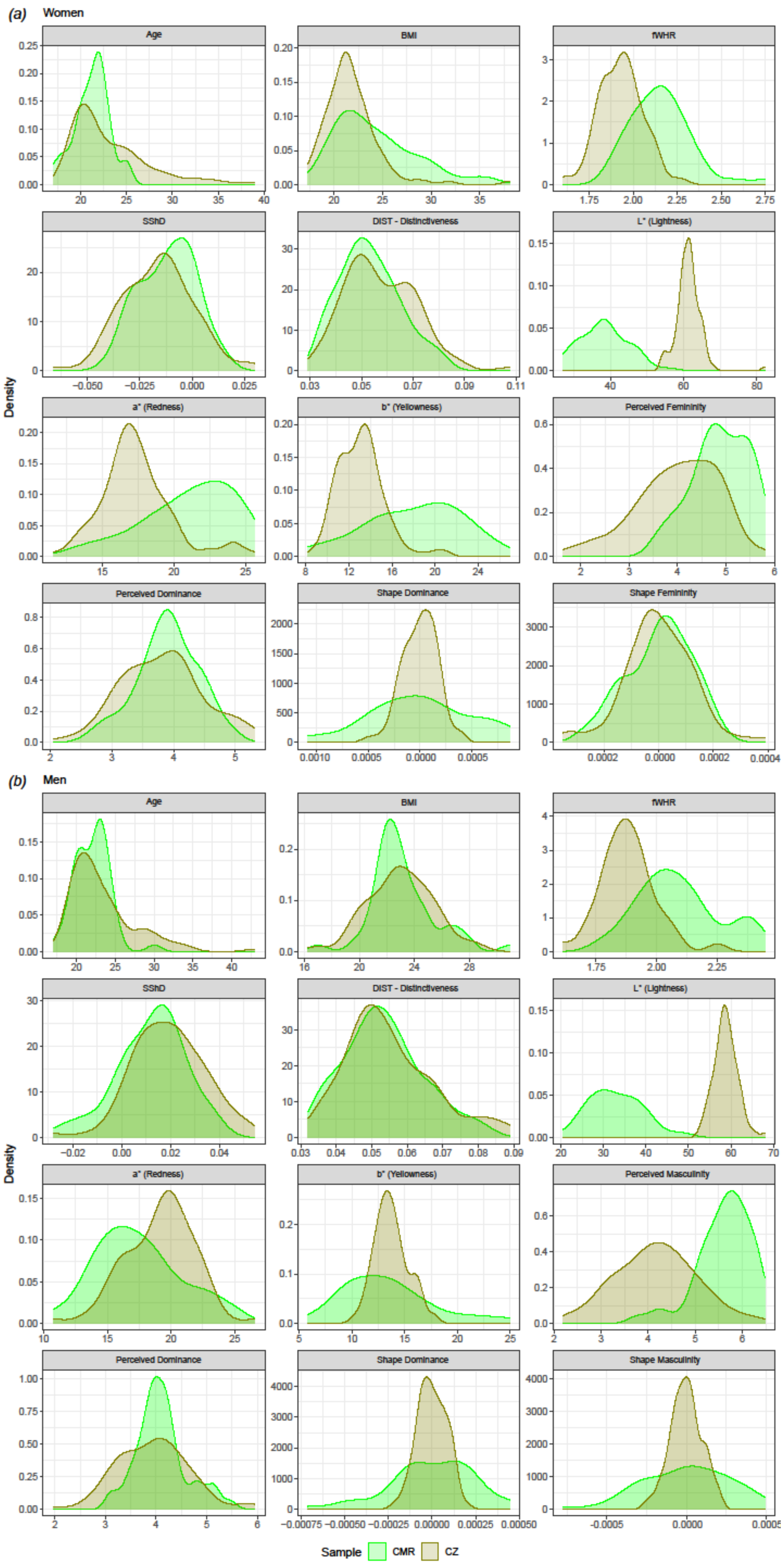

Figure S2 CZ_CMR_Density. Density plots of the distribution of each variable from the analysed samples (Czech and Cameroonian)

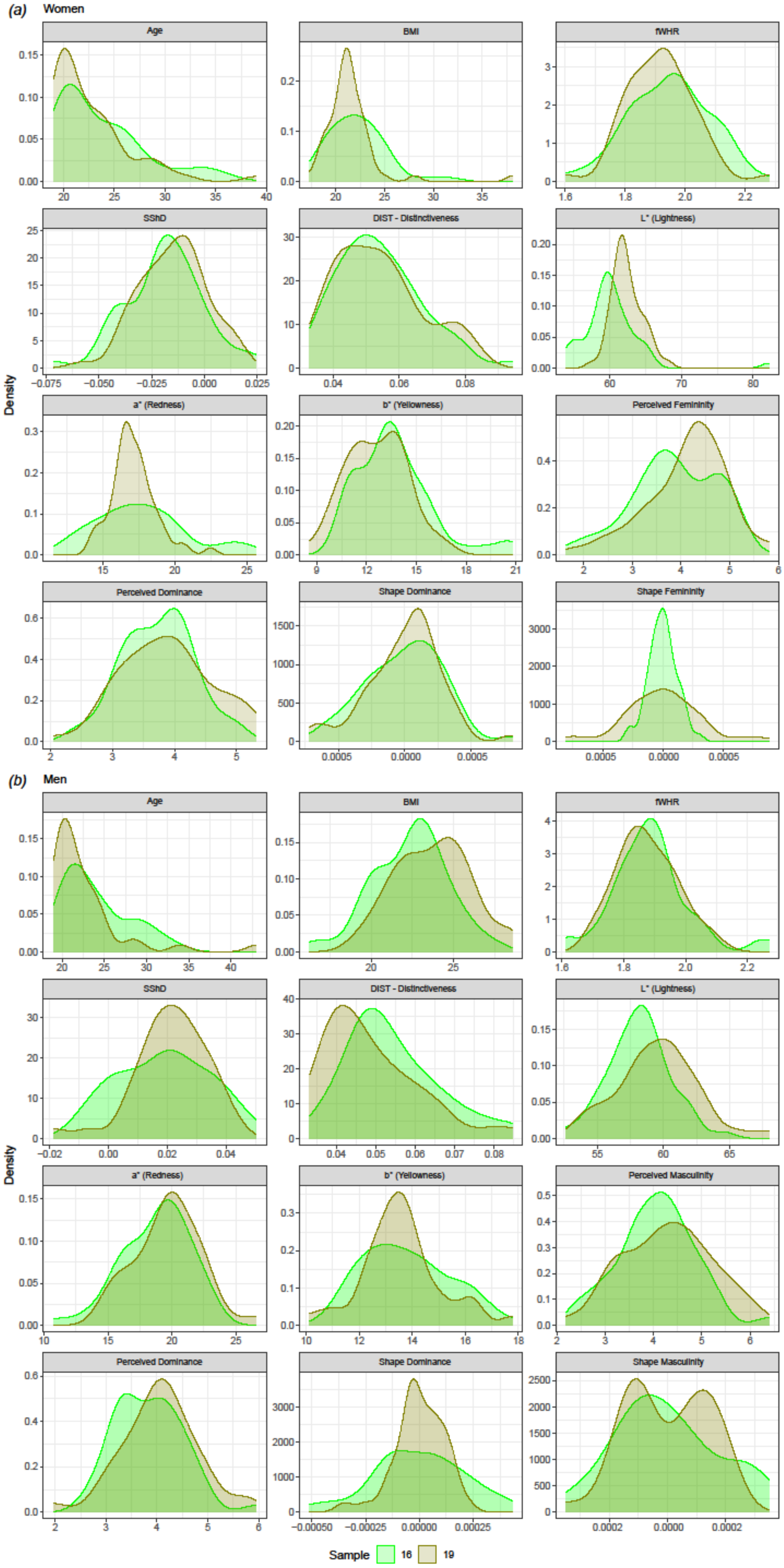

Figure S3 CZ_16_19_Density. Density plots of the distribution of each variable from the Czech sample, separated by the year of the stimuli acquisition (either 2016 or 2019)

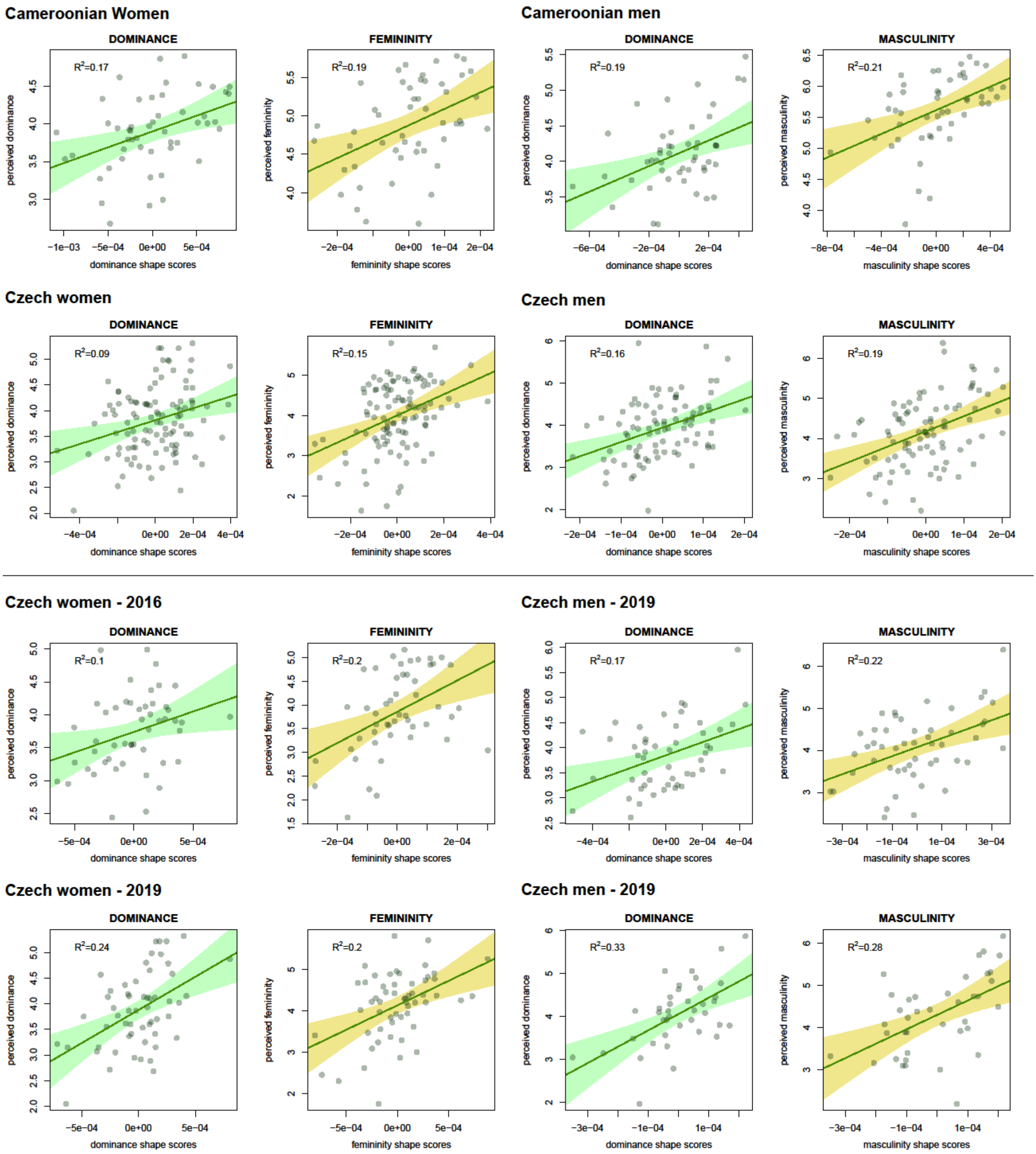

Figure S4_Shape_Regressions_Diagrams. Diagrams of the linear regression between perceived dominance/sex-typicality and its shape component (e.g., between perceived masculinity and shape masculinity)

[Not shown – see below]

Figure S5 Full_Scale Plots of Bayesian Regressions. Basic plots ("plot(coeftab('given model'))") for each of the fitted Bayesian multiple regression, redundant coefficients are omitted. This supplementary figure is also available as "Figure S5, Full_Scale Plots of Bayesian Regressions.pdf" under the link (https://osf.io/mqgxa/?view_only=a42db3ea5d0f4bd3b76e4d614575ba92).

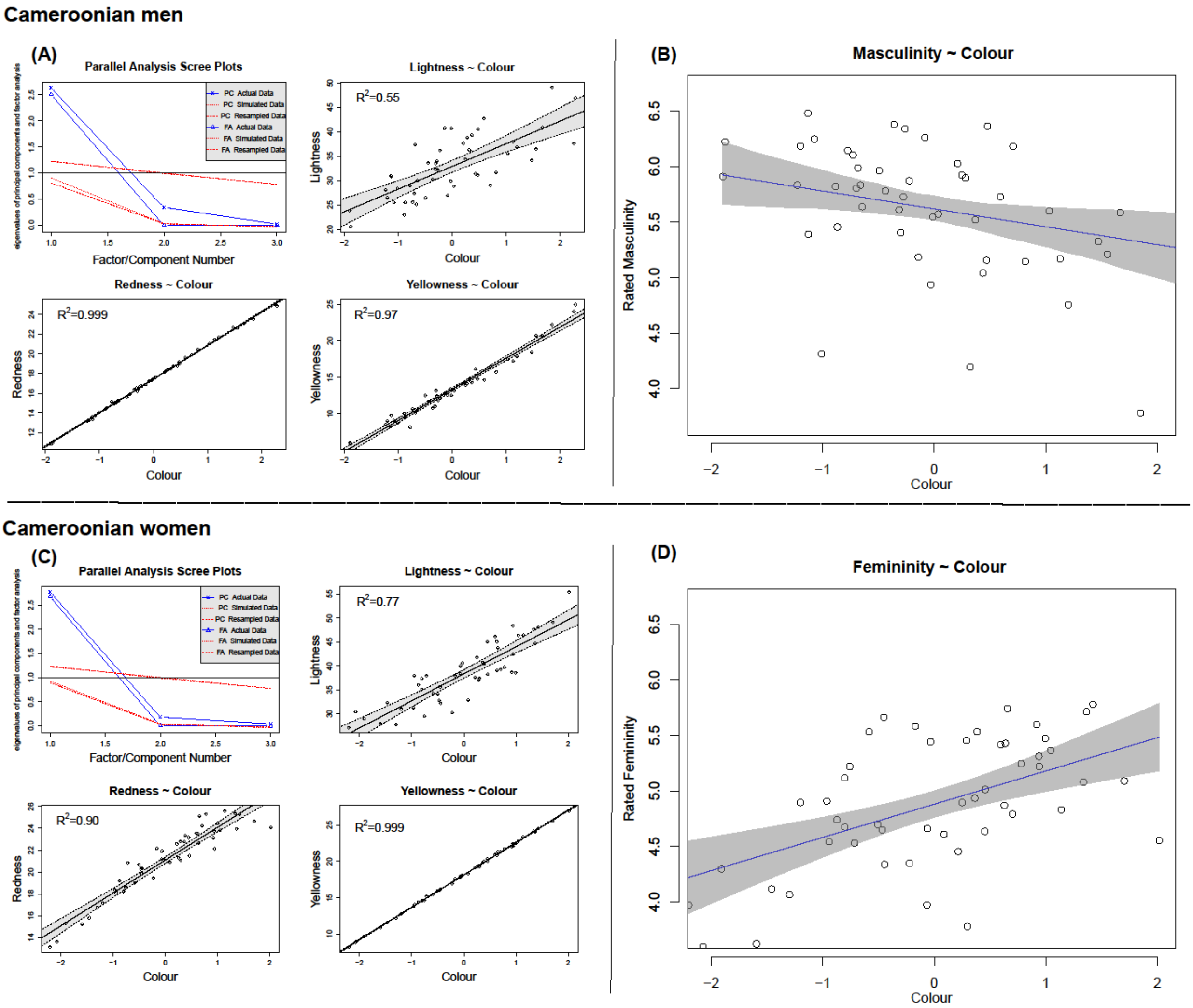

Figure S6_Colour variable in CMR. Associations between colour variables and perceived sex-typicality in Cameroonian sample. Panels on the left (A, C) shows the Parallel Analysis Plot (a steep decline of eigenvalue between one and two underlying factors and plateau further on in the data shows that a single underlying factor may likely explain the variability within the three variables) for the variables CIELab L*, a*, b* and regressions of the resulting variable "colour" on each of the three colour channels. Counterfactual plots on the right (B, D) shows the resulting slope for the partial regression (i.e. graphical representation of the bivariate relationship based on the coefficients from the multiple regression analyses described above). Grey bands represent the 95% confidence/credibility interval. Panels (A, B) describes the sample of men; Panels (C, D) describes the sample of women.

S.3.1. Abbreviations in the Bayesian multiple regression analyses

AVRG, av, iav = distinctiveness of facial configuration (DIST), the relative distance of face configuration from mean facial configuration of a given sample (e.g. Czech women)

TDom/TFem/TMasc (td/tf/tm) = Shape Dominance/Shape Femininity/Shape Masculinity; the shape component of the perceived dominance and sex-typicality (femininity/masculinity)

S.3.1.1. Model syntax

In the scripts (see below and in this folder) and in the basic diagrams of estimated parameters (see in this folder, Figure S4, Table S3), we use abbreviations for the variables and related estimated parameters. These are as follows:

Masc = perceived masculinity

Fem = perceived femininity
Dom = perceived dominance
muM, muF, muD = mean value of the distribution of given variable – masculinity, femininity, dominance
Rho_out = residual covariance between variables within the same multi-variate distribution (perceived dominance and perceived sex-typicality in this set of models)
Sigma_out = standard deviation for a given variable(s) within a distribution
iD = intercept for the perceived dominance
iF = intercept for the perceived femininity
iM = intercept for the perceived masculinity

BaM/BaF = “beta from redness (a) to perceived masculinity or femininity (M/F)” – an estimated slope of a directed bivariate linear association
BbM/BbF = “beta from yellowness (b) to perceived masculinity or femininity (M/F)”
BLM/BLF = “beta from lightness (L) to perceived masculinity or femininity (M/F)”
BavM/BavF = “beta from distinctiveness (av) to perceived masculinity or femininity (M/F)”
BfWM/BfWF = “beta from fWHR (fW) to perceived masculinity or femininity (M/F)”
BShM/BShF = “beta from SShD (Sh) to perceived masculinity or femininity (M/F)”
BBMIM/BBMIF = “beta from BMI to perceived masculinity or femininity (M/F)”
BAgeM/BAgeF = "beta from Age to perceived masculinity or femininity (M/F)"

BaD = "beta from redness (a) to perceived dominance (D)"
BbD = "beta from yellowness (b) to perceived dominance (D)"
BLD = "beta from lightness (L) to perceived dominance (D)"
BavD = "beta from distinctiveness (av) to perceived dominance (D)"
BfWD = "beta from fWHR (fW) to perceived dominance (D)"
BShD = "beta from SShD (Sh) to perceived dominance (D)"
BBMID = "beta from BMI to perceived dominance (D)"
BAgeD = "beta from Age to perceived dominance (D)"

Rho_out ~ lkj_corr(2) = prior for correlation matrix (residual covariance between perceived dominance and masculinity/femininity)
sigma_out ~ exponential(1) = prior for standard deviation for perceived dominance and masculinity/femininity

mua, mub, muL, muAVRG, mufWHR, muSShD = mean value of distribution of given variable – (in row) redness (mua), yellowness (mub), lightness (muL), distinctiveness (muAVRG), relative facial width (mufWHR), and sexual shape dimorphism (muSShD)

ia, ib, iL, iav, ifw, iSh = intercept for (in row) redness (ia), yellowness (ib), Lightness (iL), distinctiveness (iav), fWHR (ifw), SShD (iSh)

BBMIa/BAgea = “beta from BMI/Age to redness (a)”
BBMIb/BAgeb = “beta from BMI/Age to yellowness (b)”
BBMIL/BAgeL = “beta from BMI/Age to lightness (L)”
BBMIav/BAgeav = “beta from BMI/Age to distinctiveness (av)”
BBMIfW/BAgefW = “beta from BMI/Age to relative facial width (fW)”
BBMISh/BAgeSh = “beta from BMI/Age to sexual shape dimorphism (Sh)”

Rho_med ~ lkj_corr(2) = prior for correlation matrix (residual covariance among redness, yellowness, lightness, AVRG, fWHR and SShD, respectively redness, yellowness, lightness, AVRG, fWHR, SShD, shape dominance, and shape sex-typicality)

sigma_med ~ exponential (1) = prior for standard deviations for estimated distribution of redness, yellowness, lightness, AVRG, fWHR and SShD (respectively redness, yellowness, lightness, AVRG, fWHR, SShD, shape dominance, and shape sex-typicality)

muBMI, sigmaBMI = mean value of the distribution of BMI, the standard deviation for BMI
iBMI = intercept for BMI
BAgeBMI = “beta from Age to BMI”

S3.1.2. Colour variable

In Cameroonian samples, we also use the variable “Colour” defined as the underlying factor for L*, a*, b* colour/tone channels; the model syntax is modified accordingly:

BCM/BCF = “beta from colour (C) to perceived masculinity/femininity”
BCD = “beta from colour (C) to perceived dominance”
muC = mean value of the distribution of colour (C)
iC = intercept for colour (C)
BAgeC/BBMIC = "beta from Age to colour"/" beta from BMI to colour"

S3.1.3. Shape Dominance & Shape Sex-typicality

In extended models, we use shape dominance and shape sex-typicality (shape masculinity and shape femininity) as predictors defined within the multi-normal distribution with redness, yellowness, Lightness, fWHR, SShD and AVRG (i.e., facial distinctiveness, DIST). Extended model syntax contains these additional parameters:

TDom = shape dominance
TMasc = shape masculinity
TFem = shape femininity

BtdM/BtdF = “beta from shape dominance (td) to perceived masculinity (M)”/” beta from shape dominance to perceived femininity (F)”
BtdD = “beta from shape dominance (td) to perceived dominance (D)”
BtmM/BtfF = "beta from shape masculinity (tm) to perceived masculinity (M)"/" beta from shape femininity (tf) to perceived femininity (F)"
BtmD/BtfD = "beta from shape masculinity (tm) to perceived dominance (D)"/" beta from shape femininity (tf) to perceived Dominance (D)"

muTDom = mean value of the distribution of shape dominance
muTFem = mean value of the distribution of shape femininity
muTMasc = mean value of the distribution of shape masculinity

itd = intercept for shape dominance
itf = intercept for shape femininity
itm = intercept for shape masculinity

BBMitd = “beta from BMI to shape dominance (td)”
BBMItf = "beta from BMI to shape femininity (tf)"
BBMItm = "beta from BMI to shape masculinity (tm)"
BAgetd = "beta from age to shape dominance (td)"
BAgetf = "beta from age to shape femininity" (tf)
BAgetm = "beta from age to shape masculinity (tm)"

S.3.1.4. A note to residual covariance

Rho_med stands for residual covariance among the variables that were fitted within the same multi_normal distribution. In supplementary tables and diagrams in this file, each residual covariance is reported as Rho_med[X,Y], where X stands for a row, Y stands for a matrix column.

(A) In models with redness (a*), yellowness (b*) and lightness (L*), the order of variables in the residual covariance matrix (Rho_med) is:
1 = redness (a*); 2 = yellowness (b*); 3 = lightness (L*); 4 = Distinctiveness (AVRG); 5 = fWHR; 6 = SShD

(B) In models with colour [C] as underlying factor instead of redness, yellowness, and redness, the order of variables is: 1 = colour [C]; 2 = Distinctiveness (AVRG); 3 = fWHR; 4 = SShD

(C) In models with Shape Dominance and redness (a*), yellowness (b*), and lightness (L*), the order is: 1 = redness (a*); 2 = yellowness (b*); 3 = lightness (L*); 4 = Distinctiveness (AVRG); 5 = fWHR; 6 = SShD; 7 = TDom (shape dominance)

(D) In models with Shape Sex-typicality (masculinity or femininity) and redness (a*), yellowness (b*), and lightness (L*), the order is: 1 = redness (a*); 2 = yellowness (b*); 3 = lightness (L*); 4 = Distinctiveness (AVRG); 5 = fWHR; 6 = SShD; 7 = TFem/TMasc – shape sex-typicality (masculinity or femininity)

(E) In models with both shape dominance and shape sex-typicality (masculinity or femininity) and redness, yellowness, lightness, the order of the variables is: 1 = redness (a*); 2 = yellowness (b*); 3 = lightness (L*); 4 = Distinctiveness (AVRG); 5 = fWHR; 6 = SShD; 7 = TDom (Shape Dominance); 8 = TFem/TMasc = shape sex-typicality (masculinity or femininity)

(F) In models with Shape Dominance and colour (C), the order is:
1 = colour (C); 2 = Distinctiveness (AVRG); 3 = fWHR; 4 = SShD; 5 = TDom (shape dominance)

(G) In models with Shape Sex-typicality (masculinity or femininity) and colour (C), the order is:
1 = colour (C); 2 = Distinctiveness (AVRG); 3 = fWHR; 4 = SShD; 5 TFem/TMasc = shape sex-typicality (masculinity or femininity)

(H) In models with both shape dominance and shape sex-typicality (masculinity or femininity) and colour variable the order is:
1 = colour (C); 2 = Distinctiveness (AVRG); 3 = fWHR; 4 = SShD; 5 = TDom (shape dominance), 6 = shape masculinity or femininity

S.3.2. A guide to the online Supplementary Material folder:

There are four file folders and eight separate stand-alone files in the OSF storage to the project. Some of the files are full-scale version of the Figures shown above. All of the file folders are available under the link: https://osf.io/mqgxa/?view_only=a42db3ea5d0f4bd3b76e4d614575ba92

<u>Separate files</u>:

“Figure S1 CZ_CMR_Density.pdf” – density plots of the distribution of each variable from the analysed samples (Czech and Cameroonian)

“Figure S2 CZ_16_19_Density.pdf” – density plots of the distribution of each variable from the Czech sample, separated by the year of the stimuli acquisition (either 2016 or 2019)

“Figure S3_Shape_Regressions_Diagrams.pdf” – diagrams of the linear regression between perceived dominance/sex-typicality and its shape component (e.g., between perceived masculinity and shape masculinity)

“Figure S4 Multiple Regressions.pdf” – basic plots (“plot(coeftab(‘given model’))”) for each of the fitted Bayesian multiple regression, redundant coefficients are omitted. Note: This Figure (Figure S4) is also available at: https://osf.io/mqgxa/?view_only=a42db3ea5d0f4bd3b76e4d614575ba92. Version located in the aforementioned file folder represents the direct unedited outcome of the R scripts.

“Figure S5_Colour variable in CMR.pdf” – Associations between colour variables and perceived sex-typicality in Cameroonian sample. Panels on the left (A, C) shows the Parallel Analysis Plot (a steep decline of eigenvalue between one and two underlying factors and plateau further on in the data shows that a single underlying factor may likely explain the variability within the three variables) for the variables CIELab L*, a*, b* and regressions of the resulting variable “colour” on each of the three colour channels. Counterfactual plots on the right (B, D) shows the resulting slope for the partial regression (i.e. graphical representation of the bivariate relationship based on the coefficients from the multiple regression analyses described above). Grey bands represent the 95% confidence/credibility interval. Panels (A, B) describes the sample of men; Panels (C, D) describes the sample of women.

“Table S1 Descriptive statistics per stimuli sample.xlsx” – Detailed report of descriptive statistics for each sample (Czech samples are reported both as 2016 and 2019 combined sample and as separate samples) and each variable

“Table S2 Descriptive statistics per raters sample.xlsx” – Descriptive statistics for samples of raters

“Table S3_Multiple Regression Coefficients.xlsx” – Estimated parameters for each fitted model (in a row) and each sample (in a column)

Note: For optimal view, the .xlsx files should be downloaded and opened with MS Office Excel.

File folders (at https://osf.io/mqgxa/?view_only=a42db3ea5d0f4bd3b76e4d614575ba92):

“**Bayesian Multiple Regressions [.Rdata objects & separate figures]**” – The folder contains (a) tables of coefficients for each model (mean, SD, 89% and 95% credibility intervals) for each estimated model parameter; (b) basic diagram for each of the fitted model (c) the models themselves, saved as R Workspace (.Rdata) files. The files were used to draw the diagrams described above.

“**Comparison across Multiple Regressions - Plots**” – The folder contains diagrams that compare results across models with different variables. We fitted models with Shape Dominance and Shape Sex-Typicality and models with either one of those shape variables or without them. It may be hard to follow estimated model parameters and their variance across different table(s)/diagrams(s); therefore, we draw diagrams that report all the models at once, with corresponding associations plotted next to each other.
The word “_Big” in the file’s name denotes a diagram reporting all the coefficients, some of which may be redundant and/or irrelevant. The word “_small” denotes that the diagram plots only the coefficients of interest.

The folder “**Original Data**” contains .csv tables with all the variables that entered the analyses (for the purpose of re-analyses and additional analyses).

"**Scripts**" – The folder stores R scripts, as follows (in alphabetic order):

"Bayes_CZ_16_19_Subsets.R" Script for the analyses on the separate Czech datasets (2016 and 2019).
"CZ_CMR_Graphs_Across_Models.R" Script with commands to draw basic coeftab plots to compare the mean of each parameter sampled distribution and its credibility intervals across the fitted models. In each sample, we compare estimated parameters from the model (i) without shape dominance and shape sex-typicality; (ii) with either shape dominance OR shape sex-typicality; (ii) with both the shape dominance and shape sex-typicality.
"Demography_Exploration.R" Script contains commands to obtain (a) Density plots of the variables' distributions; (b) Table with mean, SD, range, skewness and kurtosis for variables in each sample; (c) Correlation and Partial correlation networks.
"ShapeDomSext_Bayes_Models.R" Script for the main analyses that are reported in the paper.

Notes:

- The scripts require the Rethinking Package, ver. 2.13. The current version of the package requires Stan (for model syntax and sampling), ver. 2.21.2. to be installed either. Interested reader should visit https://github.com/rmcelreath/rethinking for more info.
- Diagrams were further edited in InkScape (ver. 1.02) and CorelDraw.

S.4. References (supplementary materials only):

Axelsson, J., Sundelin, T., Olsson, M. J., Sorjonen, K., Axelsson, C., Lasselin, J., & Lekander, M. (2018). Identification of acutely sick people and facial cues of sickness. Proceedings of the Royal Society B: Biological Sciences, 285(1870), 3–9. https://doi.org/10.1098/rspb.2017.2430

Bird, B. M., Cid Jofré, V. S., Geniole, S. N., Welker, K. M., Zilioli, S., Maestripieri, D., Arnocky, S., & Carré, J. M. (2016). Does the facial width-to-height ratio map onto variability in men's testosterone concentrations? Evolution and Human Behavior, 37(5), 392–398. https://doi.org/10.1016/j.evolhumbehav.2016.03.004

Carre, J. M., & McCormick, C. M. (2008). In your face: facial metrics predict aggressive behaviour in the laboratory and in varsity and professional hockey players. Proceedings of the Royal Society B: Biological Sciences, 275(1651), 2651–2656. https://doi.org/10.1098/rspb.2008.0873

Coetzee, V., Faerber, S. J., Greeff, J. M., Lefevre, C. E., Re, D. E., & Perrett, D. I. (2012). African Perceptions of Female Attractiveness. PLoS ONE, 7(10), 3–8. https://doi.org/10.1371/journal.pone.0048116

de Jager, S., Coetzee, N., & Coetzee, V. (2018). Facial adiposity, attractiveness, and health: A review. Frontiers in Psychology, 9(DEC), 1–16. https://doi.org/10.3389/fpsyg.2018.02562

Dixson, B. J. (2017). Facial Width to Height Ratio and Dominance. In Encyclopedia of Evolutionary Psychological Science. https://doi.org/10.1007/978-3-319-16999-6

Geniole, S. N., Denson, T. F., Dixson, B. J., Carré, J. M., & McCormick, C. M. (2015). Evidence from meta-analyses of the facial width-to-height ratio as an evolved cue of threat. PLoS ONE, 10(7), 1–18. https://doi.org/10.1371/journal.pone.0132726

Haselhuhn, M. P., & Wong, E. M. (2012). Bad to the bone: Facial structure predicts unethical behaviour. Proceedings of the Royal Society B: Biological Sciences, 279(1728), 571–576. https://doi.org/10.1098/rspb.2011.1193

Kalick, S. M., Zebrowitz, L. A., Langlois, J. H., & Johnson, R. M. (1998). Does human facial attractiveness honestly advertise health? Longitudinal Data on an Evolutionary Question.

Psychological Science, 9(1), 8–13. https://doi.org/10.1111/1467-9280.00002

Kleisner, K., Chvátalová, V., & Flegr, J. (2014). Perceived Intelligence Is Associated with Measured Intelligence in Men but Not Women. PLoS ONE, 9(3), e81237. https://doi.org/10.1371/journal.pone.0081237

Kordsmeyer, T. L., Freund, D., Pita, S. R., Jünger, J., & Penke, L. (2019). Further Evidence that Facial Width-to-Height Ratio and Global Facial Masculinity Are Not Positively Associated with Testosterone Levels. Adaptive Human Behavior and Physiology, 5(2), 117–130. https://doi.org/10.1007/s40750-018-0105-4

Kramer, R. S. S. (2015). Facial width-to-height ratio in a large sample of commonwealth games athletes. Evolutionary Psychology, 13(1), 197–209. https://doi.org/10.1177/147470491501300112

Lefevre, C. E., & Lewis, G. J. (2014). Perceiving Aggression from Facial Structure: Further Evidence for a Positive Association with Facial Width-to-Height Ratio and Masculinity, but not for Moderation by Self-Reported Dominance. European Journal of Personality, 28(6), 530–537. https://doi.org/10.1002/per.1942

Lefevre, C. E., Lewis, G. J., Perrett, D. I., & Penke, L. (2013). Telling facial metrics: facial width is associated with testosterone levels in men. Evolution and Human Behavior, 34(4), 273–279. https://doi.org/10.1016/j.evolhumbehav.2013.03.005

Linke, L., Saribay, S. A., & Kleisner, K. (2016). Perceived trustworthiness is associated with position in a corporate hierarchy. Personality and Individual Differences, 99(September), 22–27. https://doi.org/10.1016/j.paid.2016.04.076

McElreath, R. (2020). rethinking: Statistical Rethinking book package. R package version 2.13. (2.13). https://github.com/rmcelreath/rethinking

McLaren, K. (1976). The Development of the CIE 1976 (L " a " b ") Uniform Colour Space and Colour-difference Formula. Journal of the Society of Dyers and Colourists, 92(9), 338–341. https://doi.org/10.1111/j.1478-4408.1976.tb03301.x

Nábělková, M. (2007). Closely-related languages in contact : Czech , Slovak , '" Czechoslovak ."' International Journal of The Sociology of Language, 183, 53–73. https://doi.org/10.1515/IJSL.2007.004

Oosterhof, N. N., & Todorov, A. (2008). The functional basis of face evaluation. Proceedings of the National Academy of Sciences of the United States of America, 105(32), 11087–11092. https://doi.org/10.1073/pnas.0805664105

Over, H., & Cook, R. (2018). Where do spontaneous first impressions of faces come from? Cognition, 170(April 2017), 190–200. https://doi.org/10.1016/j.cognition.2017.10.002

Özener, B. (2012). Facial width-to-height ratio in a Turkish population is not sexually dimorphic and is unrelated to aggressive behavior. Evolution and Human Behavior, 33(3), 169–173. https://doi.org/10.1016/j.evolhumbehav.2011.08.001

R Core Team. (2021). R: A language and environment for statistical computing. (4.0.3). R Foundation for Statistical Computing. https://www.r-project.org/

Revelle, W. (2018). psych: Procedures for Personality and Psychological Research. Northwestern University. https://cran.r-project.org/package=psych Version = 1.8.12.

Rhodes, G., Zebrowitz, L. A., Clark, A., Kalick, S. M., Hightower, A., & McKay, R. (2001). Do facial averageness and symmetry signal health? Evolution and Human Behavior, 22(1), 31–46.

https://doi.org/10.1016/S1090-5138(00)00060-X

Schneider, C. A., Rasband, W. S., & Eliceiri, K. W. (2012). NIH Image to ImageJ: 25 years of image analysis. Nature Methods, 9(7), 671–675. https://doi.org/10.1038/nmeth.2089

Sell, A., Cosmides, L., Tooby, J., Sznycer, D., Von Rueden, C., & Gurven, M. (2009). Human adaptations for the visual assessment of strength and fighting ability from the body and face. Proceedings of the Royal Society B: Biological Sciences, 276(1656), 575–584. https://doi.org/10.1098/rspb.2008.1177

Shrout, P. E., & Fleiss, J. L. (1979). Intraclass correlations: uses in assessing rater reliability. Psychological Bulletin, 86(2), 420–428. http://www.ncbi.nlm.nih.gov/pubmed/18839484

Stan Development Team. (2020). RStan: the R interface to Stan. R package version 2.21.2. (2.21.2.). http://mc-stan.org/

Stirrat, M. R., & Perrett, D. I. (2010). Valid Facial Cues to Cooperation and Trust: Male Facial Width and Trustworthiness. Psychological Science, 21(3), 349–354. https://doi.org/10.1177/0956797610362647

Stolier, R. M., Hehman, E., Keller, M. D., Walker, M., & Freeman, J. B. (2018). The conceptual structure of face impressions. Proceedings of the National Academy of Sciences of the United States of America, 115(37), 9210–9215. https://doi.org/10.1073/pnas.1807222115

Todorov, A., Pakrashi, M., & Oosterhof, N. N. (2009). Evaluating faces on trustworthiness after minimal time exposure. Social Cognition, 27(6), 813–833. https://doi.org/https://doi.org/10.1521/soco.2009.27.6.813

Třebický, V., Fialová, J., Kleisner, K., & Havlíček, J. (2016). Focal length affects depicted shape and perception of facial images. PLoS ONE, 11(2), e0149313. https://doi.org/10.1371/journal.pone.0149313

Třebický, V., Fialová, J., Kleisner, K., Roberts, S. C., Little, A. C., & Havlíček, J. (2015). Further evidence for links between facial width-to-height ratio and fighting success: Commentary on Zilioli et al. (2014). Aggressive Behavior, 41(4), 331–334. https://doi.org/10.1002/ab.21559

Třebický, V., Havlíček, J., Roberts, S. C., Little, A. C., & Kleisner, K. (2013). Perceived Aggressiveness Predicts Fighting Performance in Mixed-Martial-Arts Fighters. Psychological Science, 24(9), 1664–1672. https://doi.org/10.1177/0956797613477117

Wang, D., Nair, K., Kouchaki, M., Zajac, E. J., & Zhao, X. (2019). A Case of Evolutionary Mismatch? Why Facial Width-to-Height Ratio May Not Predict Behavioral Tendencies. Psychological Science, 30(7), 1074–1081. https://doi.org/10.1177/0956797619849928

Willis, J., & Todorov, A. (2006). First Impressions: Making up Your Mind after a 100-ms Exposure to a Face. Psychological Science, 17(7), 592–598. https://doi.org/DOI: 10.1111/j.1467-9280.2006.01750.x

Zebrowitz, L. A. (2006). Finally, faces find favor. Social Cognition, 24(5), 657–701. https://doi.org/10.1521/soco.2006.24.5.657

Figure S5 (see the pages below):

[A] Cameroonian men − L*a*b* as separate variables, without ShDom, ShMasc
Abbreviations are explained in S.3.1 in 'Supplementary texts.pdf'

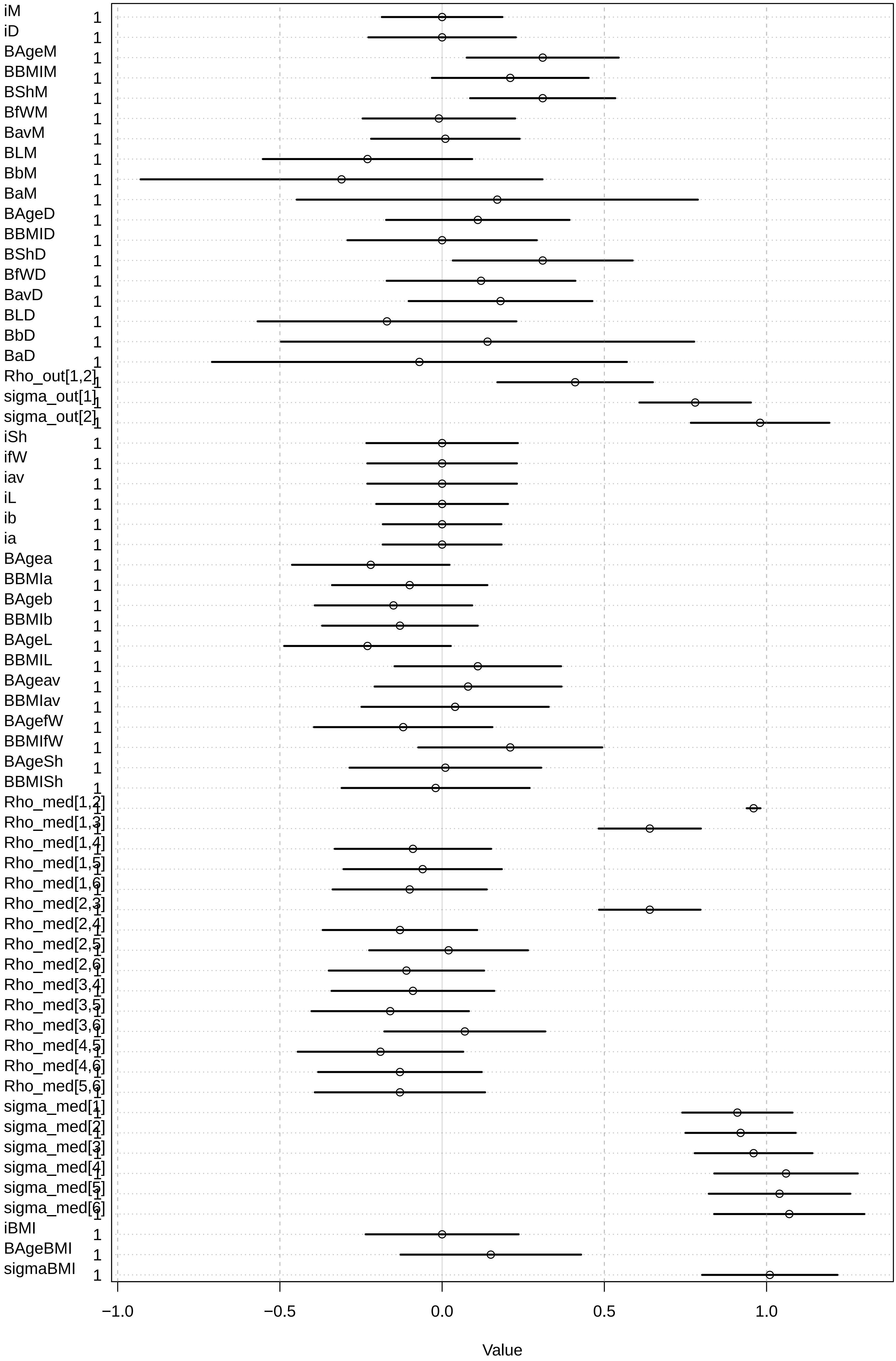

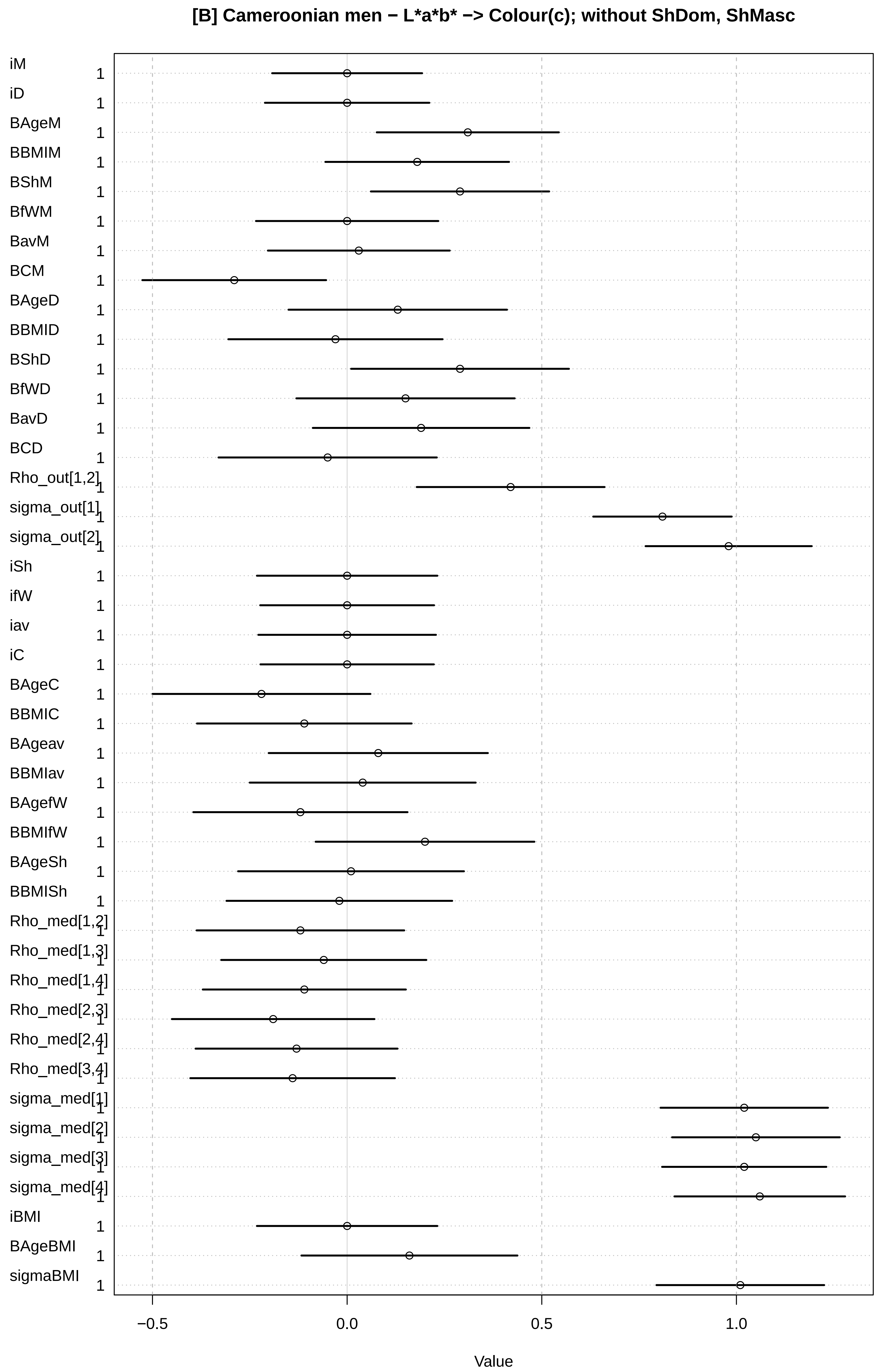

[B] Cameroonian men − L*a*b* −> Colour(c); without ShDom, ShMasc
iM
iD
BAgeM
BBMIM
BShM
BfWM
BavM
BCM
BAgeD
BBMID
BShD
BfWD
BavD
BCD
Rho_out[1,2]
sigma_out[1]
sigma_out[2]
iSh
ifW
iav
iC
BAgeC
BBMIC
BAgeav
BBMIav
BAgefW
BBMIfW
BAgeSh
BBMISh
Rho_med[1,2]
Rho_med[1,3]
Rho_med[1,4]
Rho_med[2,3]
Rho_med[2,4]
Rho_med[3,4]
sigma_med[1]
sigma_med[2]
sigma_med[3]
sigma_med[4]
iBMI
BAgeBMI
sigmaBMI
−0.5
0.0
0.5
1.0
Value

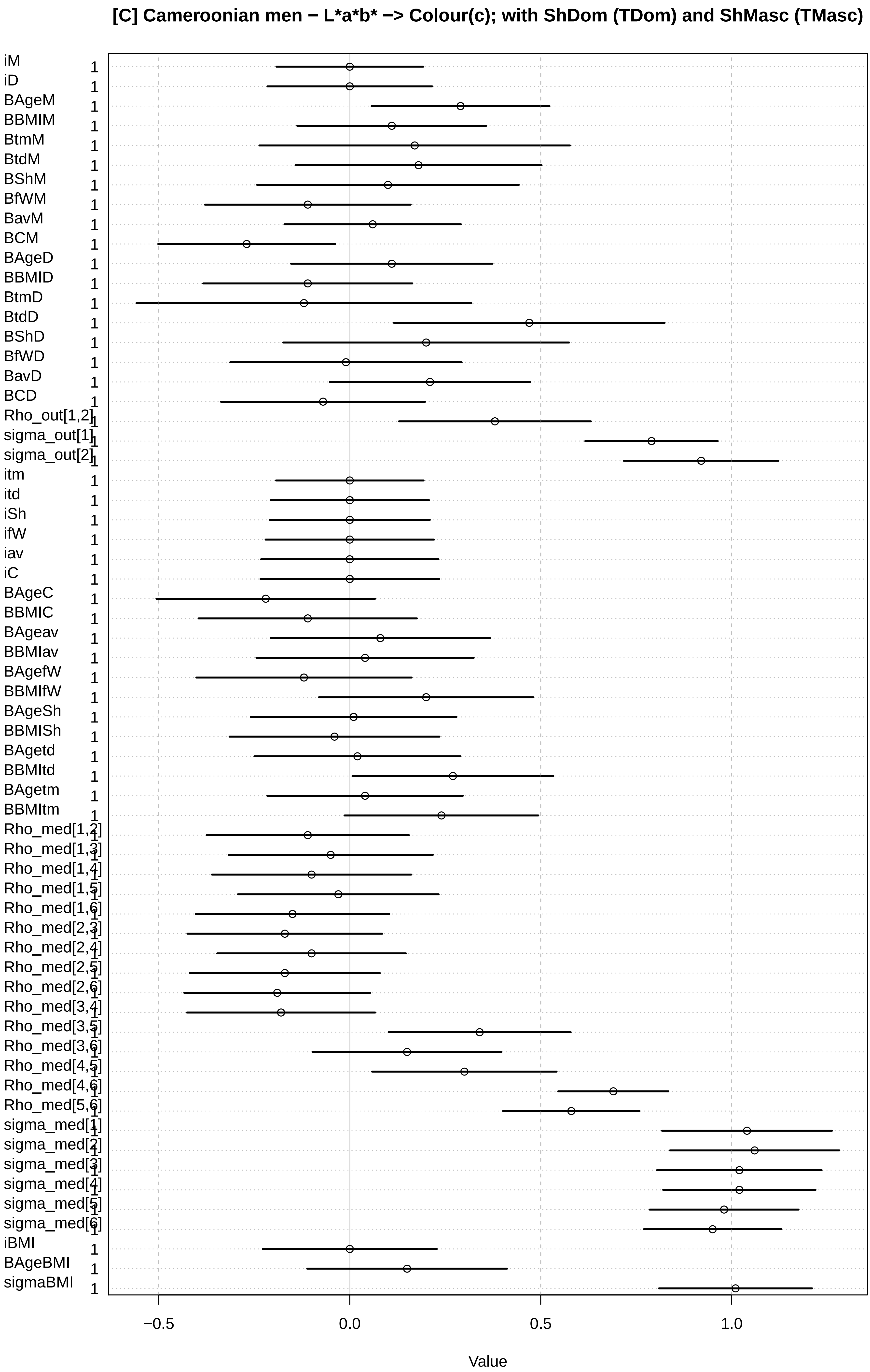
[C] Cameroonian men − L*a*b* −> Colour(c); with ShDom (TDom) and ShMasc (TMasc)
iM
iD
BAgeM
BBMIM
BtmM
BtdM
BShM
BfWM
BavM
BCM
BAgeD
BBMID
BtmD
BtdD
BShD
BfWD
BavD
BCD
Rho_out[1,2]
sigma_out[1]
sigma_out[2]
itm
itd
iSh
ifW
iav
iC
BAgeC
BBMIC
BAgeav
BBMIav
BAgefW
BBMIfW
BAgeSh
BBMISh
BAgetd
BBMItd
BAgetm
BBMItm
Rho_med[1,2]
Rho_med[1,3]
Rho_med[1,4]
Rho_med[1,5]
Rho_med[1,6]
Rho_med[2,3]
Rho_med[2,4]
Rho_med[2,5]
Rho_med[2,6]
Rho_med[3,4]
Rho_med[3,5]
Rho_med[3,6]
Rho_med[4,5]
Rho_med[4,6]
Rho_med[5,6]
sigma_med[1]
sigma_med[2]
sigma_med[3]
sigma_med[4]
sigma_med[5]
sigma_med[6]
iBMI
BAgeBMI
sigmaBMI
−0.5
0.0
0.5
1.0
Value

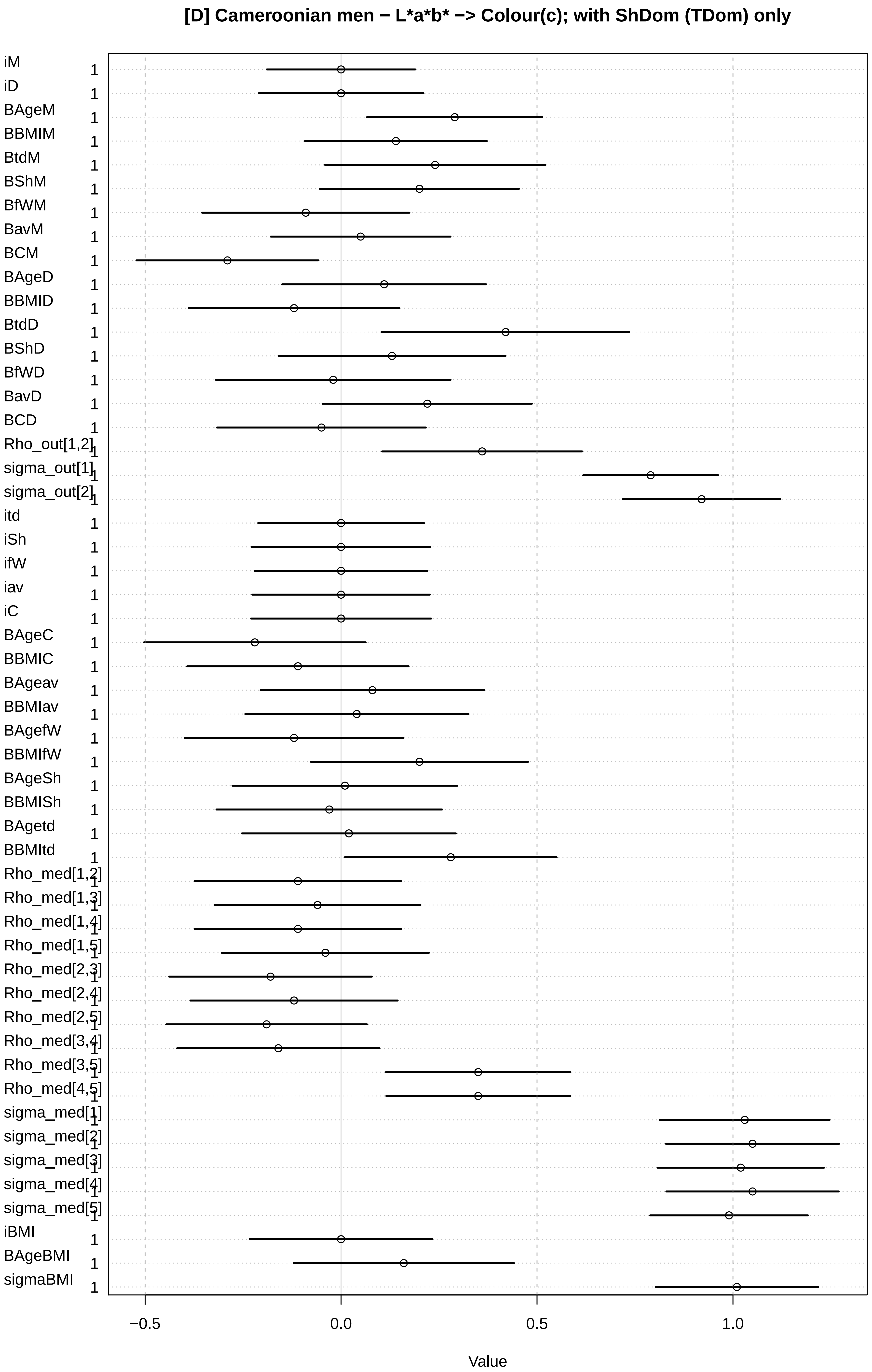

[D] Cameroonian men − L*a*b* −> Colour(c); with ShDom (TDom) only
iM
iD
BAgeM
BBMIM
BtdM
BShM
BfWM
BavM
BCM
BAgeD
BBMID
BtdD
BShD
BfWD
BavD
BCD
Rho_out[1,2]
sigma_out[1]
sigma_out[2]
itd
iSh
ifW
iav
iC
BAgeC
BBMIC
BAgeav
BBMIav
BAgefW
BBMIfW
BAgeSh
BBMISh
BAgetd
BBMItd
Rho_med[1,2]
Rho_med[1,3]
Rho_med[1,4]
Rho_med[1,5]
Rho_med[2,3]
Rho_med[2,4]
Rho_med[2,5]
Rho_med[3,4]
Rho_med[3,5]
Rho_med[4,5]
sigma_med[1]
sigma_med[2]
sigma_med[3]
sigma_med[4]
sigma_med[5]
iBMI
BAgeBMI
sigmaBMI
−0.5
0.0
0.5
1.0
Value

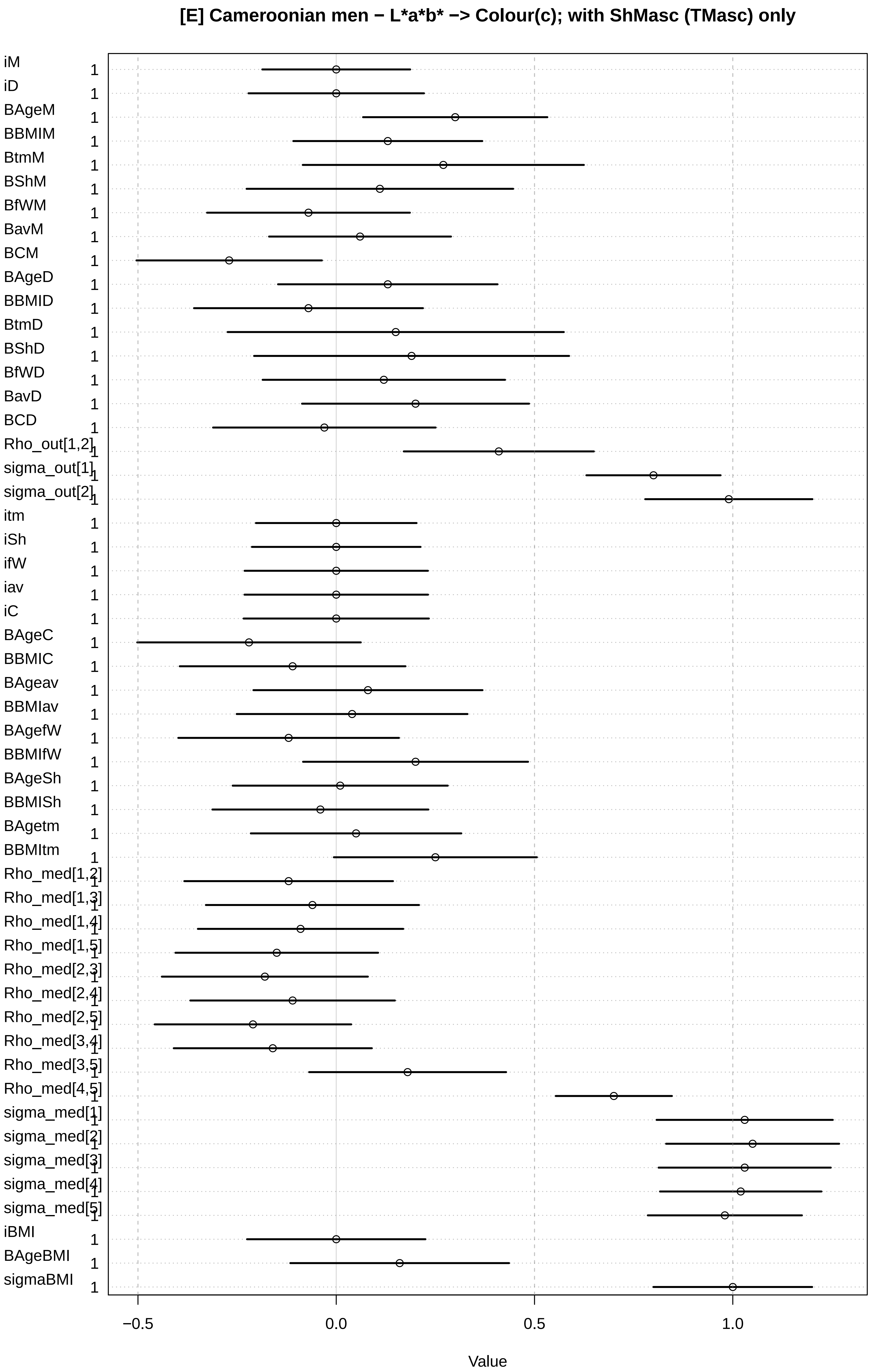
[E] Cameroonian men − L*a*b* −> Colour(c); with ShMasc (TMasc) only
iM
iD
BAgeM
BBMIM
BtmM
BShM
BfWM
BavM
BCM
BAgeD
BBMID
BtmD
BShD
BfWD
BavD
BCD
Rho_out[1,2]
sigma_out[1]
sigma_out[2]
itm
iSh
ifW
iav
iC
BAgeC
BBMIC
BAgeav
BBMIav
BAgefW
BBMIfW
BAgeSh
BBMISh
BAgetm
BBMItm
Rho_med[1,2]
Rho_med[1,3]
Rho_med[1,4]
Rho_med[1,5]
Rho_med[2,3]
Rho_med[2,4]
Rho_med[2,5]
Rho_med[3,4]
Rho_med[3,5]
Rho_med[4,5]
sigma_med[1]
sigma_med[2]
sigma_med[3]
sigma_med[4]
sigma_med[5]
iBMI
BAgeBMI
sigmaBMI
−0.5
0.0
0.5
1.0
Value

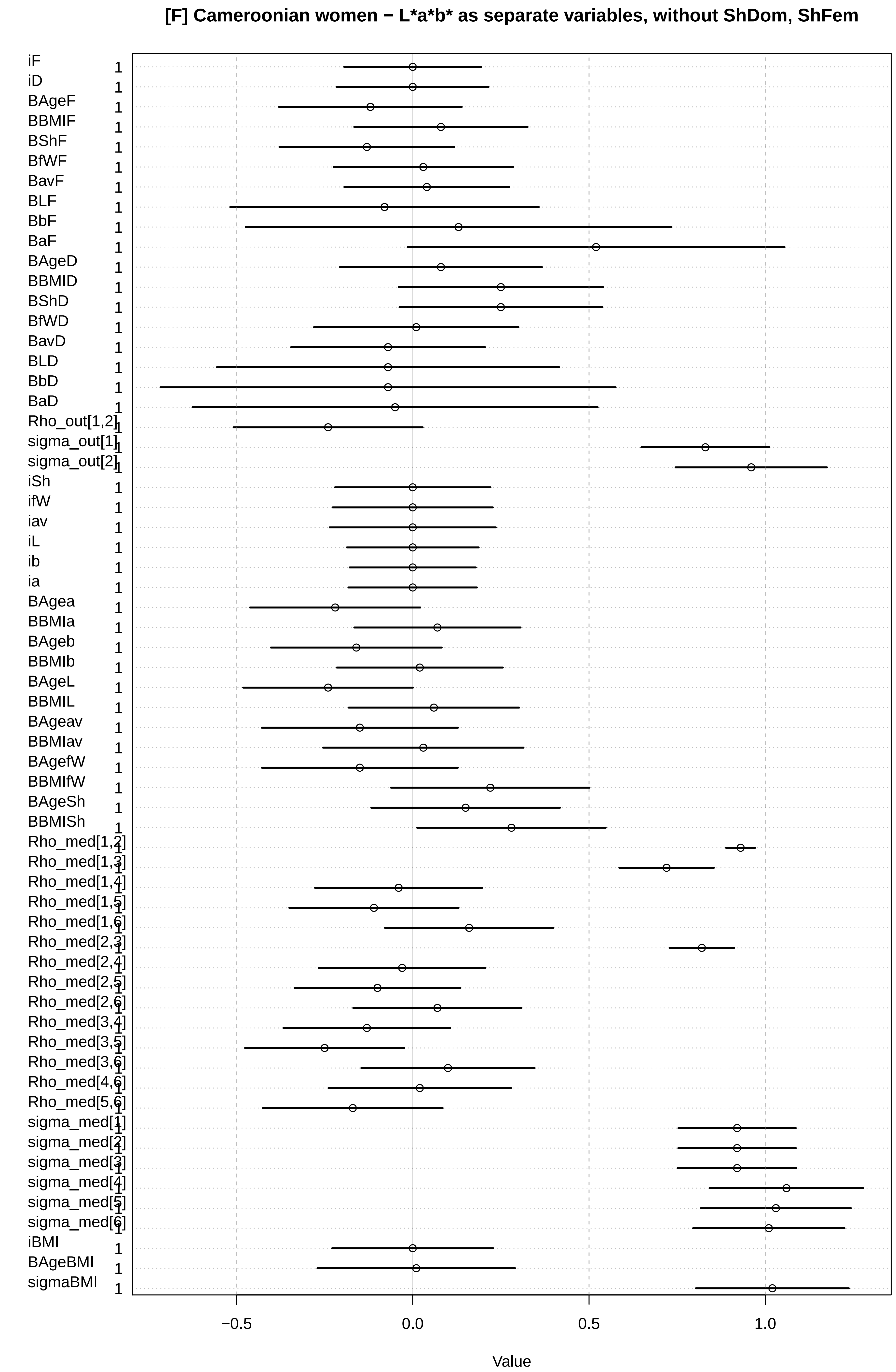
[F] Cameroonian women − L*a*b* as separate variables, without ShDom, ShFem
iF
iD
BAgeF
BBMIF
BShF
BfWF
BavF
BLF
BbF
BaF
BAgeD
BBMID
BShD
BfWD
BavD
BLD
BbD
BaD
Rho_out[1,2]
sigma_out[1]
sigma_out[2]
iSh
ifW
iav
iL
ib
ia
BAgea
BBMIa
BAgeb
BBMIb
BAgeL
BBMIL
BAgeav
BBMIav
BAgefW
BBMIfW
BAgeSh
BBMISh
Rho_med[1,2]
Rho_med[1,3]
Rho_med[1,4]
Rho_med[1,5]
Rho_med[1,6]
Rho_med[2,3]
Rho_med[2,4]
Rho_med[2,5]
Rho_med[2,6]
Rho_med[3,4]
Rho_med[3,5]
Rho_med[3,6]
Rho_med[4,6]
Rho_med[5,6]
sigma_med[1]
sigma_med[2]
sigma_med[3]
sigma_med[4]
sigma_med[5]
sigma_med[6]
iBMI
BAgeBMI
sigmaBMI
−0.5
0.0
0.5
1.0
Value

[G] Cameroonian women − L*a*b* −> Colour(c); without ShDom, ShFem

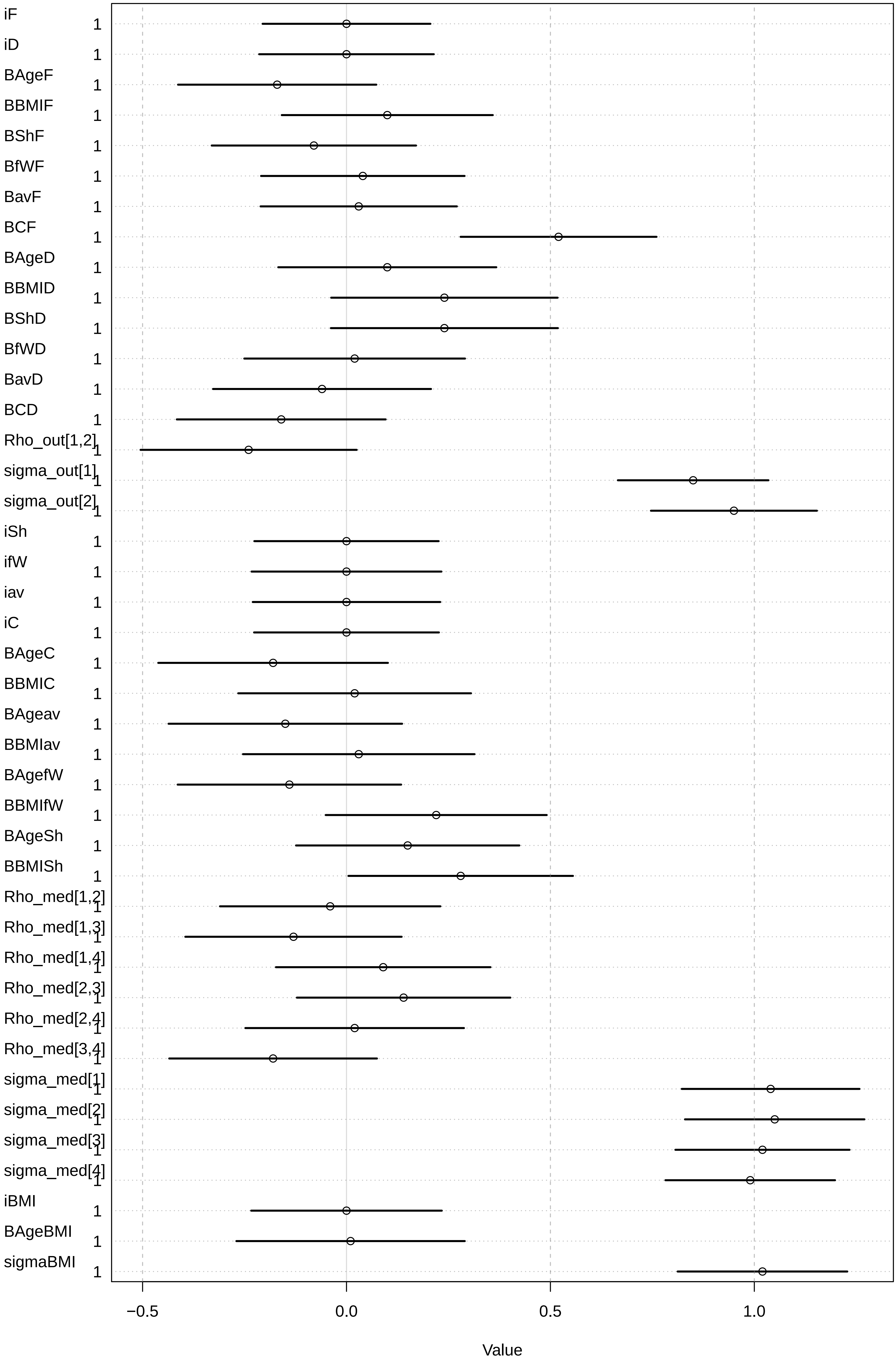

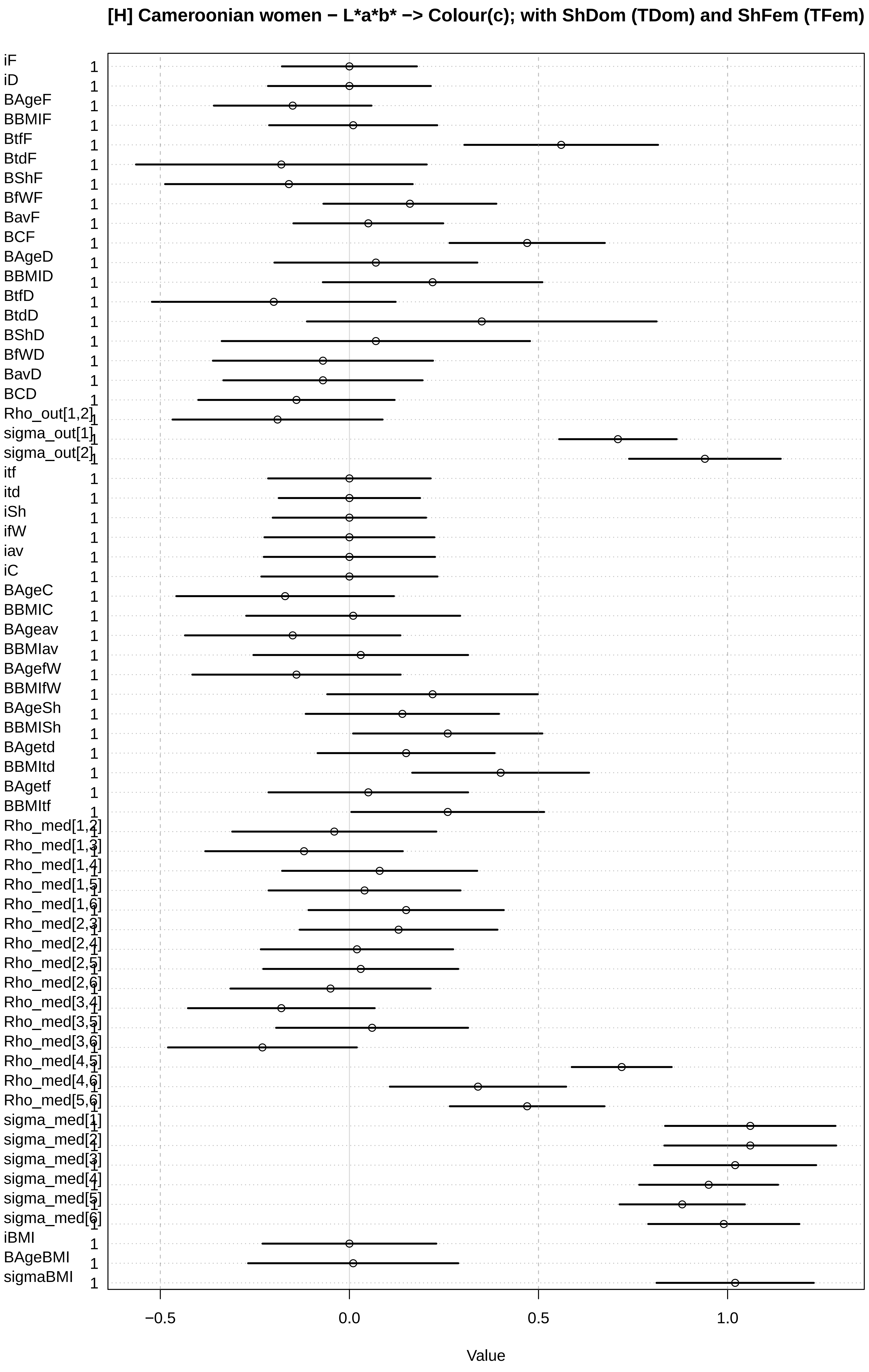

[H] Cameroonian women − L*a*b* −> Colour(c); with ShDom (TDom) and ShFem (TFem)
iF
iD
BAgeF
BBMIF
BtfF
BtdF
BShF
BfWF
BavF
BCF
BAgeD
BBMID
BtfD
BtdD
BShD
BfWD
BavD
BCD
Rho_out[1,2]
sigma_out[1]
sigma_out[2]
itf
itd
iSh
ifW
iav
iC
BAgeC
BBMIC
BAgeav
BBMIav
BAgefW
BBMIfW
BAgeSh
BBMISh
BAgetd
BBMItd
BAgetf
BBMItf
Rho_med[1,2]
Rho_med[1,3]
Rho_med[1,4]
Rho_med[1,5]
Rho_med[1,6]
Rho_med[2,3]
Rho_med[2,4]
Rho_med[2,5]
Rho_med[2,6]
Rho_med[3,4]
Rho_med[3,5]
Rho_med[3,6]
Rho_med[4,5]
Rho_med[4,6]
Rho_med[5,6]
sigma_med[1]
sigma_med[2]
sigma_med[3]
sigma_med[4]
sigma_med[5]
sigma_med[6]
iBMI
BAgeBMI
sigmaBMI
−0.5
0.0
0.5
1.0
Value

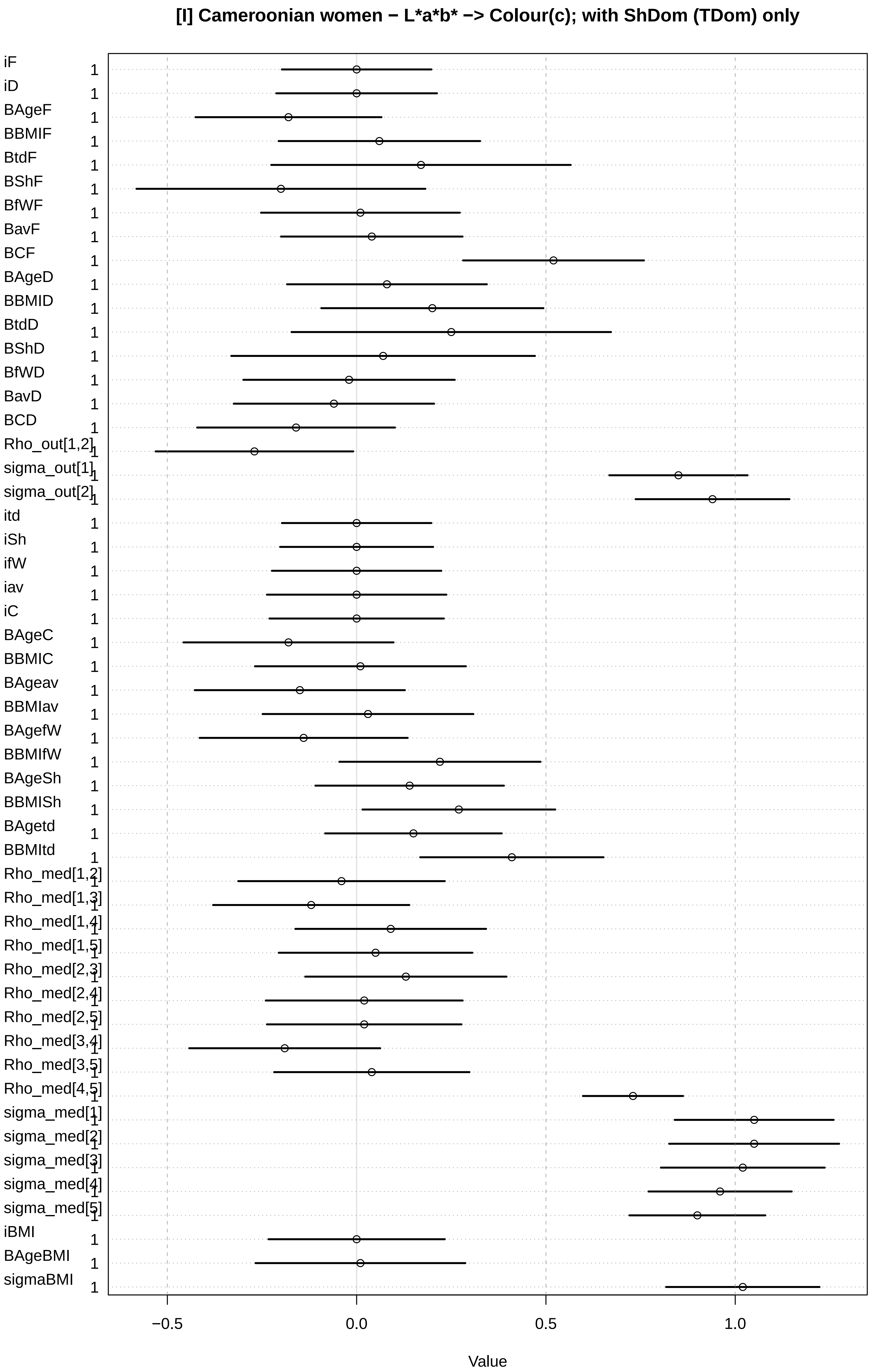

[I] Cameroonian women − L*a*b* −> Colour(c); with ShDom (TDom) only
iF
iD
BAgeF
BBMIF
BtdF
BShF
BfWF
BavF
BCF
BAgeD
BBMID
BtdD
BShD
BfWD
BavD
BCD
Rho_out[1,2]
sigma_out[1]
sigma_out[2]
itd
iSh
ifW
iav
iC
BAgeC
BBMIC
BAgeav
BBMIav
BAgefW
BBMIfW
BAgeSh
BBMISh
BAgetd
BBMItd
Rho_med[1,2]
Rho_med[1,3]
Rho_med[1,4]
Rho_med[1,5]
Rho_med[2,3]
Rho_med[2,4]
Rho_med[2,5]
Rho_med[3,4]
Rho_med[3,5]
Rho_med[4,5]
sigma_med[1]
sigma_med[2]
sigma_med[3]
sigma_med[4]
sigma_med[5]
iBMI
BAgeBMI
sigmaBMI
−0.5
0.0
0.5
1.0
Value

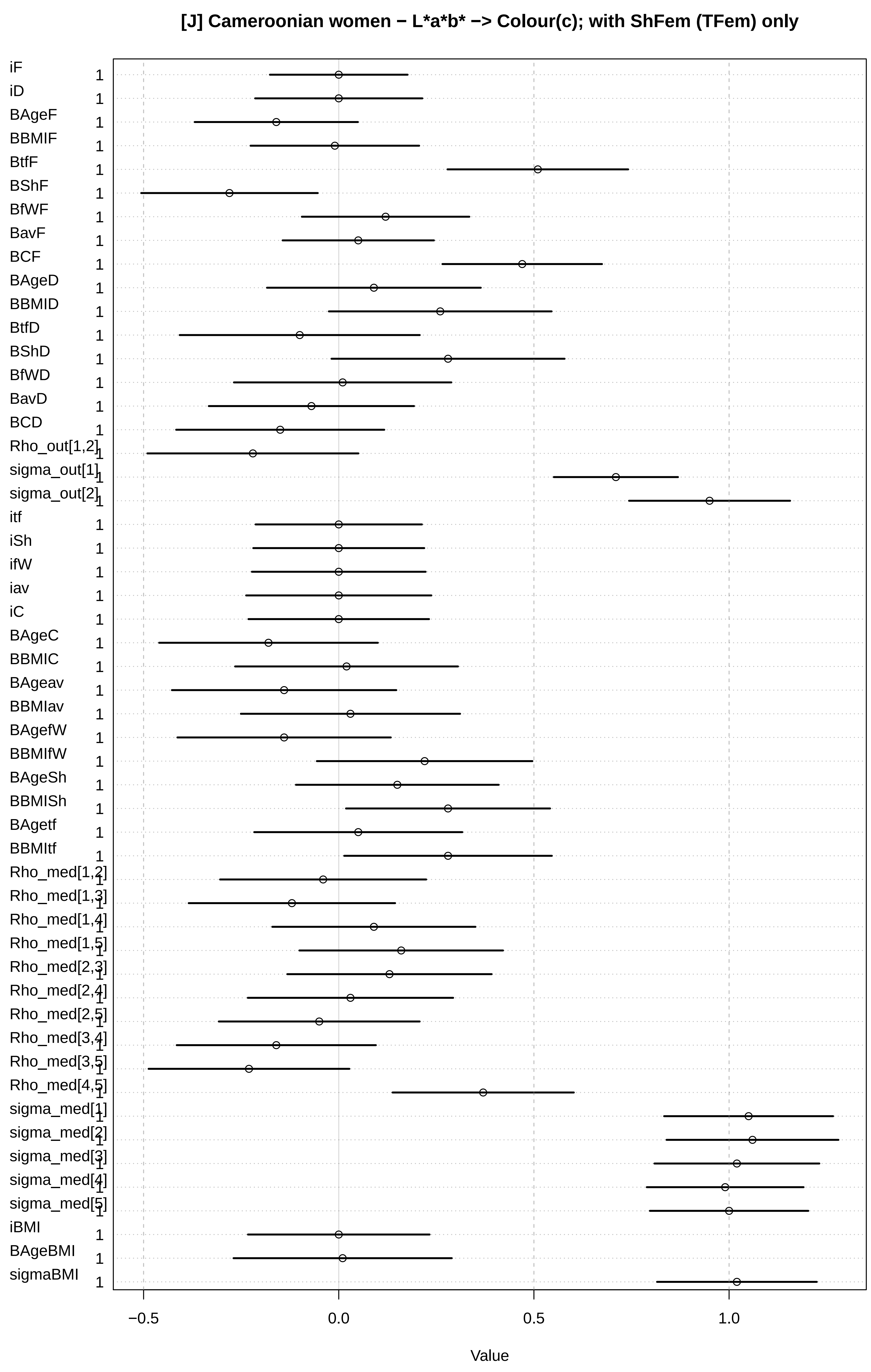
[J] Cameroonian women − L*a*b* −> Colour(c); with ShFem (TFem) only
iF
iD
BAgeF
BBMIF
BtfF
BShF
BfWF
BavF
BCF
BAgeD
BBMID
BtfD
BShD
BfWD
BavD
BCD
Rho_out[1,2]
sigma_out[1]
sigma_out[2]
itf
iSh
ifW
iav
iC
BAgeC
BBMIC
BAgeav
BBMIav
BAgefW
BBMIfW
BAgeSh
BBMISh
BAgetf
BBMItf
Rho_med[1,2]
Rho_med[1,3]
Rho_med[1,4]
Rho_med[1,5]
Rho_med[2,3]
Rho_med[2,4]
Rho_med[2,5]
Rho_med[3,4]
Rho_med[3,5]
Rho_med[4,5]
sigma_med[1]
sigma_med[2]
sigma_med[3]
sigma_med[4]
sigma_med[5]
iBMI
BAgeBMI
sigmaBMI
−0.5
0.0
0.5
1.0
Value

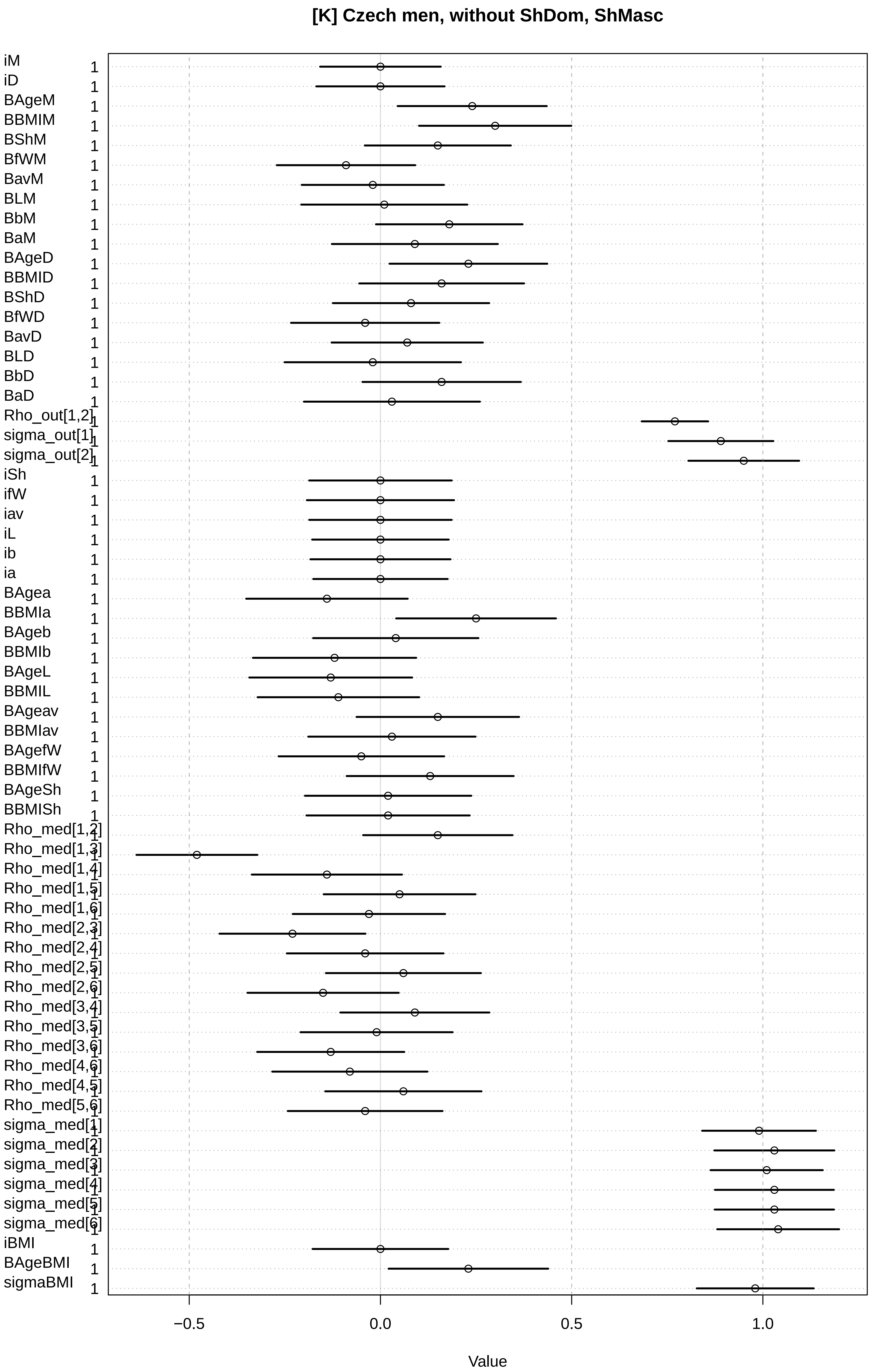
[K] Czech men, without ShDom, ShMasc
iM
iD
BAgeM
BBMIM
BShM
BfWM
BavM
BLM
BbM
BaM
BAgeD
BBMID
BShD
BfWD
BavD
BLD
BbD
BaD
Rho_out[1,2]
sigma_out[1]
sigma_out[2]
iSh
ifW
iav
iL
ib
ia
BAgea
BBMIa
BAgeb
BBMIb
BAgeL
BBMIL
BAgeav
BBMIav
BAgefW
BBMIfW
BAgeSh
BBMISh
Rho_med[1,2]
Rho_med[1,3]
Rho_med[1,4]
Rho_med[1,5]
Rho_med[1,6]
Rho_med[2,3]
Rho_med[2,4]
Rho_med[2,5]
Rho_med[2,6]
Rho_med[3,4]
Rho_med[3,5]
Rho_med[3,6]
Rho_med[4,6]
Rho_med[4,5]
Rho_med[5,6]
sigma_med[1]
sigma_med[2]
sigma_med[3]
sigma_med[4]
sigma_med[5]
sigma_med[6]
iBMI
BAgeBMI
sigmaBMI
−0.5
0.0
0.5
1.0
Value

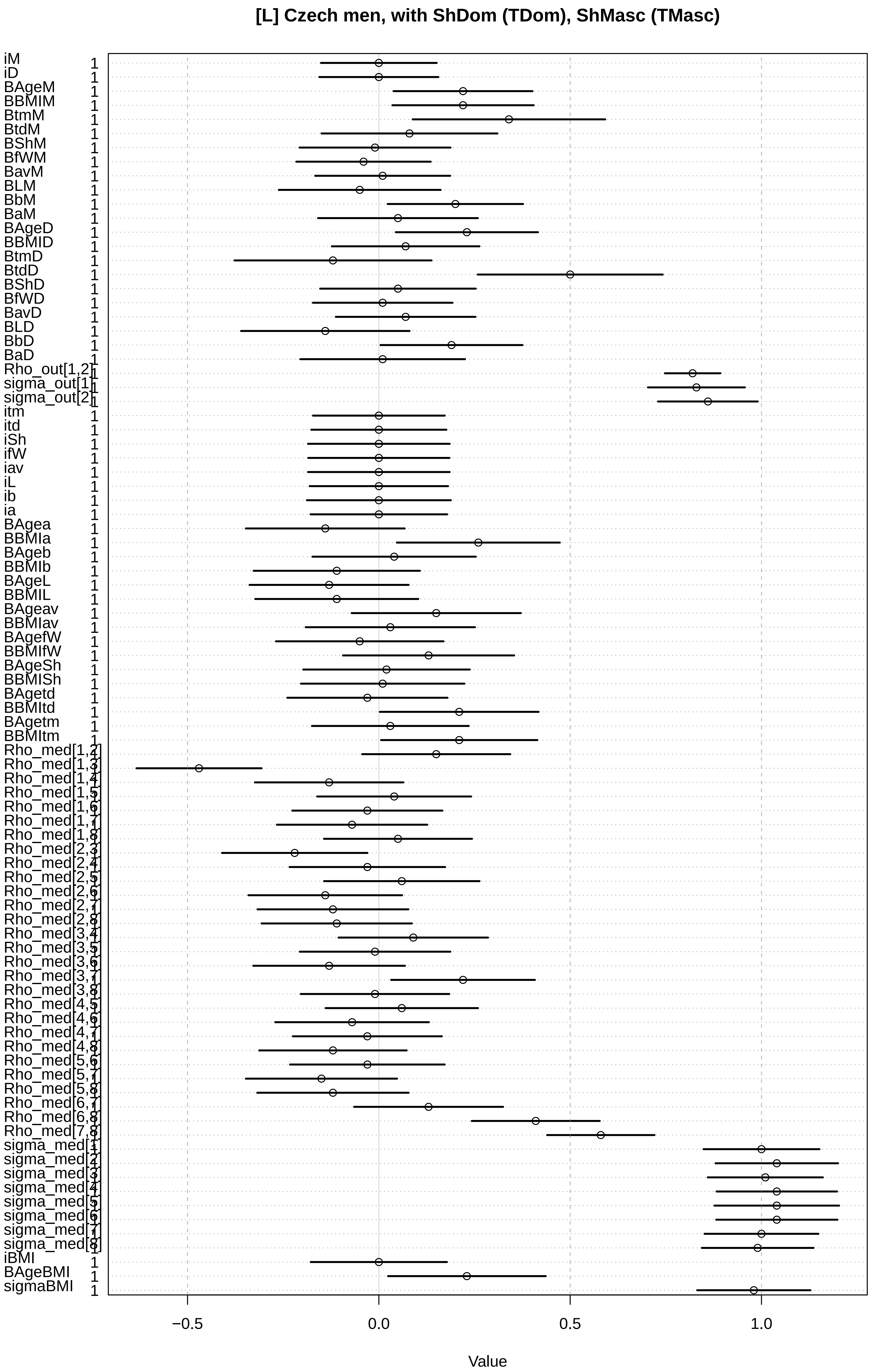
[L] Czech men, with ShDom (TDom), ShMasc (TMasc)
iM
iD
BAgeM
BBMIM
BtmM
BtdM
BShM
BfWM
BavM
BLM
BbM
BaM
BAgeD
BBMID
BtmD
BtdD
BShD
BfWD
BavD
BLD
BbD
BaD
Rho_out[1,2]
sigma_out[1]
sigma_out[2]
itm
itd
iSh
ifW
iav
iL
ib
ia
BAgea
BBMIa
BAgeb
BBMIb
BAgeL
BBMIL
BAgeav
BBMIav
BAgefW
BBMIfW
BAgeSh
BBMISh
BAgetd
BBMItd
BAgetm
BBMItm
Rho_med[1,2]
Rho_med[1,3]
Rho_med[1,4]
Rho_med[1,5]
Rho_med[1,6]
Rho_med[1,7]
Rho_med[1,8]
Rho_med[2,3]
Rho_med[2,4]
Rho_med[2,5]
Rho_med[2,6]
Rho_med[2,7]
Rho_med[2,8]
Rho_med[3,4]
Rho_med[3,5]
Rho_med[3,6]
Rho_med[3,7]
Rho_med[3,8]
Rho_med[4,5]
Rho_med[4,6]
Rho_med[4,7]
Rho_med[4,8]
Rho_med[5,6]
Rho_med[5,7]
Rho_med[5,8]
Rho_med[6,7]
Rho_med[6,8]
Rho_med[7,8]
sigma_med[1]
sigma_med[2]
sigma_med[3]
sigma_med[4]
sigma_med[5]
sigma_med[6]
sigma_med[7]
sigma_med[8]
iBMI
BAgeBMI
sigmaBMI
−0.5
0.0
0.5
1.0
Value

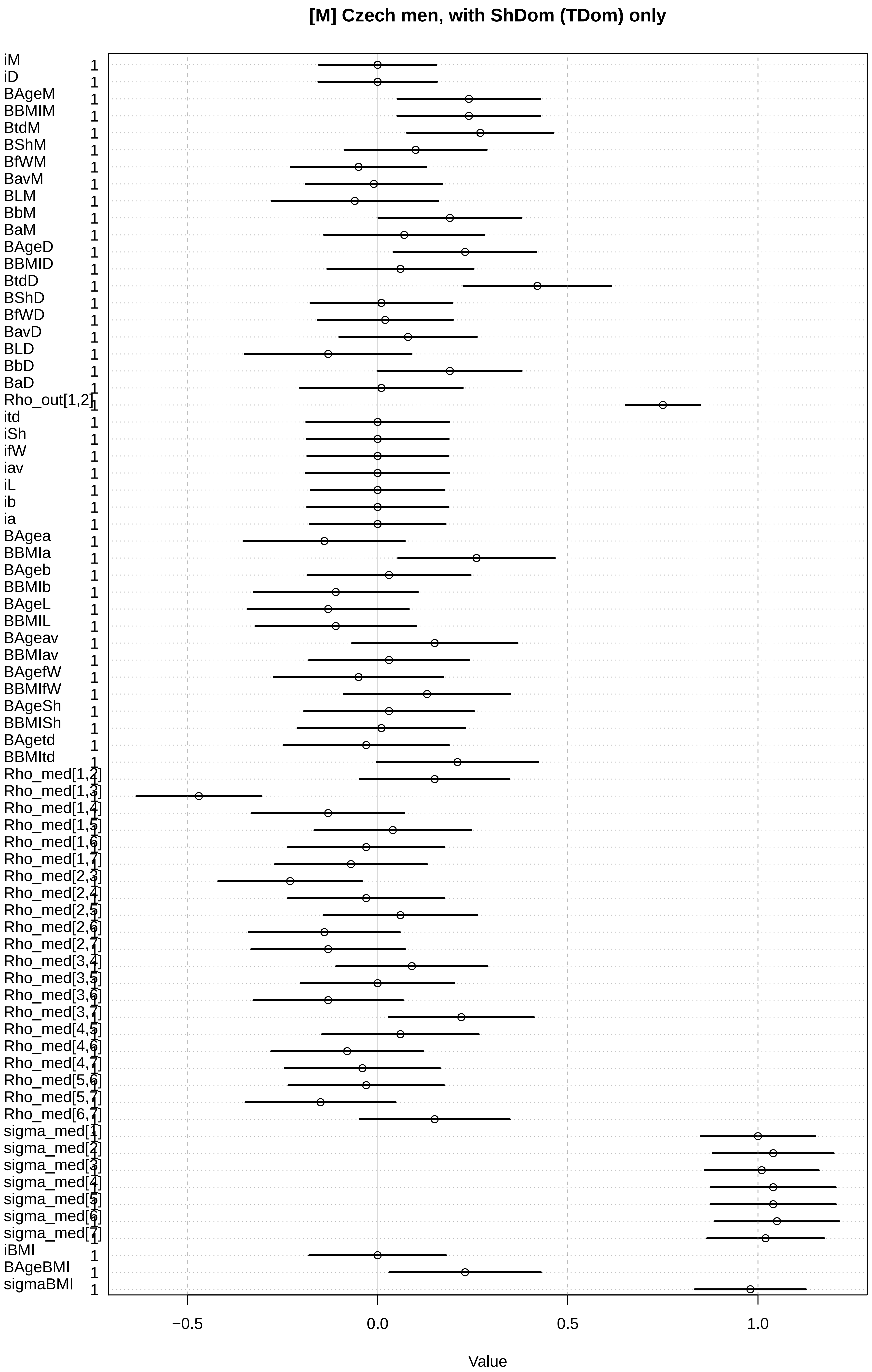

[M] Czech men, with ShDom (TDom) only
iM
iD
BAgeM
BBMIM
BtdM
BShM
BfWM
BavM
BLM
BbM
BaM
BAgeD
BBMID
BtdD
BShD
BfWD
BavD
BLD
BbD
BaD
Rho_out[1,2]
itd
iSh
ifW
iav
iL
ib
ia
BAgea
BBMIa
BAgeb
BBMIb
BAgeL
BBMIL
BAgeav
BBMIav
BAgefW
BBMIfW
BAgeSh
BBMISh
BAgetd
BBMItd
Rho_med[1,2]
Rho_med[1,3]
Rho_med[1,4]
Rho_med[1,5]
Rho_med[1,6]
Rho_med[1,7]
Rho_med[2,3]
Rho_med[2,4]
Rho_med[2,5]
Rho_med[2,6]
Rho_med[2,7]
Rho_med[3,4]
Rho_med[3,5]
Rho_med[3,6]
Rho_med[3,7]
Rho_med[4,5]
Rho_med[4,6]
Rho_med[4,7]
Rho_med[5,6]
Rho_med[5,7]
Rho_med[6,7]
sigma_med[1]
sigma_med[2]
sigma_med[3]
sigma_med[4]
sigma_med[5]
sigma_med[6]
sigma_med[7]
iBMI
BAgeBMI
sigmaBMI
−0.5
0.0
0.5
1.0
Value

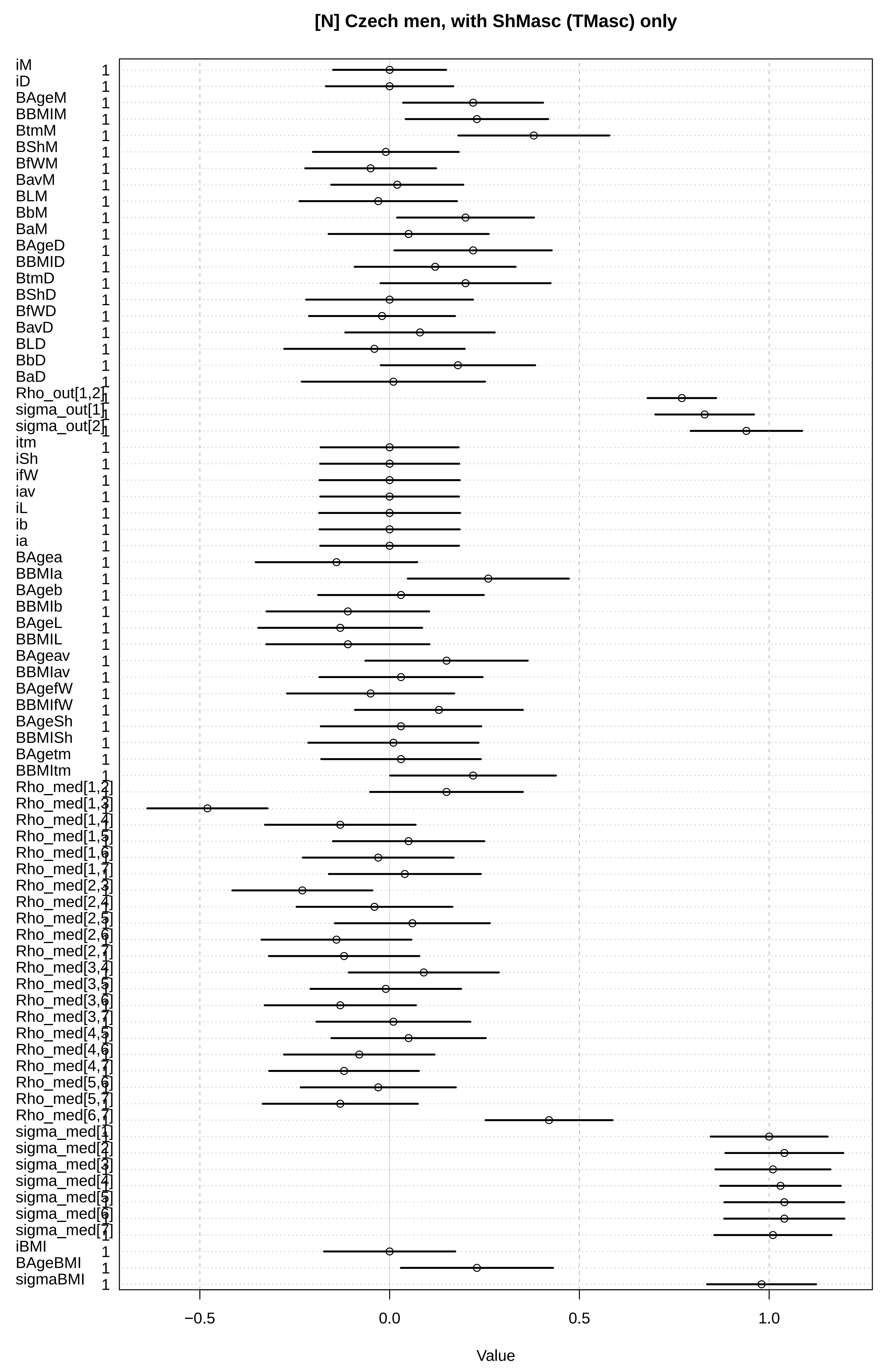

[N] Czech men, with ShMasc (TMasc) only
iM
iD
BAgeM
BBMIM
BtmM
BShM
BfWM
BavM
BLM
BbM
BaM
BAgeD
BBMID
BtmD
BShD
BfWD
BavD
BLD
BbD
BaD
Rho_out[1,2]
sigma_out[1]
sigma_out[2]
itm
iSh
ifW
iav
iL
ib
ia
BAgea
BBMIa
BAgeb
BBMIb
BAgeL
BBMIL
BAgeav
BBMIav
BAgefW
BBMIfW
BAgeSh
BBMISh
BAgetm
BBMItm
Rho_med[1,2]
Rho_med[1,3]
Rho_med[1,4]
Rho_med[1,5]
Rho_med[1,6]
Rho_med[1,7]
Rho_med[2,3]
Rho_med[2,4]
Rho_med[2,5]
Rho_med[2,6]
Rho_med[2,7]
Rho_med[3,4]
Rho_med[3,5]
Rho_med[3,6]
Rho_med[3,7]
Rho_med[4,5]
Rho_med[4,6]
Rho_med[4,7]
Rho_med[5,6]
Rho_med[5,7]
Rho_med[6,7]
sigma_med[1]
sigma_med[2]
sigma_med[3]
sigma_med[4]
sigma_med[5]
sigma_med[6]
sigma_med[7]
iBMI
BAgeBMI
sigmaBMI
−0.5
0.0
0.5
1.0
Value

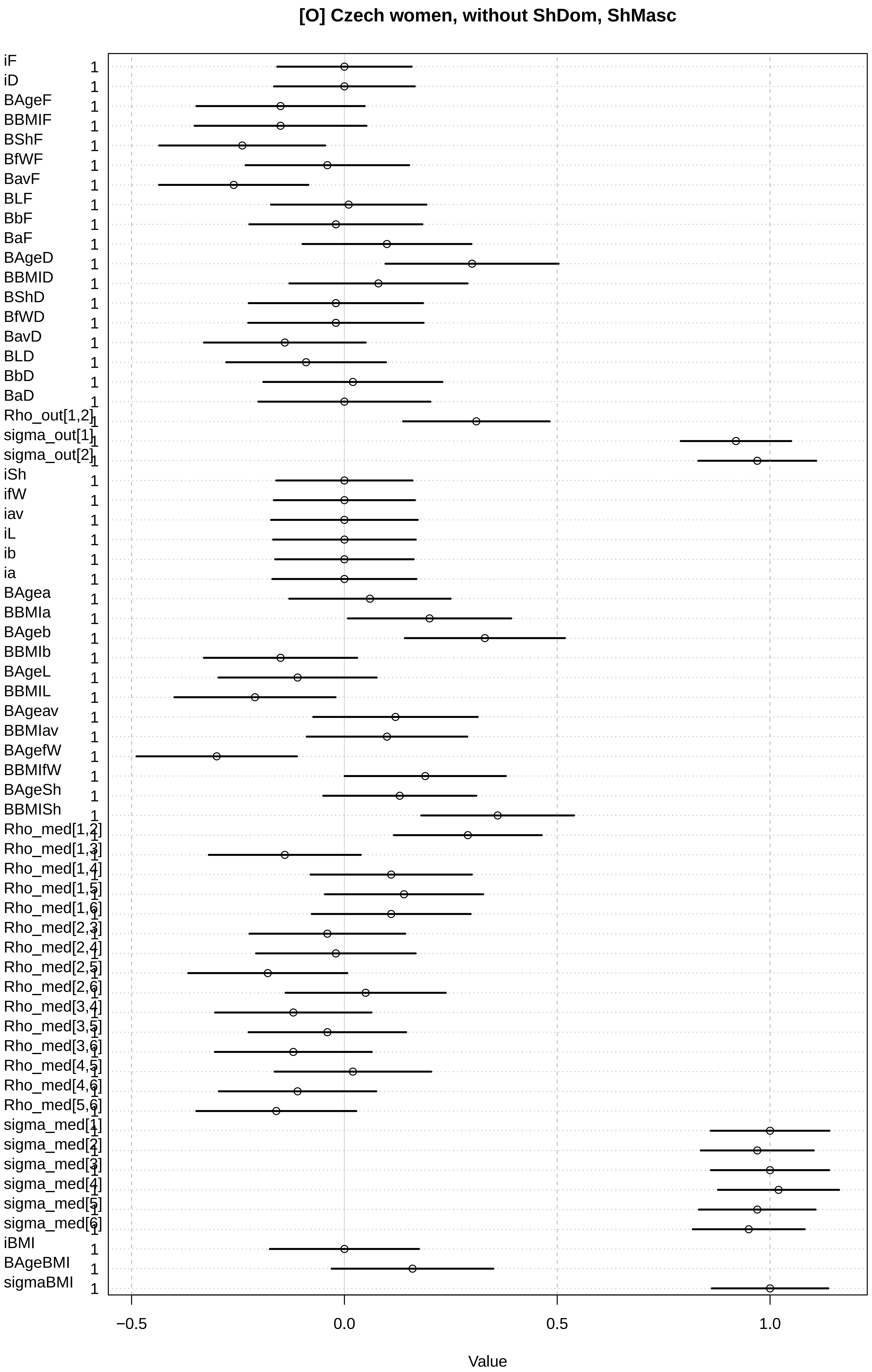

[O] Czech women, without ShDom, ShMasc
iF
iD
BAgeF
BBMIF
BShF
BfWF
BavF
BLF
BbF
BaF
BAgeD
BBMID
BShD
BfWD
BavD
BLD
BbD
BaD
Rho_out[1,2]
sigma_out[1]
sigma_out[2]
iSh
ifW
iav
iL
ib
ia
BAgea
BBMIa
BAgeb
BBMIb
BAgeL
BBMIL
BAgeav
BBMIav
BAgefW
BBMIfW
BAgeSh
BBMISh
Rho_med[1,2]
Rho_med[1,3]
Rho_med[1,4]
Rho_med[1,5]
Rho_med[1,6]
Rho_med[2,3]
Rho_med[2,4]
Rho_med[2,5]
Rho_med[2,6]
Rho_med[3,4]
Rho_med[3,5]
Rho_med[3,6]
Rho_med[4,5]
Rho_med[4,6]
Rho_med[5,6]
sigma_med[1]
sigma_med[2]
sigma_med[3]
sigma_med[4]
sigma_med[5]
sigma_med[6]
iBMI
BAgeBMI
sigmaBMI
−0.5
0.0
0.5
1.0
Value

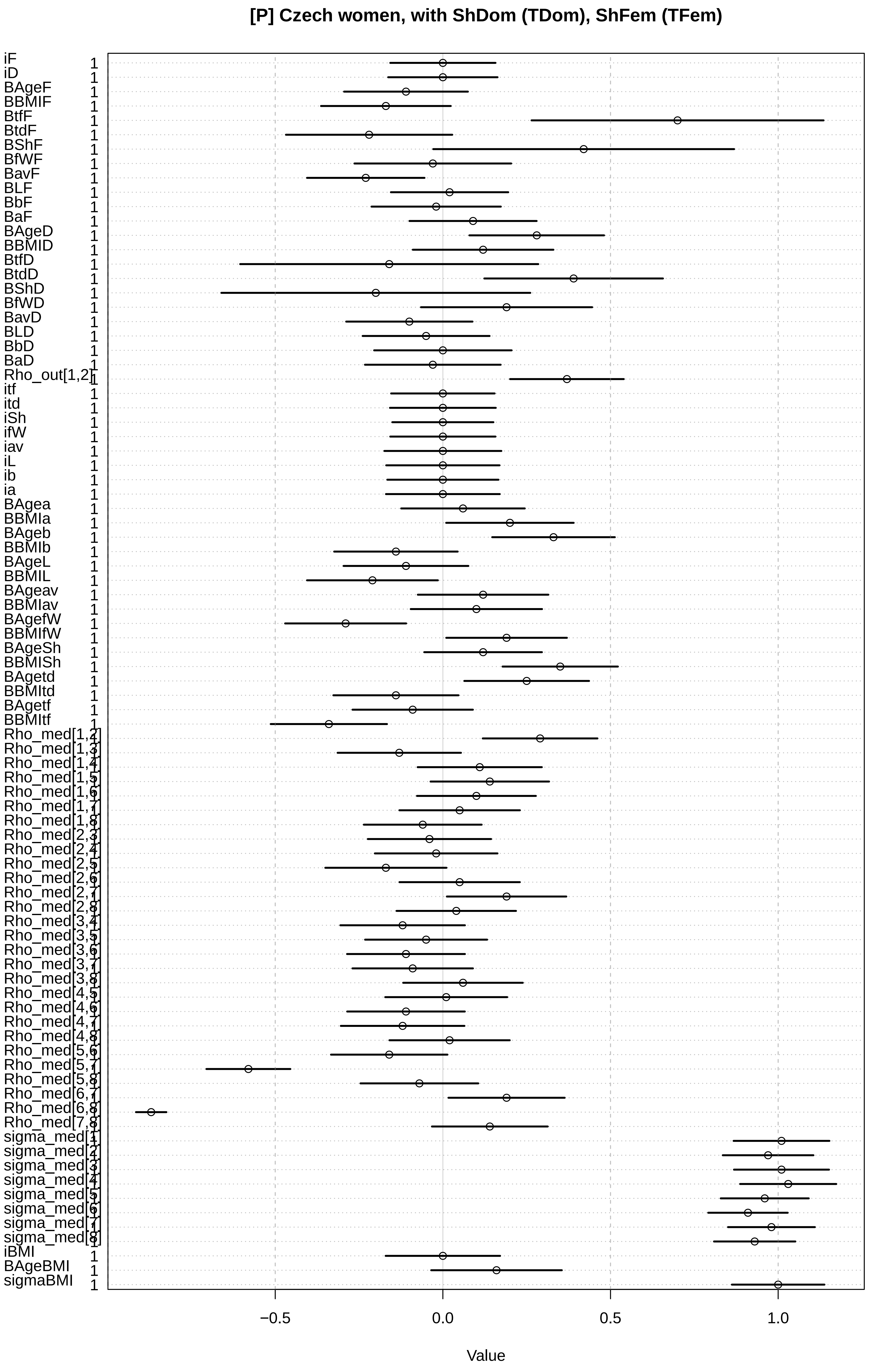

[P] Czech women, with ShDom (TDom), ShFem (TFem)
iF
iD
BAgeF
BBMIF
BtfF
BtdF
BShF
BfWF
BavF
BLF
BbF
BaF
BAgeD
BBMID
BtfD
BtdD
BShD
BfWD
BavD
BLD
BbD
BaD
Rho_out[1,2]
itf
itd
iSh
ifW
iav
iL
ib
ia
BAgea
BBMIa
BAgeb
BBMIb
BAgeL
BBMIL
BAgeav
BBMIav
BAgefW
BBMIfW
BAgeSh
BBMISh
BAgetd
BBMItd
BAgetf
BBMItf
Rho_med[1,2]
Rho_med[1,3]
Rho_med[1,4]
Rho_med[1,5]
Rho_med[1,6]
Rho_med[1,7]
Rho_med[1,8]
Rho_med[2,3]
Rho_med[2,4]
Rho_med[2,5]
Rho_med[2,6]
Rho_med[2,7]
Rho_med[2,8]
Rho_med[3,4]
Rho_med[3,5]
Rho_med[3,6]
Rho_med[3,7]
Rho_med[3,8]
Rho_med[4,5]
Rho_med[4,6]
Rho_med[4,7]
Rho_med[4,8]
Rho_med[5,6]
Rho_med[5,7]
Rho_med[5,8]
Rho_med[6,7]
Rho_med[6,8]
Rho_med[7,8]
sigma_med[1]
sigma_med[2]
sigma_med[3]
sigma_med[4]
sigma_med[5]
sigma_med[6]
sigma_med[7]
sigma_med[8]
iBMI
BAgeBMI
sigmaBMI
−0.5
0.0
0.5
1.0
Value

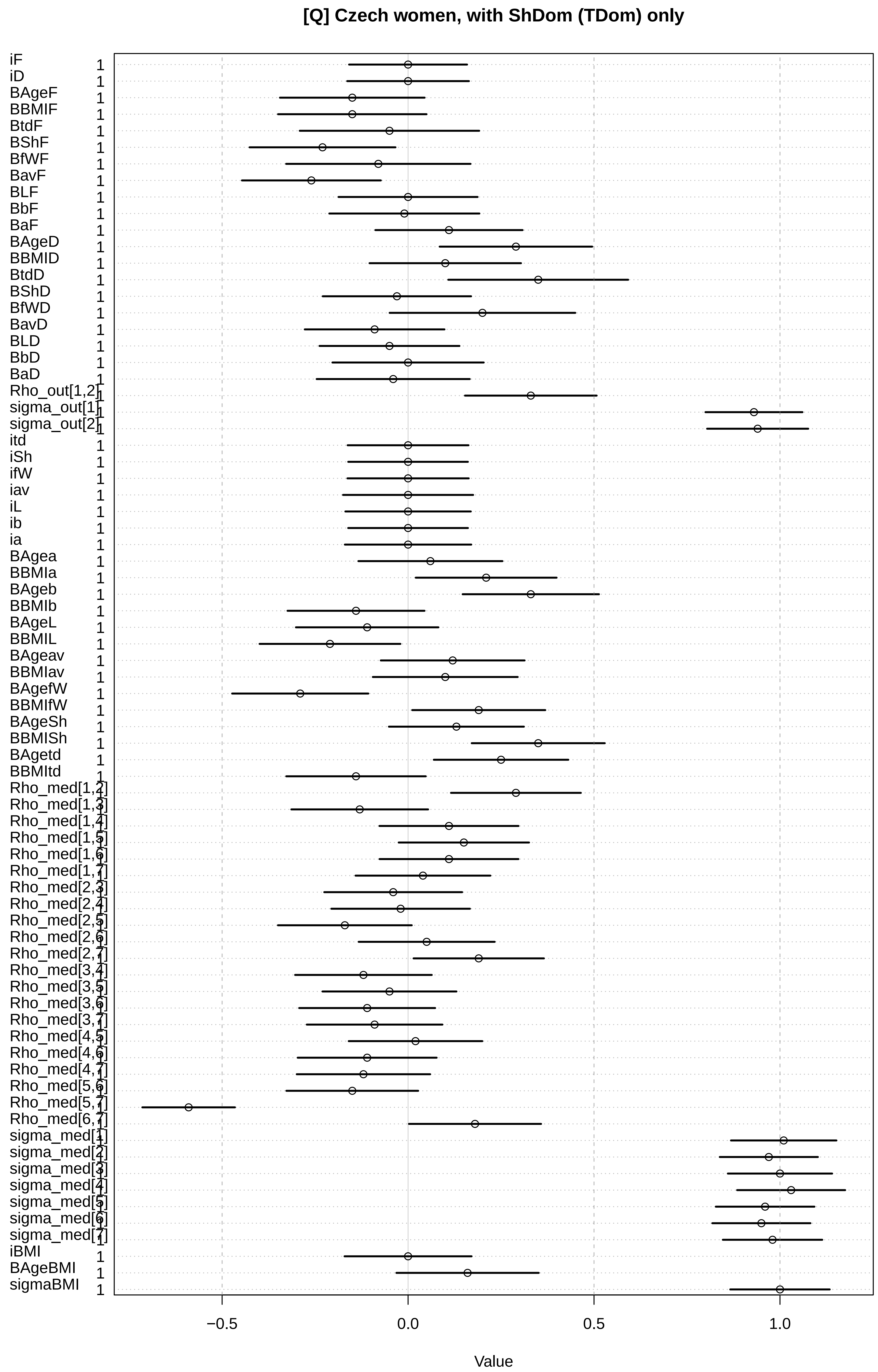
[Q] Czech women, with ShDom (TDom) only
iF
iD
BAgeF
BBMIF
BtdF
BShF
BfWF
BavF
BLF
BbF
BaF
BAgeD
BBMID
BtdD
BShD
BfWD
BavD
BLD
BbD
BaD
Rho_out[1,2]
sigma_out[1]
sigma_out[2]
itd
iSh
ifW
iav
iL
ib
ia
BAgea
BBMIa
BAgeb
BBMIb
BAgeL
BBMIL
BAgeav
BBMIav
BAgefW
BBMIfW
BAgeSh
BBMISh
BAgetd
BBMItd
Rho_med[1,2]
Rho_med[1,3]
Rho_med[1,4]
Rho_med[1,5]
Rho_med[1,6]
Rho_med[1,7]
Rho_med[2,3]
Rho_med[2,4]
Rho_med[2,5]
Rho_med[2,6]
Rho_med[2,7]
Rho_med[3,4]
Rho_med[3,5]
Rho_med[3,6]
Rho_med[3,7]
Rho_med[4,5]
Rho_med[4,6]
Rho_med[4,7]
Rho_med[5,6]
Rho_med[5,7]
Rho_med[6,7]
sigma_med[1]
sigma_med[2]
sigma_med[3]
sigma_med[4]
sigma_med[5]
sigma_med[6]
sigma_med[7]
iBMI
BAgeBMI
sigmaBMI
−0.5
0.0
0.5
1.0
Value

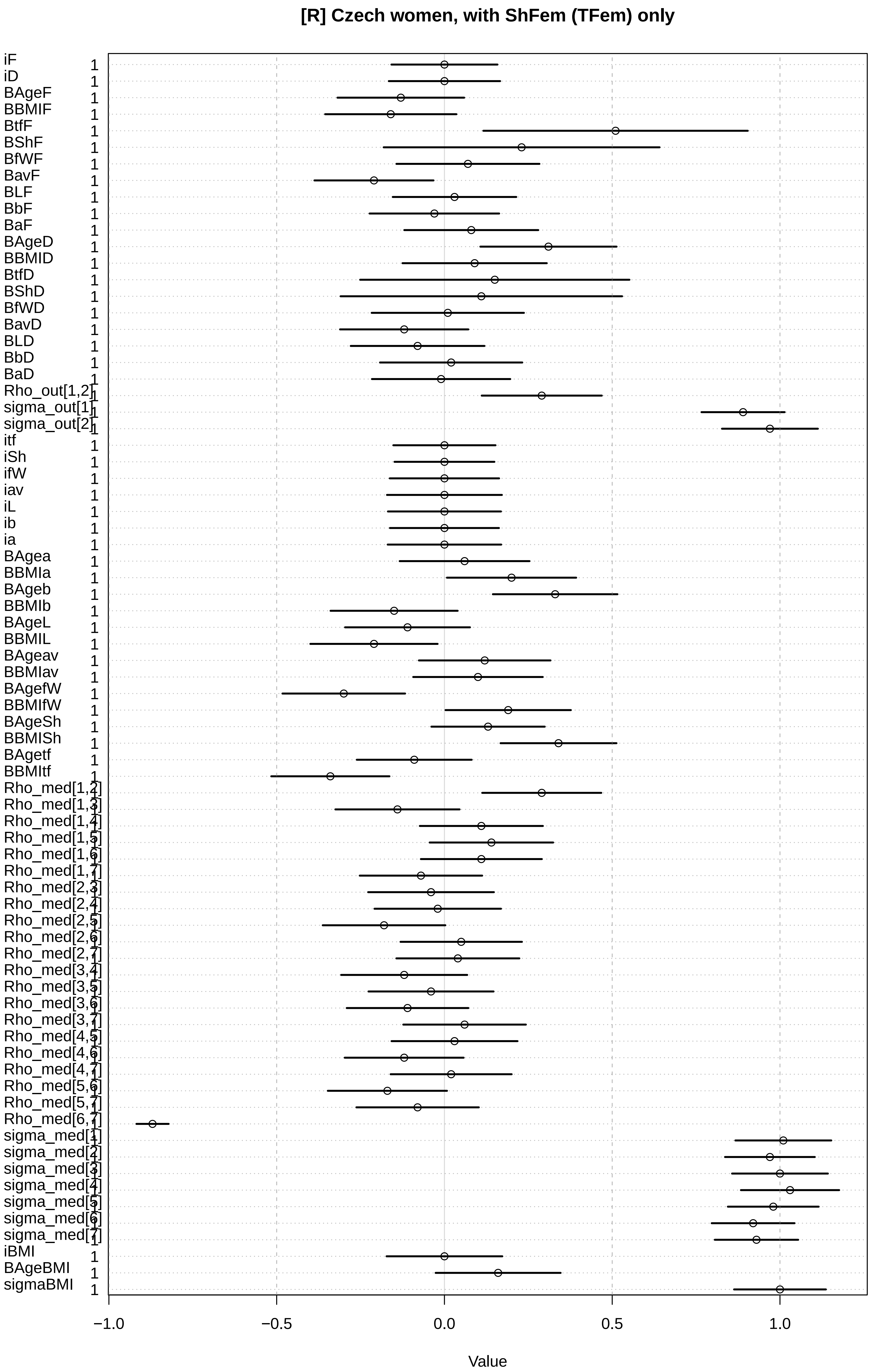

[R] Czech women, with ShFem (TFem) only
iF
iD
BAgeF
BBMIF
BtfF
BShF
BfWF
BavF
BLF
BbF
BaF
BAgeD
BBMID
BtfD
BShD
BfWD
BavD
BLD
BbD
BaD
Rho_out[1,2]
sigma_out[1]
sigma_out[2]
itf
iSh
ifW
iav
iL
ib
ia
BAgea
BBMIa
BAgeb
BBMIb
BAgeL
BBMIL
BAgeav
BBMIav
BAgefW
BBMIfW
BAgeSh
BBMISh
BAgetf
BBMItf
Rho_med[1,2]
Rho_med[1,3]
Rho_med[1,4]
Rho_med[1,5]
Rho_med[1,6]
Rho_med[1,7]
Rho_med[2,3]
Rho_med[2,4]
Rho_med[2,5]
Rho_med[2,6]
Rho_med[2,7]
Rho_med[3,4]
Rho_med[3,5]
Rho_med[3,6]
Rho_med[3,7]
Rho_med[4,5]
Rho_med[4,6]
Rho_med[4,7]
Rho_med[5,6]
Rho_med[5,7]
Rho_med[6,7]
sigma_med[1]
sigma_med[2]
sigma_med[3]
sigma_med[4]
sigma_med[5]
sigma_med[6]
sigma_med[7]
iBMI
BAgeBMI
sigmaBMI
−1.0
−0.5
0.0
0.5
1.0
Value

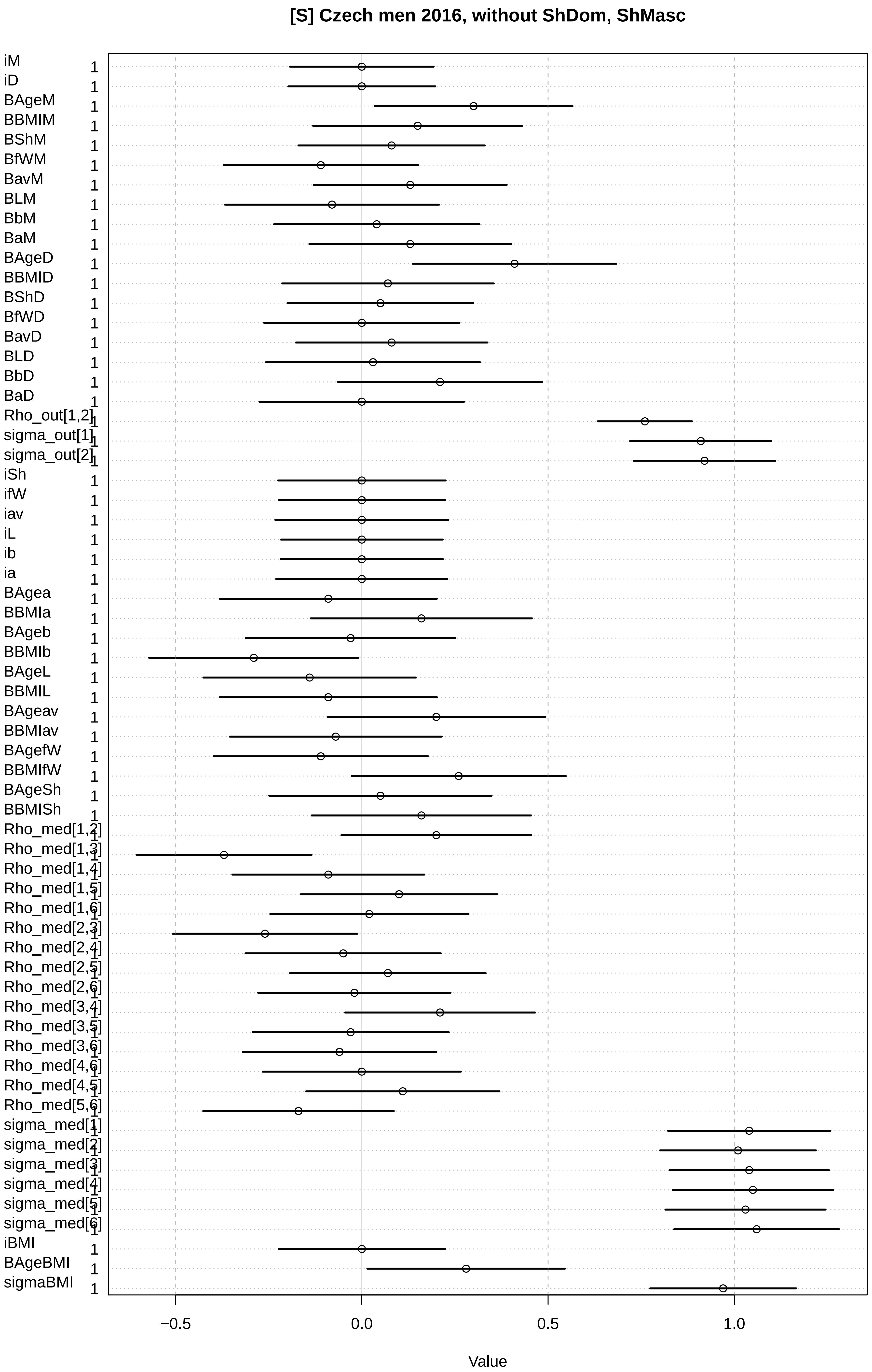
[S] Czech men 2016, without ShDom, ShMasc
iM
iD
BAgeM
BBMIM
BShM
BfWM
BavM
BLM
BbM
BaM
BAgeD
BBMID
BShD
BfWD
BavD
BLD
BbD
BaD
Rho_out[1,2]
sigma_out[1]
sigma_out[2]
iSh
ifW
iav
iL
ib
ia
BAgea
BBMIa
BAgeb
BBMIb
BAgeL
BBMIL
BAgeav
BBMIav
BAgefW
BBMIfW
BAgeSh
BBMISh
Rho_med[1,2]
Rho_med[1,3]
Rho_med[1,4]
Rho_med[1,5]
Rho_med[1,6]
Rho_med[2,3]
Rho_med[2,4]
Rho_med[2,5]
Rho_med[2,6]
Rho_med[3,4]
Rho_med[3,5]
Rho_med[3,6]
Rho_med[4,6]
Rho_med[4,5]
Rho_med[5,6]
sigma_med[1]
sigma_med[2]
sigma_med[3]
sigma_med[4]
sigma_med[5]
sigma_med[6]
iBMI
BAgeBMI
sigmaBMI
−0.5
0.0
0.5
1.0
Value

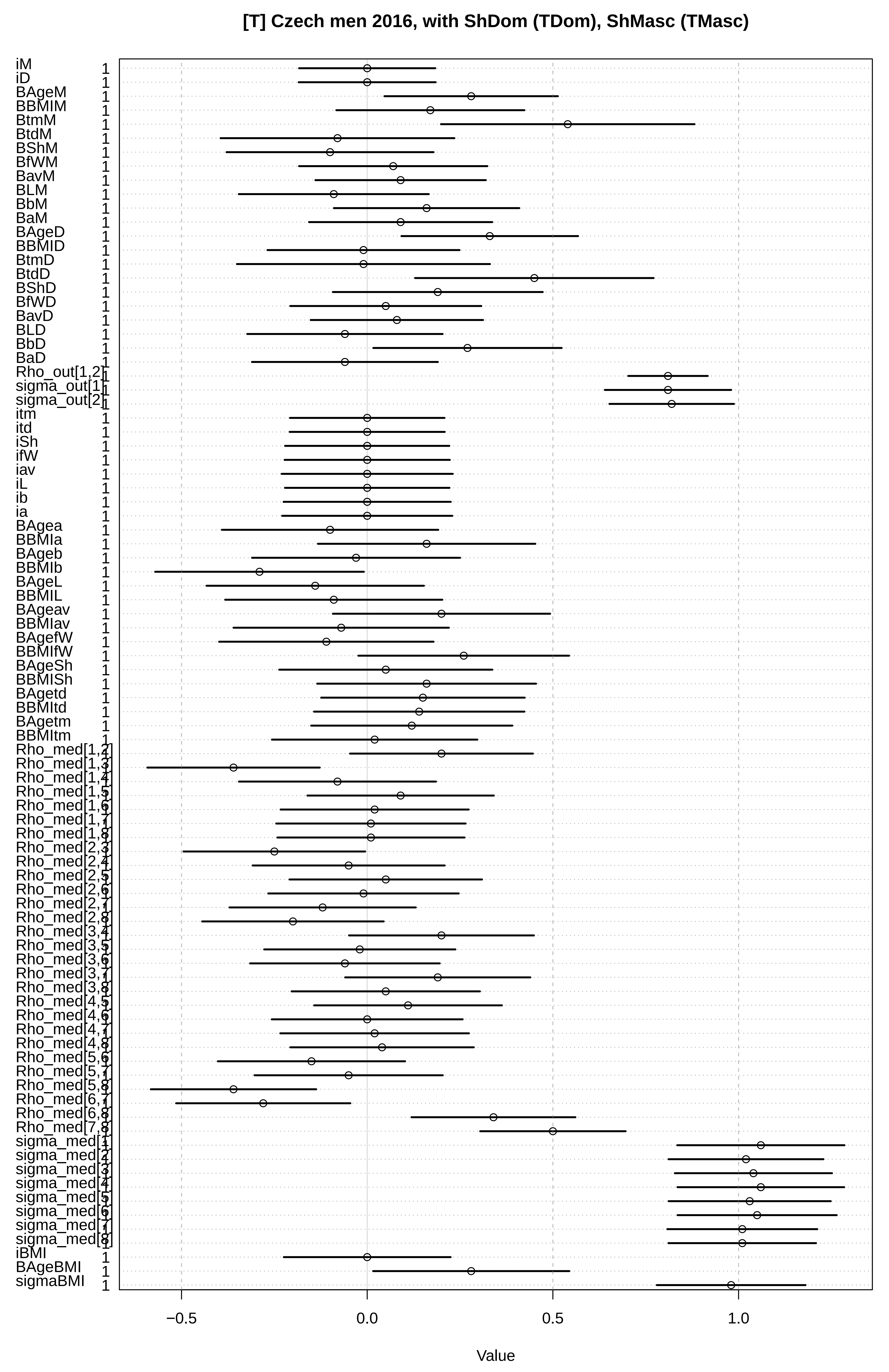

[T] Czech men 2016, with ShDom (TDom), ShMasc (TMasc)
iM
iD
BAgeM
BBMIM
BtmM
BtdM
BShM
BfWM
BavM
BLM
BbM
BaM
BAgeD
BBMID
BtmD
BtdD
BShD
BfWD
BavD
BLD
BbD
BaD
Rho_out[1,2]
sigma_out[1]
sigma_out[2]
itm
itd
iSh
ifW
iav
iL
ib
ia
BAgea
BBMIa
BAgeb
BBMIb
BAgeL
BBMIL
BAgeav
BBMIav
BAgefW
BBMIfW
BAgeSh
BBMISh
BAgetd
BBMItd
BAgetm
BBMItm
Rho_med[1,2]
Rho_med[1,3]
Rho_med[1,4]
Rho_med[1,5]
Rho_med[1,6]
Rho_med[1,7]
Rho_med[1,8]
Rho_med[2,3]
Rho_med[2,4]
Rho_med[2,5]
Rho_med[2,6]
Rho_med[2,7]
Rho_med[2,8]
Rho_med[3,4]
Rho_med[3,5]
Rho_med[3,6]
Rho_med[3,7]
Rho_med[3,8]
Rho_med[4,5]
Rho_med[4,6]
Rho_med[4,7]
Rho_med[4,8]
Rho_med[5,6]
Rho_med[5,7]
Rho_med[5,8]
Rho_med[6,7]
Rho_med[6,8]
Rho_med[7,8]
sigma_med[1]
sigma_med[2]
sigma_med[3]
sigma_med[4]
sigma_med[5]
sigma_med[6]
sigma_med[7]
sigma_med[8]
iBMI
BAgeBMI
sigmaBMI
−0.5
0.0
0.5
1.0
Value

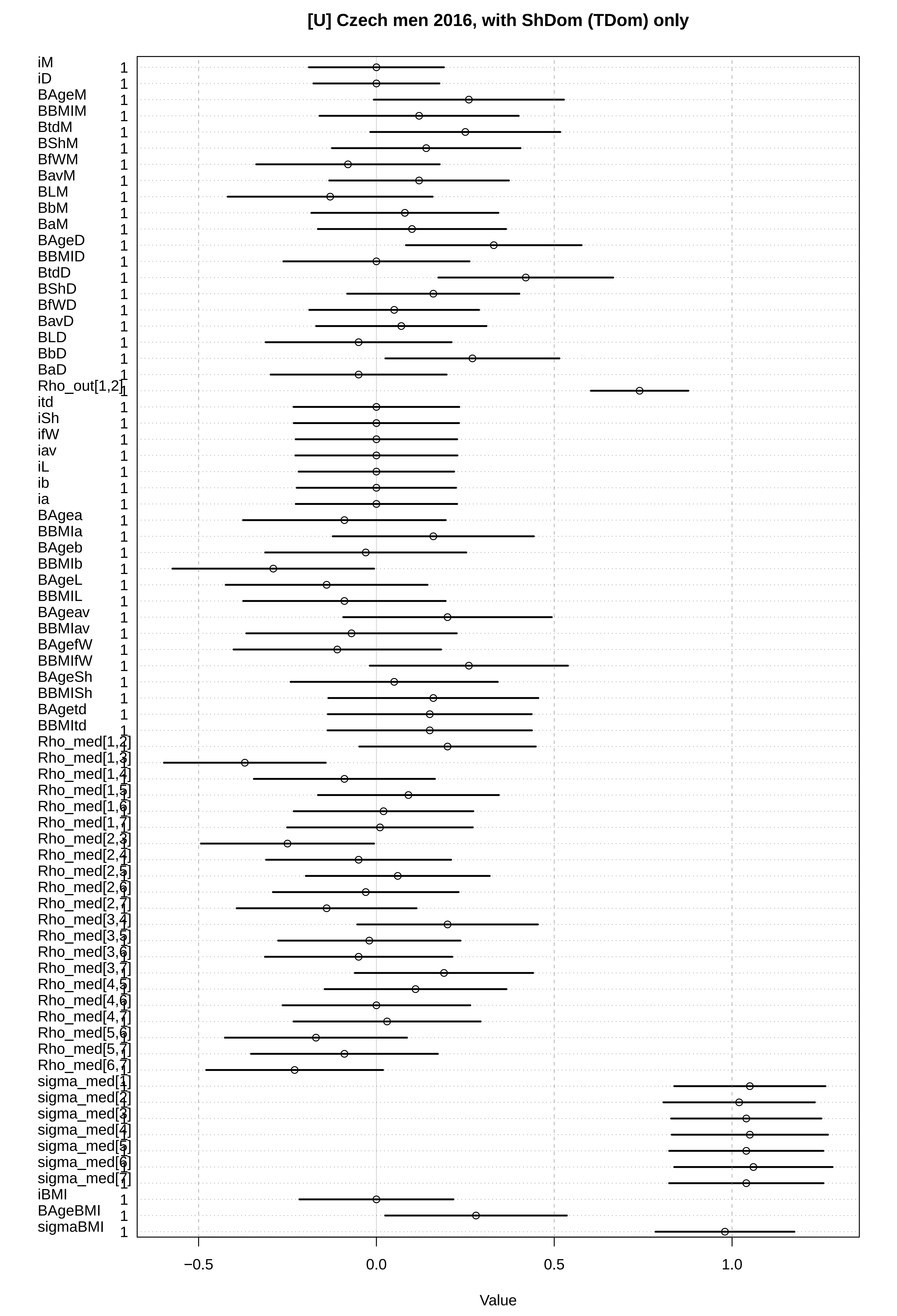
[U] Czech men 2016, with ShDom (TDom) only
iM
iD
BAgeM
BBMIM
BtdM
BShM
BfWM
BavM
BLM
BbM
BaM
BAgeD
BBMID
BtdD
BShD
BfWD
BavD
BLD
BbD
BaD
Rho_out[1,2]
itd
iSh
ifW
iav
iL
ib
ia
BAgea
BBMIa
BAgeb
BBMIb
BAgeL
BBMIL
BAgeav
BBMIav
BAgefW
BBMIfW
BAgeSh
BBMISh
BAgetd
BBMItd
Rho_med[1,2]
Rho_med[1,3]
Rho_med[1,4]
Rho_med[1,5]
Rho_med[1,6]
Rho_med[1,7]
Rho_med[2,3]
Rho_med[2,4]
Rho_med[2,5]
Rho_med[2,6]
Rho_med[2,7]
Rho_med[3,4]
Rho_med[3,5]
Rho_med[3,6]
Rho_med[3,7]
Rho_med[4,5]
Rho_med[4,6]
Rho_med[4,7]
Rho_med[5,6]
Rho_med[5,7]
Rho_med[6,7]
sigma_med[1]
sigma_med[2]
sigma_med[3]
sigma_med[4]
sigma_med[5]
sigma_med[6]
sigma_med[7]
iBMI
BAgeBMI
sigmaBMI
−0.5
0.0
0.5
1.0
Value

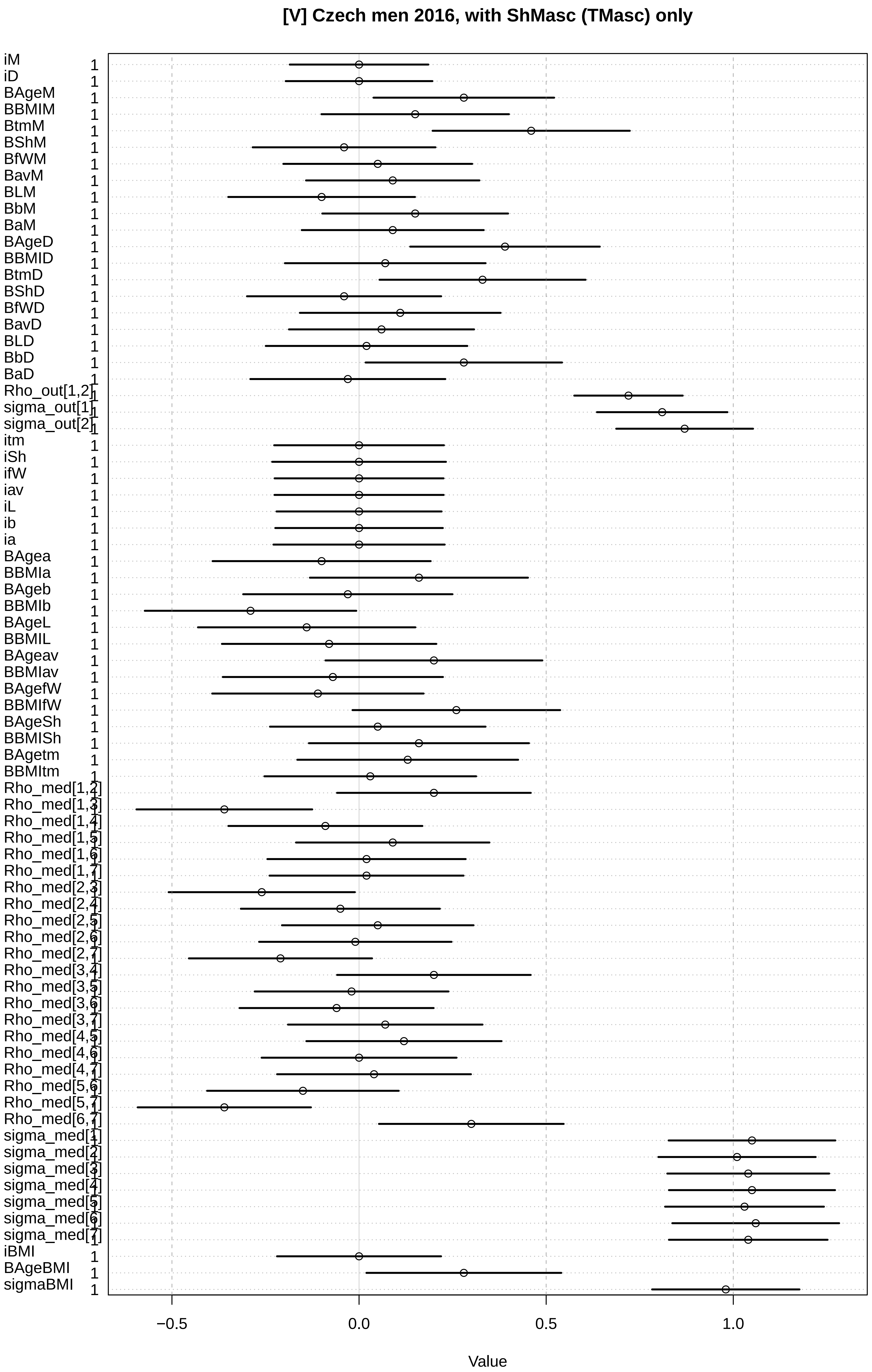
[V] Czech men 2016, with ShMasc (TMasc) only
iM
iD
BAgeM
BBMIM
BtmM
BShM
BfWM
BavM
BLM
BbM
BaM
BAgeD
BBMID
BtmD
BShD
BfWD
BavD
BLD
BbD
BaD
Rho_out[1,2]
sigma_out[1]
sigma_out[2]
itm
iSh
ifW
iav
iL
ib
ia
BAgea
BBMIa
BAgeb
BBMIb
BAgeL
BBMIL
BAgeav
BBMIav
BAgefW
BBMIfW
BAgeSh
BBMISh
BAgetm
BBMItm
Rho_med[1,2]
Rho_med[1,3]
Rho_med[1,4]
Rho_med[1,5]
Rho_med[1,6]
Rho_med[1,7]
Rho_med[2,3]
Rho_med[2,4]
Rho_med[2,5]
Rho_med[2,6]
Rho_med[2,7]
Rho_med[3,4]
Rho_med[3,5]
Rho_med[3,6]
Rho_med[3,7]
Rho_med[4,5]
Rho_med[4,6]
Rho_med[4,7]
Rho_med[5,6]
Rho_med[5,7]
Rho_med[6,7]
sigma_med[1]
sigma_med[2]
sigma_med[3]
sigma_med[4]
sigma_med[5]
sigma_med[6]
sigma_med[7]
iBMI
BAgeBMI
sigmaBMI
−0.5
0.0
0.5
1.0
Value

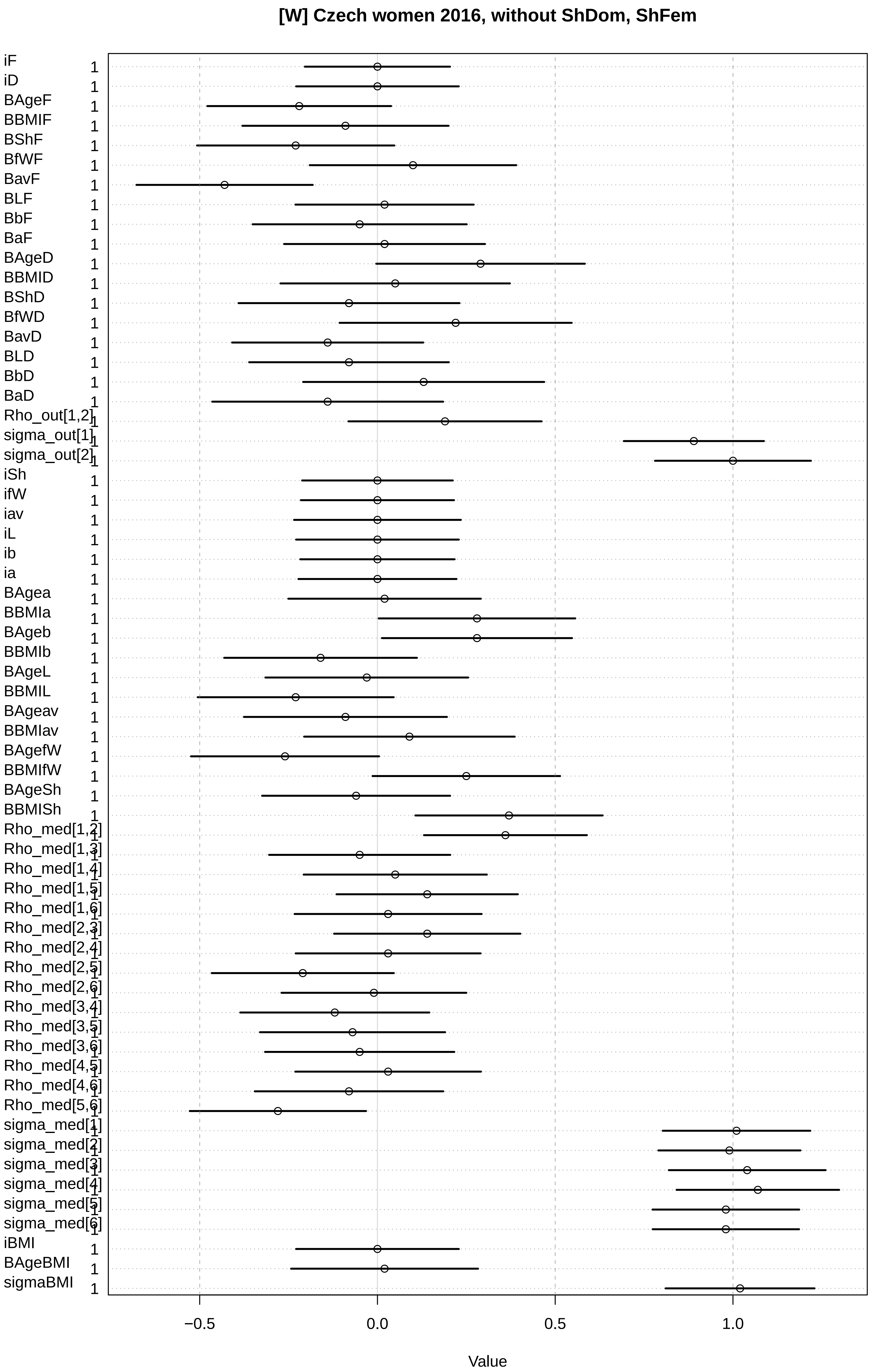
[W] Czech women 2016, without ShDom, ShFem
iF
iD
BAgeF
BBMIF
BShF
BfWF
BavF
BLF
BbF
BaF
BAgeD
BBMID
BShD
BfWD
BavD
BLD
BbD
BaD
Rho_out[1,2]
sigma_out[1]
sigma_out[2]
iSh
ifW
iav
iL
ib
ia
BAgea
BBMIa
BAgeb
BBMIb
BAgeL
BBMIL
BAgeav
BBMIav
BAgefW
BBMIfW
BAgeSh
BBMISh
Rho_med[1,2]
Rho_med[1,3]
Rho_med[1,4]
Rho_med[1,5]
Rho_med[1,6]
Rho_med[2,3]
Rho_med[2,4]
Rho_med[2,5]
Rho_med[2,6]
Rho_med[3,4]
Rho_med[3,5]
Rho_med[3,6]
Rho_med[4,5]
Rho_med[4,6]
Rho_med[5,6]
sigma_med[1]
sigma_med[2]
sigma_med[3]
sigma_med[4]
sigma_med[5]
sigma_med[6]
iBMI
BAgeBMI
sigmaBMI
−0.5
0.0
0.5
1.0
Value

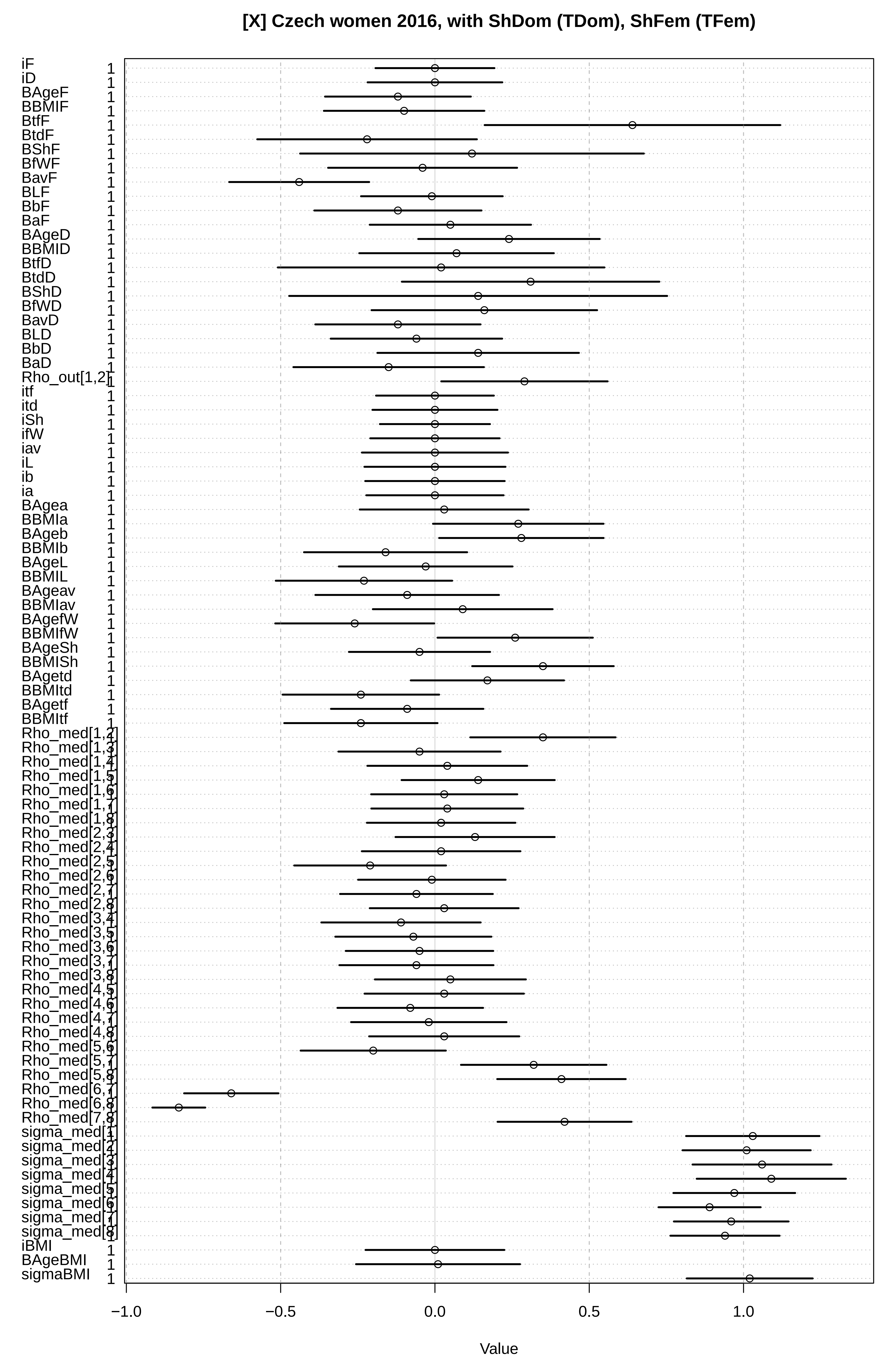

[X] Czech women 2016, with ShDom (TDom), ShFem (TFem)
iF
iD
BAgeF
BBMIF
BtfF
BtdF
BShF
BfWF
BavF
BLF
BbF
BaF
BAgeD
BBMID
BtfD
BtdD
BShD
BfWD
BavD
BLD
BbD
BaD
Rho_out[1,2]
itf
itd
iSh
ifW
iav
iL
ib
ia
BAgea
BBMIa
BAgeb
BBMIb
BAgeL
BBMIL
BAgeav
BBMIav
BAgefW
BBMIfW
BAgeSh
BBMISh
BAgetd
BBMItd
BAgetf
BBMItf
Rho_med[1,2]
Rho_med[1,3]
Rho_med[1,4]
Rho_med[1,5]
Rho_med[1,6]
Rho_med[1,7]
Rho_med[1,8]
Rho_med[2,3]
Rho_med[2,4]
Rho_med[2,5]
Rho_med[2,6]
Rho_med[2,7]
Rho_med[2,8]
Rho_med[3,4]
Rho_med[3,5]
Rho_med[3,6]
Rho_med[3,7]
Rho_med[3,8]
Rho_med[4,5]
Rho_med[4,6]
Rho_med[4,7]
Rho_med[4,8]
Rho_med[5,6]
Rho_med[5,7]
Rho_med[5,8]
Rho_med[6,7]
Rho_med[6,8]
Rho_med[7,8]
sigma_med[1]
sigma_med[2]
sigma_med[3]
sigma_med[4]
sigma_med[5]
sigma_med[6]
sigma_med[7]
sigma_med[8]
iBMI
BAgeBMI
sigmaBMI
−1.0
−0.5
0.0
0.5
1.0
Value

[Y] Czech women 2016, with ShDom (TDom) only

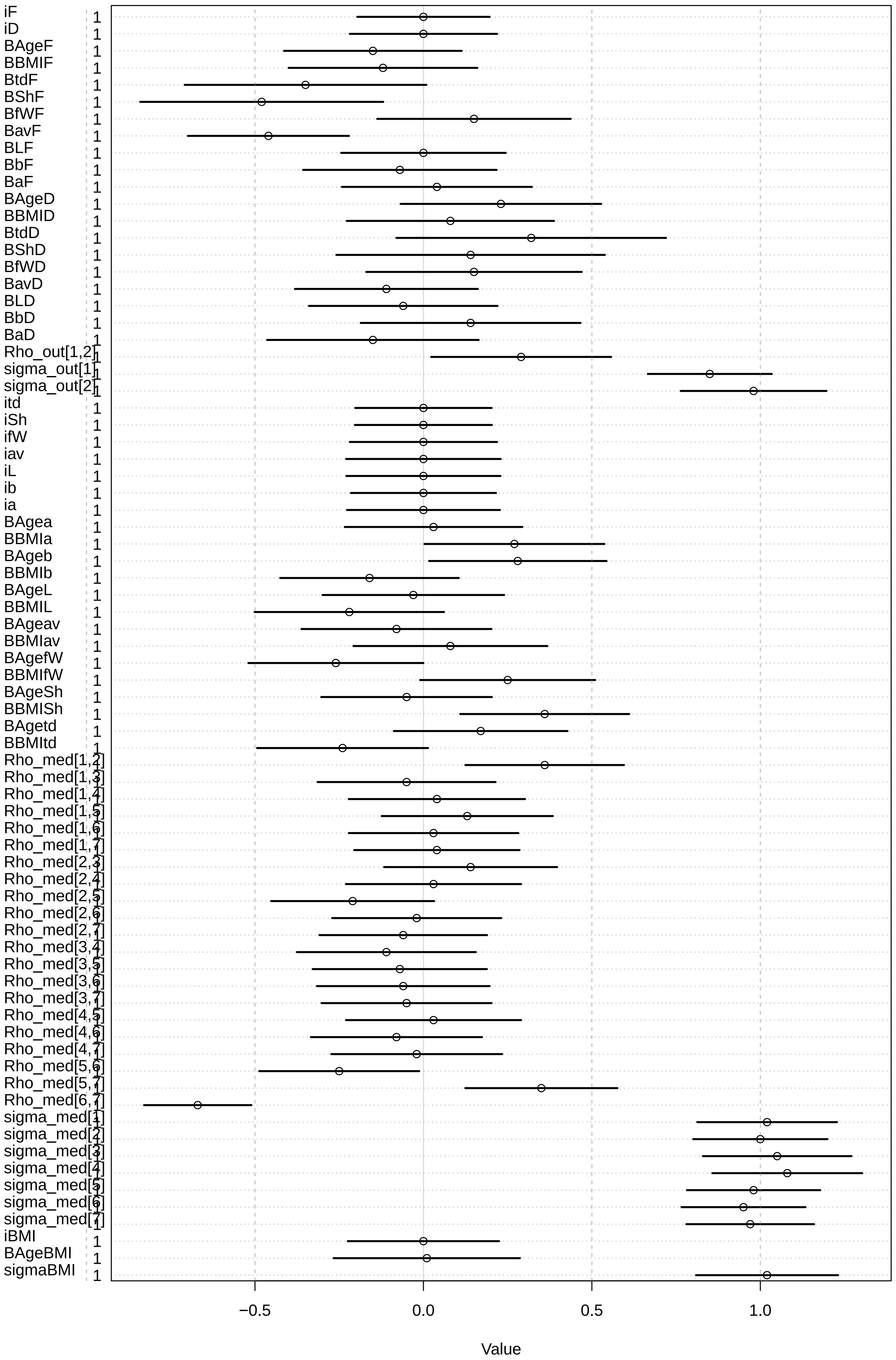

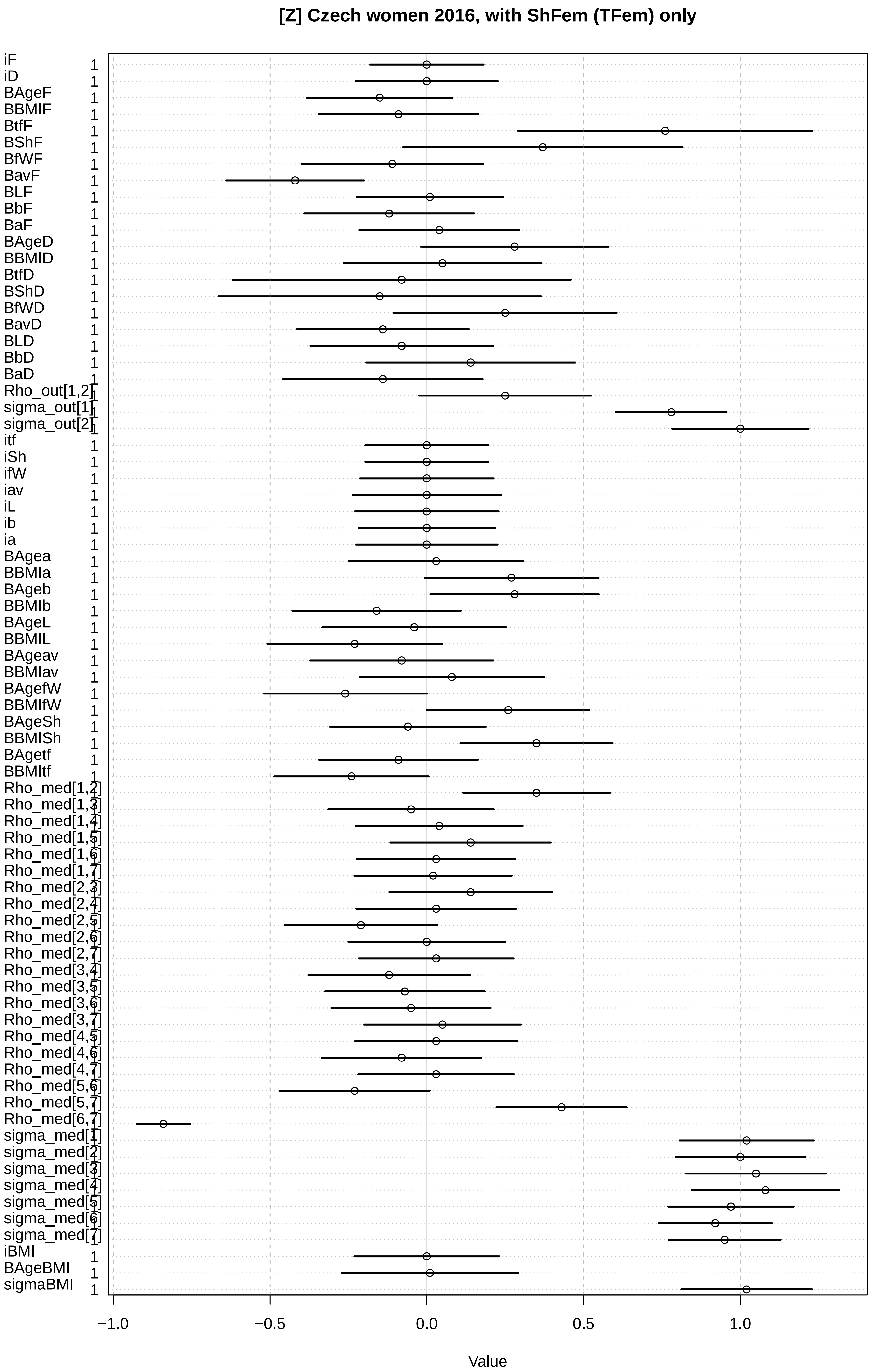
[Z] Czech women 2016, with ShFem (TFem) only
iF
iD
BAgeF
BBMIF
BtfF
BShF
BfWF
BavF
BLF
BbF
BaF
BAgeD
BBMID
BtfD
BShD
BfWD
BavD
BLD
BbD
BaD
Rho_out[1,2]
sigma_out[1]
sigma_out[2]
itf
iSh
ifW
iav
iL
ib
ia
BAgea
BBMIa
BAgeb
BBMIb
BAgeL
BBMIL
BAgeav
BBMIav
BAgefW
BBMIfW
BAgeSh
BBMISh
BAgetf
BBMItf
Rho_med[1,2]
Rho_med[1,3]
Rho_med[1,4]
Rho_med[1,5]
Rho_med[1,6]
Rho_med[1,7]
Rho_med[2,3]
Rho_med[2,4]
Rho_med[2,5]
Rho_med[2,6]
Rho_med[2,7]
Rho_med[3,4]
Rho_med[3,5]
Rho_med[3,6]
Rho_med[3,7]
Rho_med[4,5]
Rho_med[4,6]
Rho_med[4,7]
Rho_med[5,6]
Rho_med[5,7]
Rho_med[6,7]
sigma_med[1]
sigma_med[2]
sigma_med[3]
sigma_med[4]
sigma_med[5]
sigma_med[6]
sigma_med[7]
iBMI
BAgeBMI
sigmaBMI
−1.0
−0.5
0.0
0.5
1.0
Value

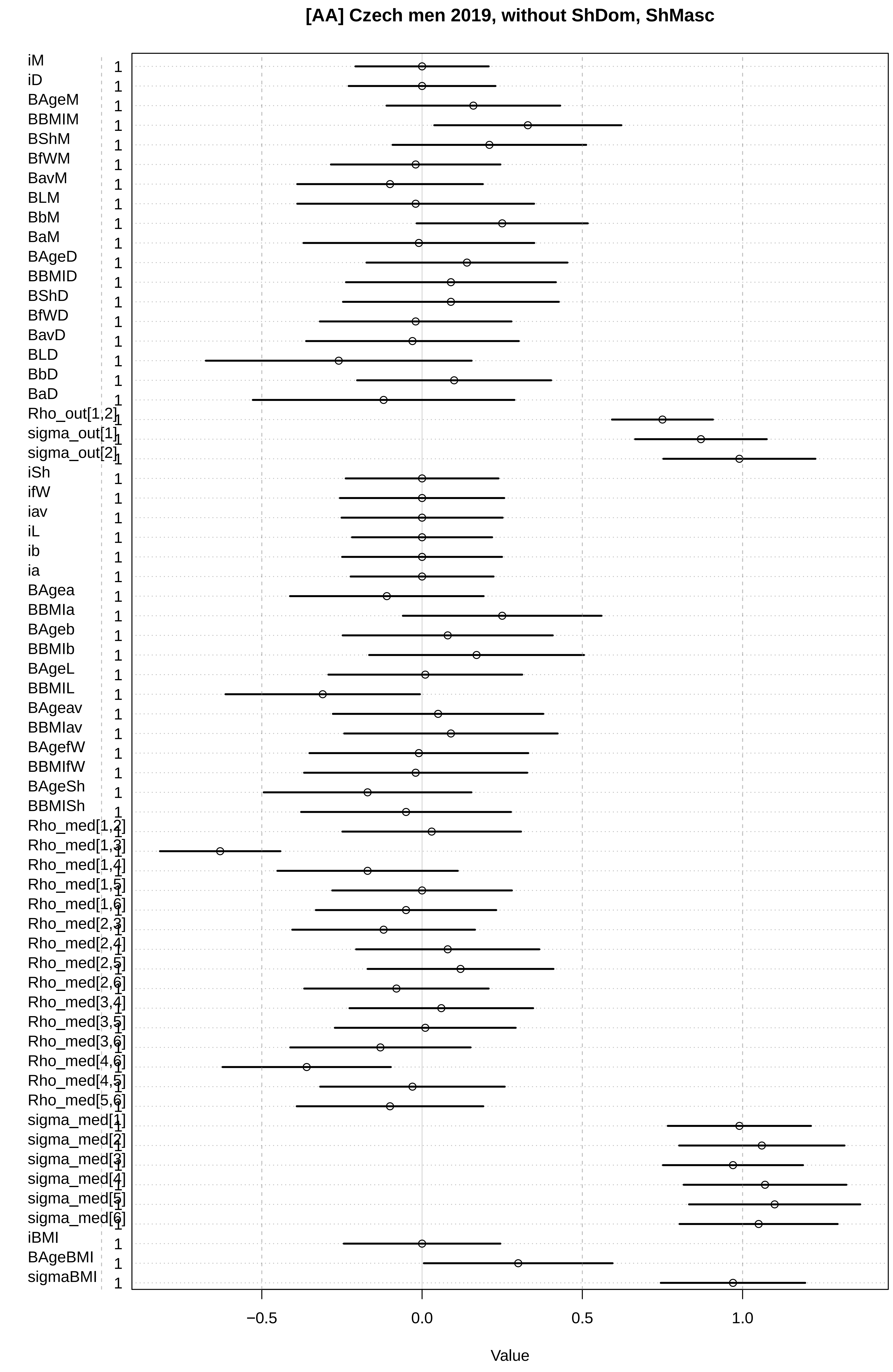
[AA] Czech men 2019, without ShDom, ShMasc
iM
iD
BAgeM
BBMIM
BShM
BfWM
BavM
BLM
BbM
BaM
BAgeD
BBMID
BShD
BfWD
BavD
BLD
BbD
BaD
Rho_out[1,2]
sigma_out[1]
sigma_out[2]
iSh
ifW
iav
iL
ib
ia
BAgea
BBMIa
BAgeb
BBMIb
BAgeL
BBMIL
BAgeav
BBMIav
BAgefW
BBMIfW
BAgeSh
BBMISh
Rho_med[1,2]
Rho_med[1,3]
Rho_med[1,4]
Rho_med[1,5]
Rho_med[1,6]
Rho_med[2,3]
Rho_med[2,4]
Rho_med[2,5]
Rho_med[2,6]
Rho_med[3,4]
Rho_med[3,5]
Rho_med[3,6]
Rho_med[4,6]
Rho_med[4,5]
Rho_med[5,6]
sigma_med[1]
sigma_med[2]
sigma_med[3]
sigma_med[4]
sigma_med[5]
sigma_med[6]
iBMI
BAgeBMI
sigmaBMI
−0.5
0.0
0.5
1.0
Value

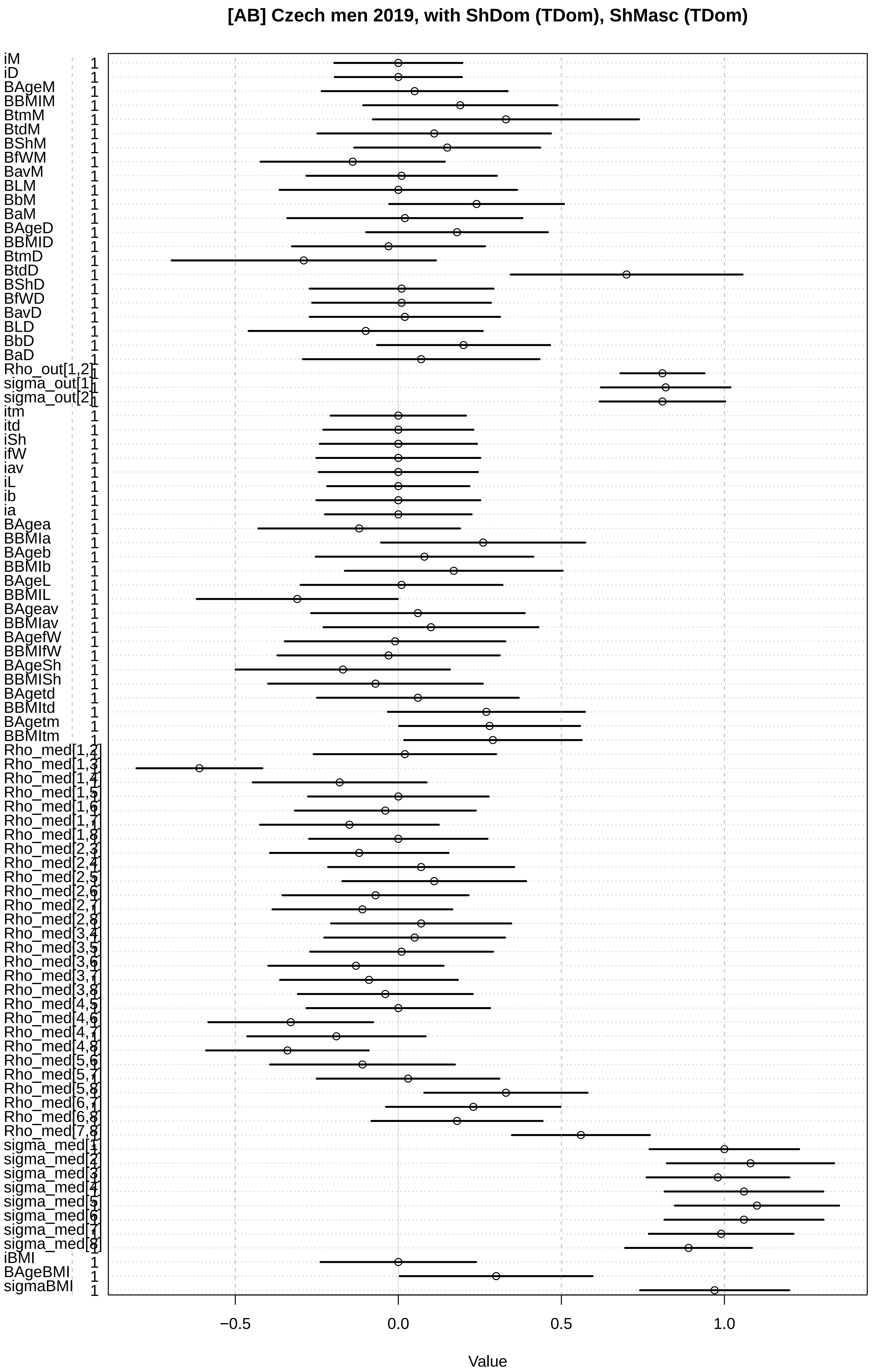
[AB] Czech men 2019, with ShDom (TDom), ShMasc (TDom)
iM
iD
BAgeM
BBMIM
BtmM
BtdM
BShM
BfWM
BavM
BLM
BbM
BaM
BAgeD
BBMID
BtmD
BtdD
BShD
BfWD
BavD
BLD
BbD
BaD
Rho_out[1,2]
sigma_out[1]
sigma_out[2]
itm
itd
iSh
ifW
iav
iL
ib
ia
BAgea
BBMIa
BAgeb
BBMIb
BAgeL
BBMIL
BAgeav
BBMIav
BAgefW
BBMIfW
BAgeSh
BBMISh
BAgetd
BBMItd
BAgetm
BBMItm
Rho_med[1,2]
Rho_med[1,3]
Rho_med[1,4]
Rho_med[1,5]
Rho_med[1,6]
Rho_med[1,7]
Rho_med[1,8]
Rho_med[2,3]
Rho_med[2,4]
Rho_med[2,5]
Rho_med[2,6]
Rho_med[2,7]
Rho_med[2,8]
Rho_med[3,4]
Rho_med[3,5]
Rho_med[3,6]
Rho_med[3,7]
Rho_med[3,8]
Rho_med[4,5]
Rho_med[4,6]
Rho_med[4,7]
Rho_med[4,8]
Rho_med[5,6]
Rho_med[5,7]
Rho_med[5,8]
Rho_med[6,7]
Rho_med[6,8]
Rho_med[7,8]
sigma_med[1]
sigma_med[2]
sigma_med[3]
sigma_med[4]
sigma_med[5]
sigma_med[6]
sigma_med[7]
sigma_med[8]
iBMI
BAgeBMI
sigmaBMI
−0.5
0.0
0.5
1.0
Value

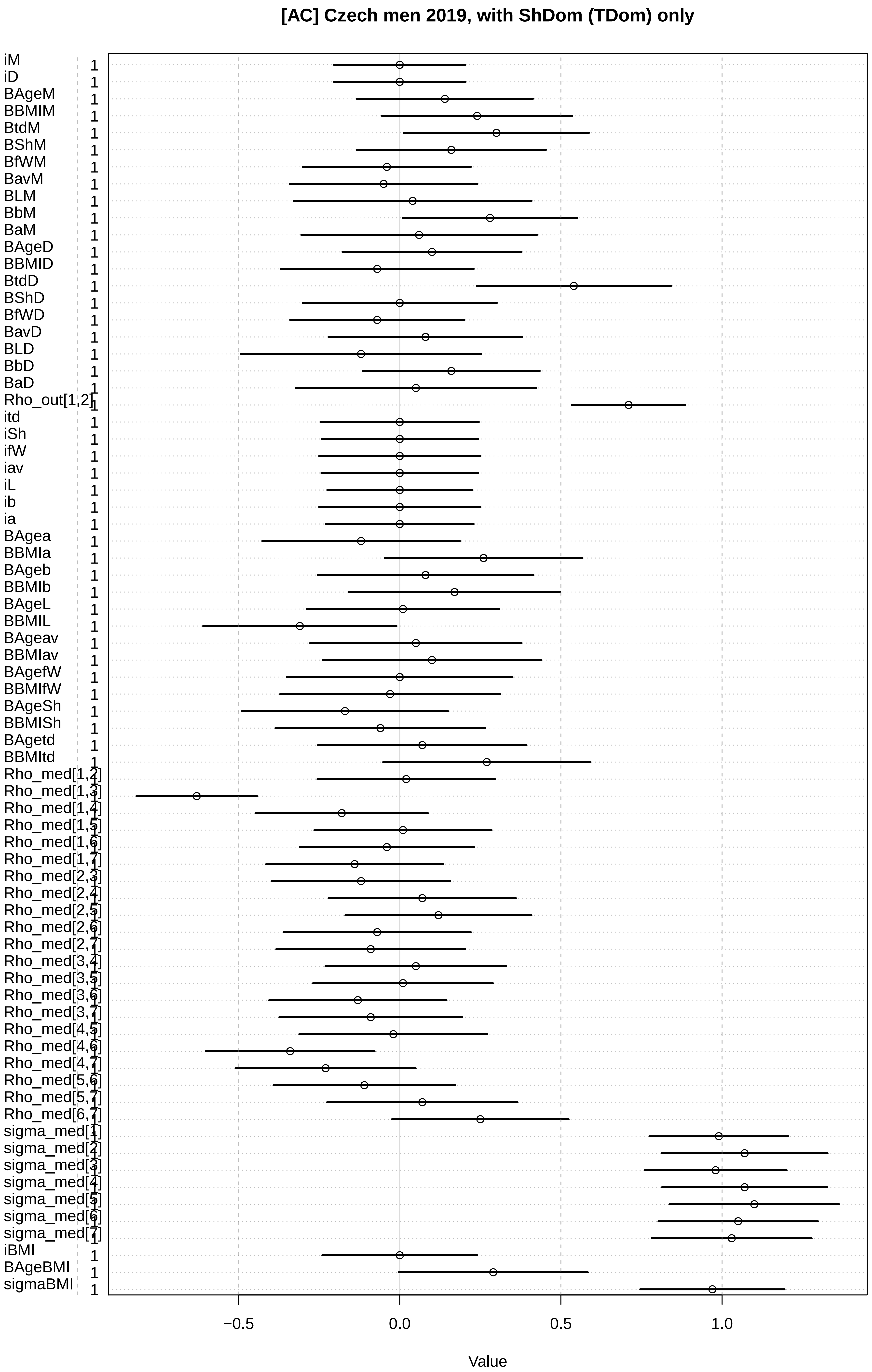

[AC] Czech men 2019, with ShDom (TDom) only
iM
iD
BAgeM
BBMIM
BtdM
BShM
BfWM
BavM
BLM
BbM
BaM
BAgeD
BBMID
BtdD
BShD
BfWD
BavD
BLD
BbD
BaD
Rho_out[1,2]
itd
iSh
ifW
iav
iL
ib
ia
BAgea
BBMIa
BAgeb
BBMIb
BAgeL
BBMIL
BAgeav
BBMIav
BAgefW
BBMIfW
BAgeSh
BBMISh
BAgetd
BBMItd
Rho_med[1,2]
Rho_med[1,3]
Rho_med[1,4]
Rho_med[1,5]
Rho_med[1,6]
Rho_med[1,7]
Rho_med[2,3]
Rho_med[2,4]
Rho_med[2,5]
Rho_med[2,6]
Rho_med[2,7]
Rho_med[3,4]
Rho_med[3,5]
Rho_med[3,6]
Rho_med[3,7]
Rho_med[4,5]
Rho_med[4,6]
Rho_med[4,7]
Rho_med[5,6]
Rho_med[5,7]
Rho_med[6,7]
sigma_med[1]
sigma_med[2]
sigma_med[3]
sigma_med[4]
sigma_med[5]
sigma_med[6]
sigma_med[7]
iBMI
BAgeBMI
sigmaBMI
−0.5
0.0
0.5
1.0
Value

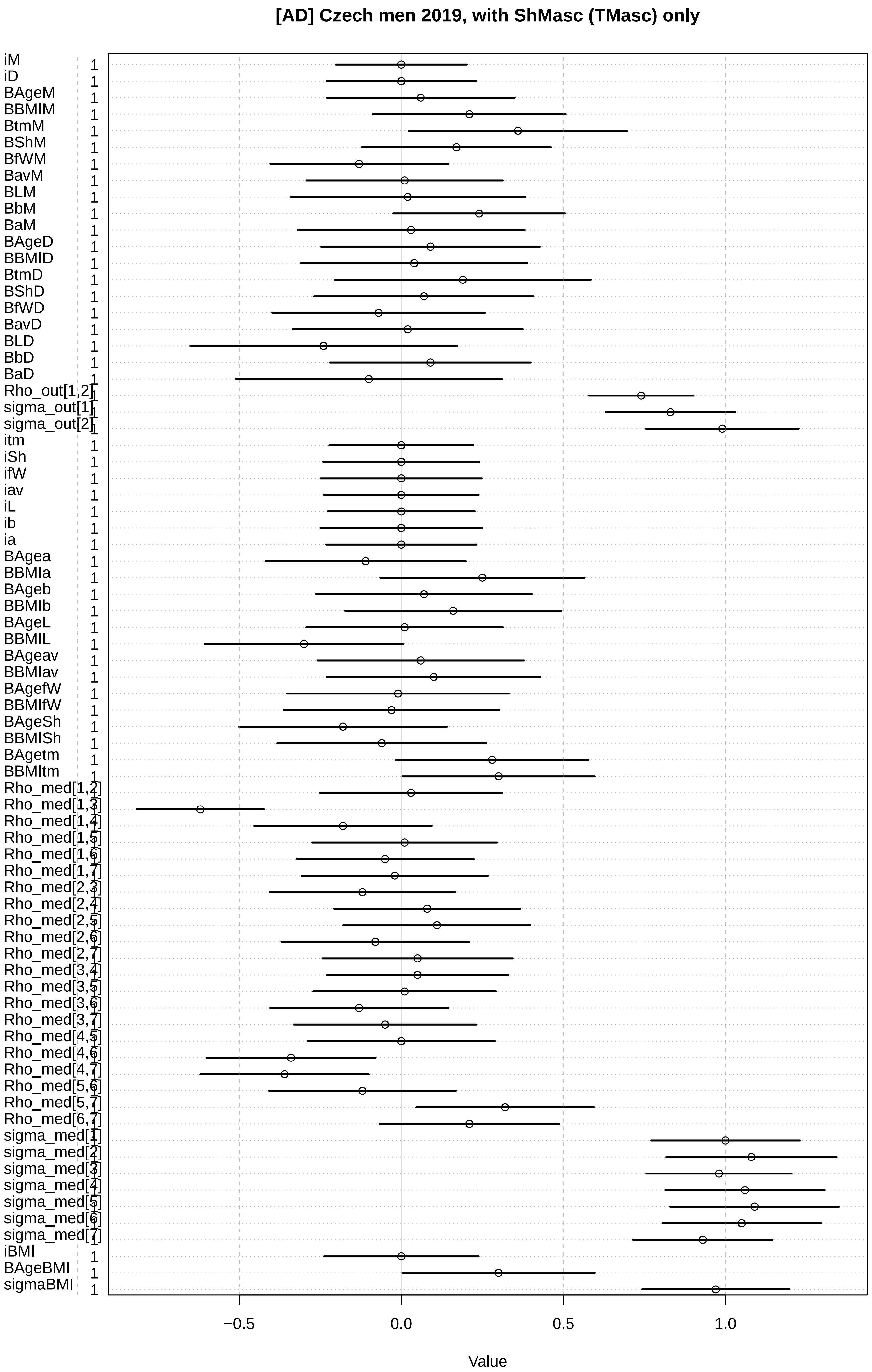

[AD] Czech men 2019, with ShMasc (TMasc) only
iM
iD
BAgeM
BBMIM
BtmM
BShM
BfWM
BavM
BLM
BbM
BaM
BAgeD
BBMID
BtmD
BShD
BfWD
BavD
BLD
BbD
BaD
Rho_out[1,2]
sigma_out[1]
sigma_out[2]
itm
iSh
ifW
iav
iL
ib
ia
BAgea
BBMIa
BAgeb
BBMIb
BAgeL
BBMIL
BAgeav
BBMIav
BAgefW
BBMIfW
BAgeSh
BBMISh
BAgetm
BBMItm
Rho_med[1,2]
Rho_med[1,3]
Rho_med[1,4]
Rho_med[1,5]
Rho_med[1,6]
Rho_med[1,7]
Rho_med[2,3]
Rho_med[2,4]
Rho_med[2,5]
Rho_med[2,6]
Rho_med[2,7]
Rho_med[3,4]
Rho_med[3,5]
Rho_med[3,6]
Rho_med[3,7]
Rho_med[4,5]
Rho_med[4,6]
Rho_med[4,7]
Rho_med[5,6]
Rho_med[5,7]
Rho_med[6,7]
sigma_med[1]
sigma_med[2]
sigma_med[3]
sigma_med[4]
sigma_med[5]
sigma_med[6]
sigma_med[7]
iBMI
BAgeBMI
sigmaBMI
−0.5
0.0
0.5
1.0
Value

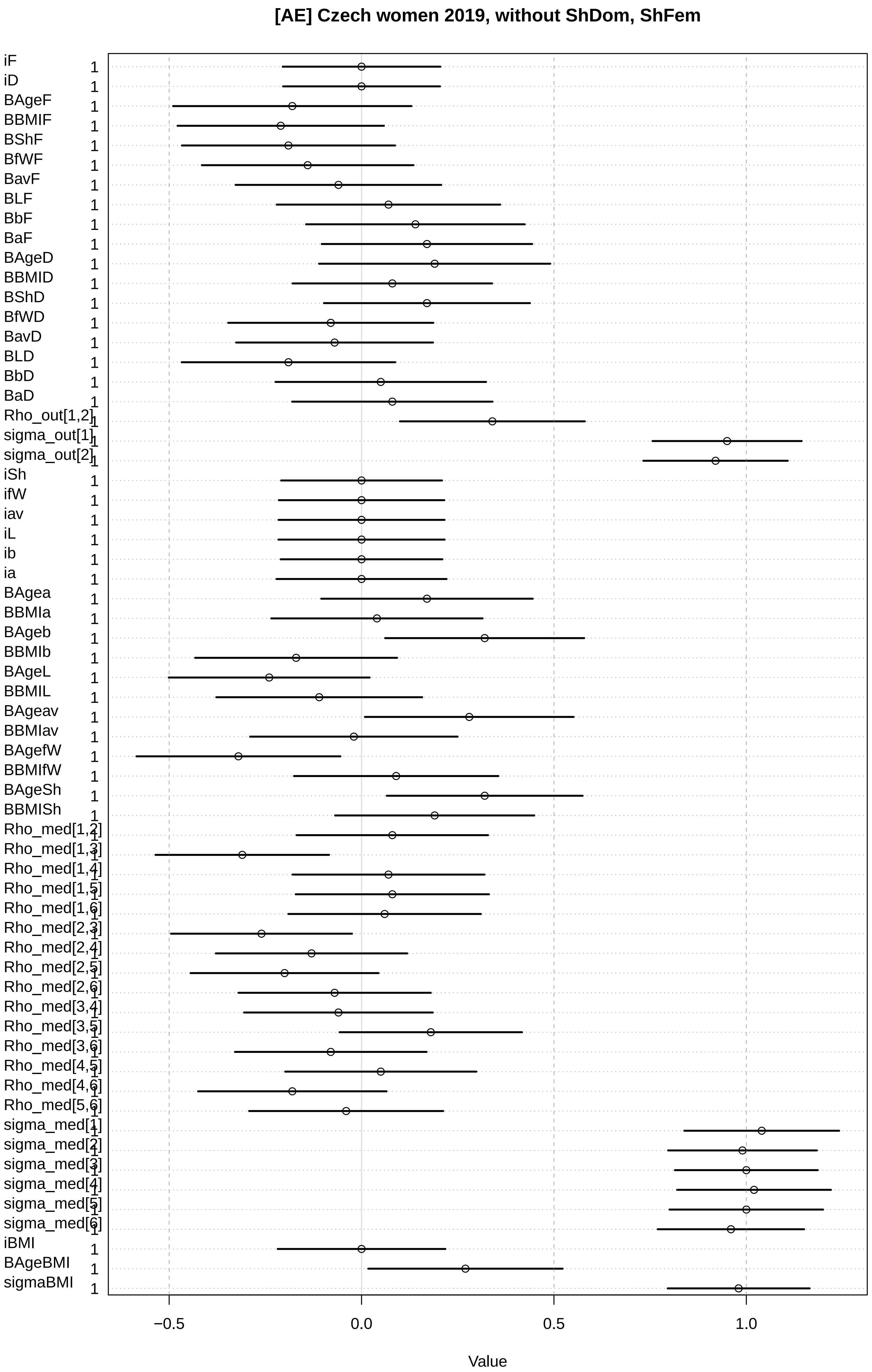
[AE] Czech women 2019, without ShDom, ShFem
iF
iD
BAgeF
BBMIF
BShF
BfWF
BavF
BLF
BbF
BaF
BAgeD
BBMID
BShD
BfWD
BavD
BLD
BbD
BaD
Rho_out[1,2]
sigma_out[1]
sigma_out[2]
iSh
ifW
iav
iL
ib
ia
BAgea
BBMIa
BAgeb
BBMIb
BAgeL
BBMIL
BAgeav
BBMIav
BAgefW
BBMIfW
BAgeSh
BBMISh
Rho_med[1,2]
Rho_med[1,3]
Rho_med[1,4]
Rho_med[1,5]
Rho_med[1,6]
Rho_med[2,3]
Rho_med[2,4]
Rho_med[2,5]
Rho_med[2,6]
Rho_med[3,4]
Rho_med[3,5]
Rho_med[3,6]
Rho_med[4,5]
Rho_med[4,6]
Rho_med[5,6]
sigma_med[1]
sigma_med[2]
sigma_med[3]
sigma_med[4]
sigma_med[5]
sigma_med[6]
iBMI
BAgeBMI
sigmaBMI
−0.5
0.0
0.5
1.0
Value

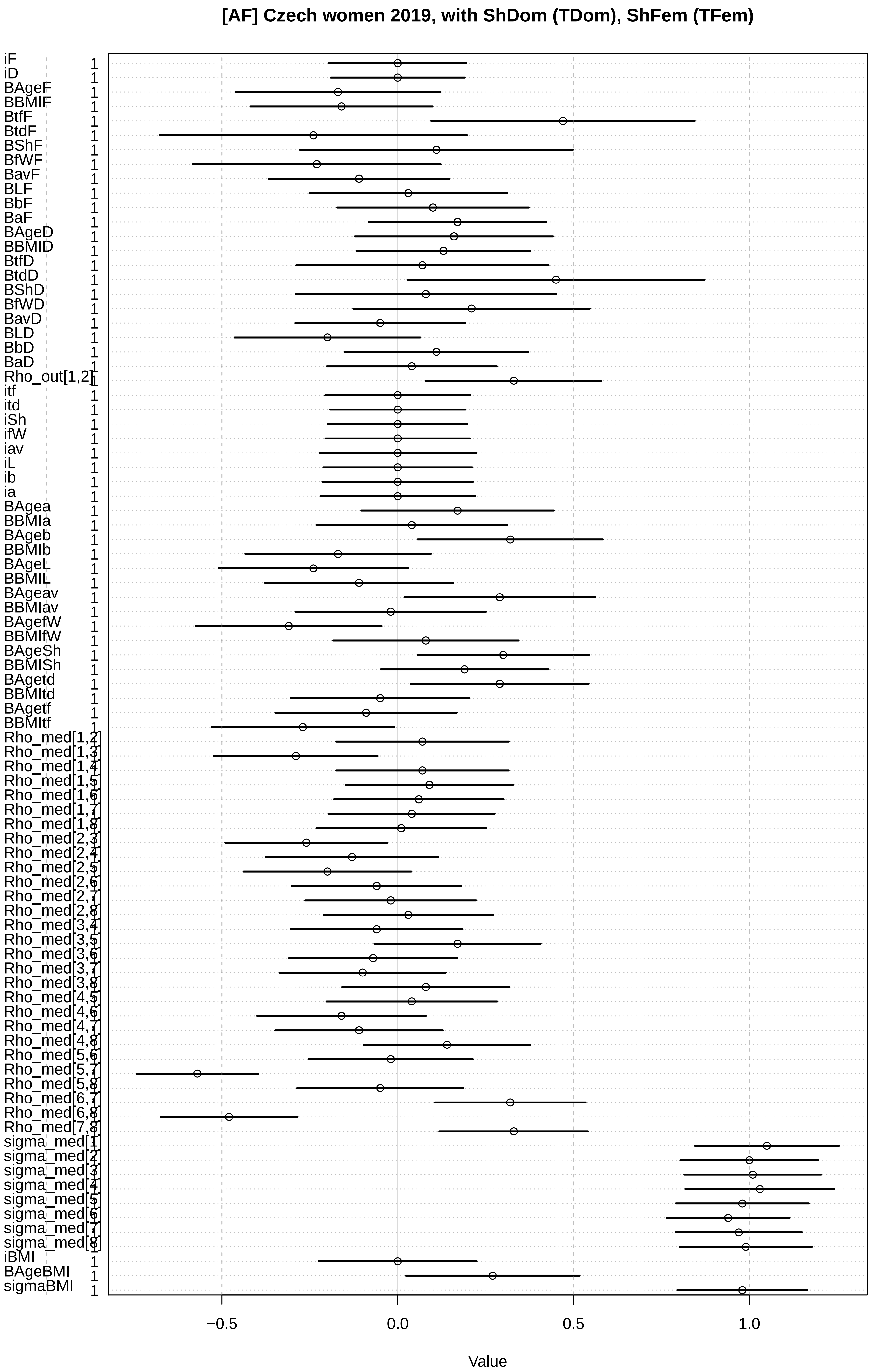
[AF] Czech women 2019, with ShDom (TDom), ShFem (TFem)
iF
iD
BAgeF
BBMIF
BtfF
BtdF
BShF
BfWF
BavF
BLF
BbF
BaF
BAgeD
BBMID
BtfD
BtdD
BShD
BfWD
BavD
BLD
BbD
BaD
Rho_out[1,2]
itf
itd
iSh
ifW
iav
iL
ib
ia
BAgea
BBMIa
BAgeb
BBMIb
BAgeL
BBMIL
BAgeav
BBMIav
BAgefW
BBMIfW
BAgeSh
BBMISh
BAgetd
BBMItd
BAgetf
BBMItf
Rho_med[1,2]
Rho_med[1,3]
Rho_med[1,4]
Rho_med[1,5]
Rho_med[1,6]
Rho_med[1,7]
Rho_med[1,8]
Rho_med[2,3]
Rho_med[2,4]
Rho_med[2,5]
Rho_med[2,6]
Rho_med[2,7]
Rho_med[2,8]
Rho_med[3,4]
Rho_med[3,5]
Rho_med[3,6]
Rho_med[3,7]
Rho_med[3,8]
Rho_med[4,5]
Rho_med[4,6]
Rho_med[4,7]
Rho_med[4,8]
Rho_med[5,6]
Rho_med[5,7]
Rho_med[5,8]
Rho_med[6,7]
Rho_med[6,8]
Rho_med[7,8]
sigma_med[1]
sigma_med[2]
sigma_med[3]
sigma_med[4]
sigma_med[5]
sigma_med[6]
sigma_med[7]
sigma_med[8]
iBMI
BAgeBMI
sigmaBMI
−0.5
0.0
0.5
1.0
Value

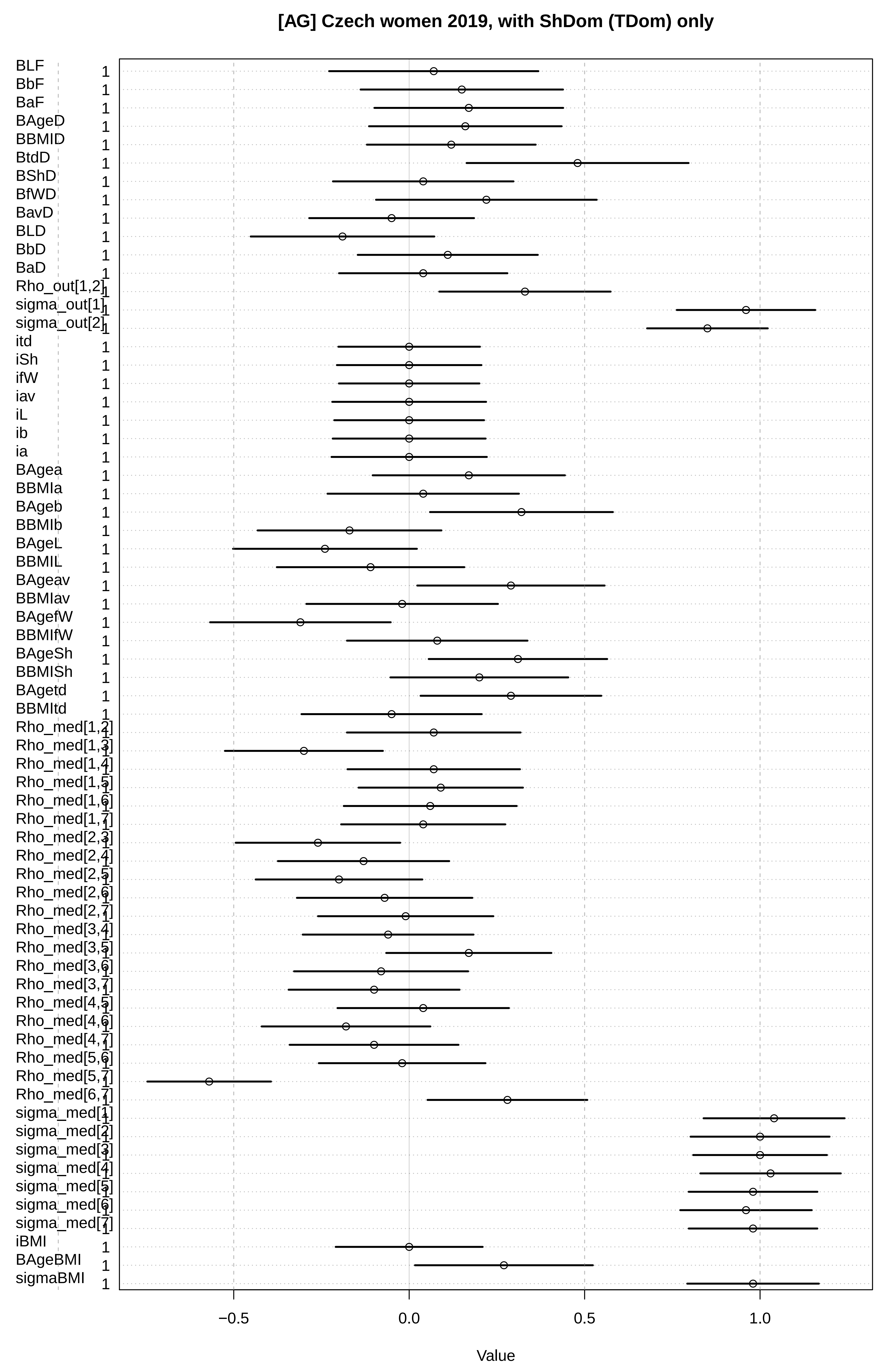

[AG] Czech women 2019, with ShDom (TDom) only
BLF
BbF
BaF
BAgeD
BBMID
BtdD
BShD
BfWD
BavD
BLD
BbD
BaD
Rho_out[1,2]
sigma_out[1]
sigma_out[2]
itd
iSh
ifW
iav
iL
ib
ia
BAgea
BBMIa
BAgeb
BBMIb
BAgeL
BBMIL
BAgeav
BBMIav
BAgefW
BBMIfW
BAgeSh
BBMISh
BAgetd
BBMItd
Rho_med[1,2]
Rho_med[1,3]
Rho_med[1,4]
Rho_med[1,5]
Rho_med[1,6]
Rho_med[1,7]
Rho_med[2,3]
Rho_med[2,4]
Rho_med[2,5]
Rho_med[2,6]
Rho_med[2,7]
Rho_med[3,4]
Rho_med[3,5]
Rho_med[3,6]
Rho_med[3,7]
Rho_med[4,5]
Rho_med[4,6]
Rho_med[4,7]
Rho_med[5,6]
Rho_med[5,7]
Rho_med[6,7]
sigma_med[1]
sigma_med[2]
sigma_med[3]
sigma_med[4]
sigma_med[5]
sigma_med[6]
sigma_med[7]
iBMI
BAgeBMI
sigmaBMI
−0.5
0.0
0.5
1.0
Value

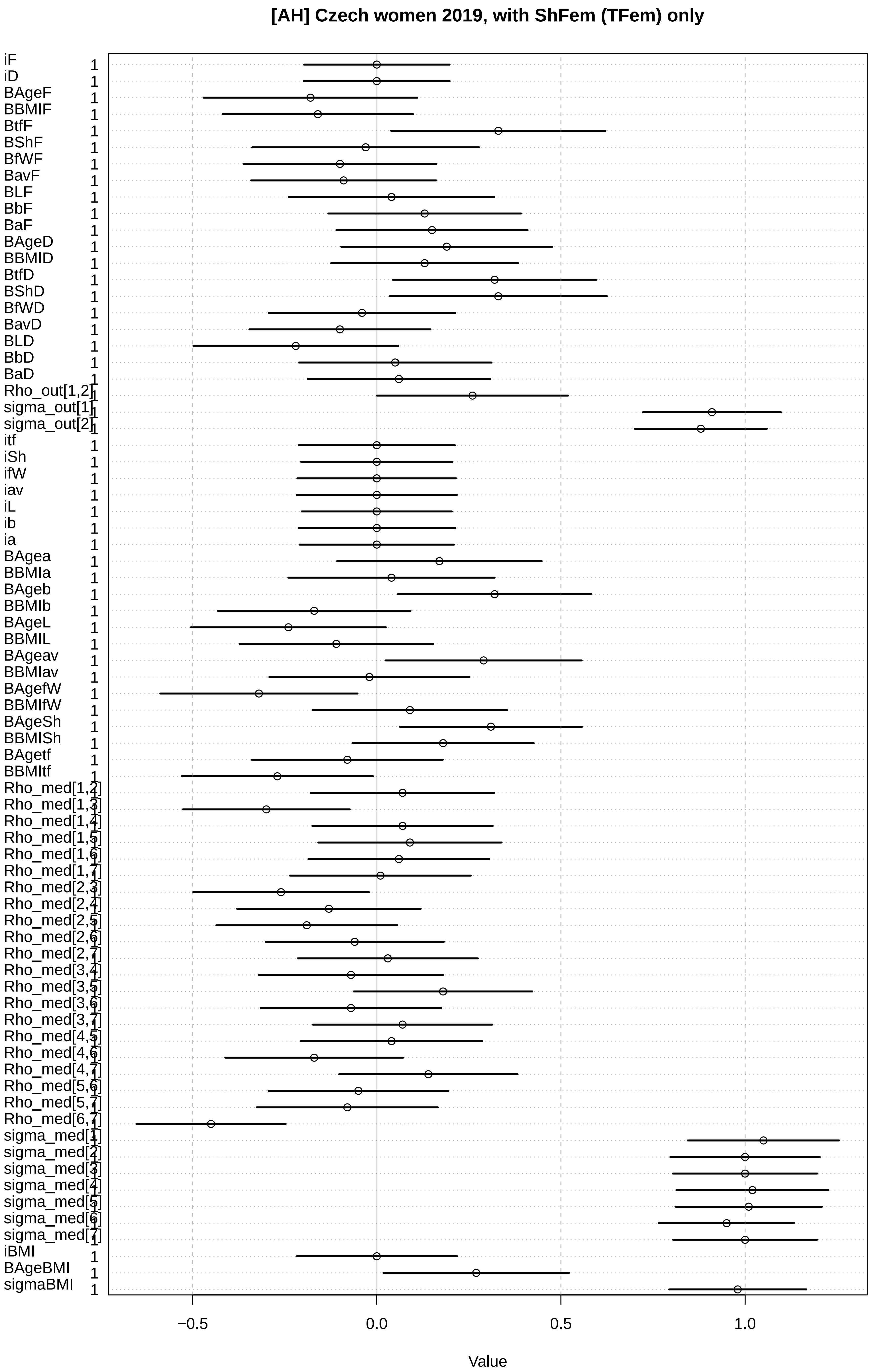

[AH] Czech women 2019, with ShFem (TFem) only
iF
iD
BAgeF
BBMIF
BtfF
BShF
BfWF
BavF
BLF
BbF
BaF
BAgeD
BBMID
BtfD
BShD
BfWD
BavD
BLD
BbD
BaD
Rho_out[1,2]
sigma_out[1]
sigma_out[2]
itf
iSh
ifW
iav
iL
ib
ia
BAgea
BBMIa
BAgeb
BBMIb
BAgeL
BBMIL
BAgeav
BBMIav
BAgefW
BBMIfW
BAgeSh
BBMISh
BAgetf
BBMItf
Rho_med[1,2]
Rho_med[1,3]
Rho_med[1,4]
Rho_med[1,5]
Rho_med[1,6]
Rho_med[1,7]
Rho_med[2,3]
Rho_med[2,4]
Rho_med[2,5]
Rho_med[2,6]
Rho_med[2,7]
Rho_med[3,4]
Rho_med[3,5]
Rho_med[3,6]
Rho_med[3,7]
Rho_med[4,5]
Rho_med[4,6]
Rho_med[4,7]
Rho_med[5,6]
Rho_med[5,7]
Rho_med[6,7]
sigma_med[1]
sigma_med[2]
sigma_med[3]
sigma_med[4]
sigma_med[5]
sigma_med[6]
sigma_med[7]
iBMI
BAgeBMI
sigmaBMI
−0.5
0.0
0.5
1.0
Value
